# Integrative Single-Cell Transcriptomic, Mendelian Randomization and In Silico Perturbation Analyses Prioritize MUC20 as a Candidate Gene Associated with Osteoporosis and Metabolic Dysfunction-Associated Steatotic Liver Disease in the Liver–Bone Axis

**DOI:** 10.3390/ijms27125453

**Published:** 2026-06-16

**Authors:** Hui Jin, Xiangting Ye, Gonghui Jian, Hui Xiong

**Affiliations:** 1 School of Integrated Chinese and Western Medicine, Hunan University of Chinese Medicine, Changsha 410208, China; 2 Graduate School, Hunan University of Chinese Medicine, Changsha 410208, China

**Keywords:** metabolic dysfunction-associated steatotic liver disease, osteoporosis, single-cell transcriptomics, mendelian randomization, in silico perturbation

## Abstract

Metabolic dysfunction-associated steatotic liver disease (MASLD) and osteoporosis (OP) are epidemiologically linked, but shared cell-type-specific molecular features remain unclear. We integrated public single-cell/single-nucleus transcriptomic datasets for OP (GSE147287) and MASLD (GSE289173) with two-sample Mendelian randomization (MR), colocalization, network annotation, macrophage-focused in silico perturbation, and exploratory serum assessment. After quality control, 13,753 OP cells and 42,438 MASLD cells/nuclei were analyzed. Macrophages were consistently identified in both datasets and showed disease-associated expansion. Directionally concordant macrophage differentially expressed genes yielded 147 shared candidate genes, with enrichment mainly involving lipid/sterol metabolism, extracellular matrix and adhesion processes, immune presentation, lysosomal processing, and phagocytic pathways. MR prioritized *MUC20* as the only candidate with concordant odds ratios greater than 1 for both OP (OR = 1.044, 95% CI 1.003–1.086) and MASLD (OR = 1.111, 95% CI 1.038–1.189). Colocalization supported shared genetic signals for *MUC20* in OP (PP.H4 = 0.855) and MASLD (PP.H4 = 0.816). In silico perturbation suggested a limited but pathway-enriched predicted transcriptional footprint. Serum MUC20 was higher in patients with OP+MASLD than in healthy controls. This integrative analysis identified shared macrophage-associated transcriptional themes and prioritized *MUC20* as a candidate molecule for future liver–bone crosstalk studies.

## 1. Introduction

Metabolic dysfunction-associated steatotic liver disease (MASLD) and osteoporosis (OP) are common metabolic disorders affecting the liver and skeleton, respectively, and both impose substantial health burdens worldwide. Large systematic reviews estimate that MASLD affects nearly one-third of the global adult population [1,2]. OP affects approximately 200 million people worldwide and contributes to approximately 8.9 million osteoporotic fractures each year [3]. Among adults aged 50 years or older, fracture risk increases markedly with age and remains an important cause of disability and mortality [4]. Observational studies and systematic reviews suggest that MASLD is associated with lower bone mineral density and a higher risk of OP or fractures [5,6]. However, the strength and direction of this association vary across studies, likely because of differences in population characteristics, metabolic comorbidities, and disease definitions [7]. These inconsistencies highlight the need for evidence with higher cellular resolution and complementary genetic support.

Metabolic stress and chronic low-grade inflammation are key features of both MASLD progression and abnormal bone remodeling [8,9]. Myeloid-lineage cells, especially monocytes and macrophages, regulate metabolic inflammation, local tissue remodeling, and immune responses, and may provide a biologically relevant interface between hepatic inflammatory niches and skeletal microenvironmental changes [10,11]. However, much of the current evidence comes from phenotypic associations or bulk transcriptomic studies, which cannot clearly distinguish changes in cell composition from transcriptional changes within specific cell types [12]. Single-cell and single-nucleus transcriptomics can resolve disease-associated molecular changes at the level of individual cell types and cell states, providing a useful approach for identifying cross-disease cellular programs [13]. Recent multi-omics studies in MASLD have also shown coordinated changes in hepatic metabolic, inflammatory, and tissue-remodeling networks, further supporting the value of integrative omics approaches [14].

Observational associations are also limited by confounding and reverse causation, which makes directional interpretation difficult. Mendelian randomization (MR) can provide complementary genetic evidence by using genetic variants as instruments to evaluate exposure–outcome relationships [15]. Although MASLD–OP associations and liver–bone immunometabolic crosstalk have been increasingly discussed, shared myeloid programs and genetically supported candidate genes remain insufficiently defined. Here, we integrated cell-type-resolved single-cell evidence, MR-based genetic prioritization, colocalization analysis, network annotation, computational perturbation, and exploratory serum assessment to identify shared macrophage-related programs and prioritize candidate genes for subsequent validation.

## 2. Results

### 2.1. Single-Cell Analysis in Osteoporosis

**Figure 1:**
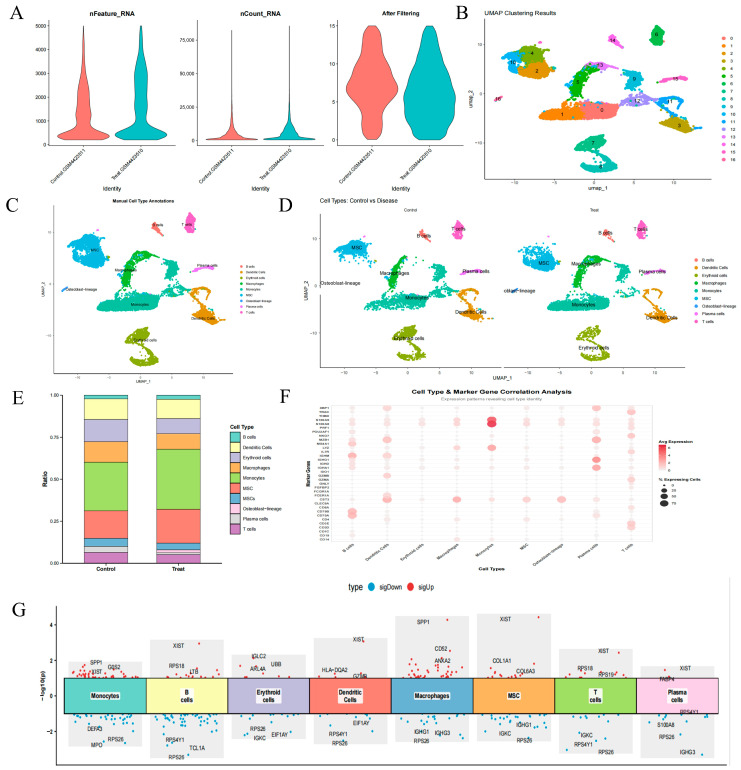
Single-cell analysis of osteoporosis (OP). (**A**) Quality control assessment after filtering. (**B**) UMAP dimensionality reduction and clustering. (**C**) Cell-type annotation based on canonical marker genes. (**D**) Cell-type distribution in control and OP samples. (**E**) Cell-type proportions in control and OP samples. (**F**) Dot plot showing representative marker gene expression across annotated cell types. (**G**) Differentially expressed genes across cell types in OP versus control samples.

In the OP dataset (GSE147287), low-quality cells were removed using the following quality control thresholds: nFeature_RNA 200–5000, nCount_RNA 500–86,000, and percent.mt < 15%. After filtering, 13,753 cells were retained, including 6549 control cells and 7204 OP cells. Post-filtering metrics were within the expected ranges: nFeature_RNA 201–4997, nCount_RNA 520–85,588, and percent.mt 0–14.995% (Figure 1A).

Using 2000 highly variable genes, we performed principal component analysis (PCA) and selected 13 principal components for downstream analysis. These components explained 91.23% of the cumulative variance. Unsupervised clustering at a resolution of 0.4 identified 17 clusters, and uniform manifold approximation and projection (UMAP) visualization showed separation of the major clusters (Figure 1B). Based on cluster-specific marker genes, canonical lineage markers, and CellMarker 2.0 reference information [16], we annotated nine major cell types: monocytes, macrophages, dendritic cells, B cells, T cells, plasma cells, erythroid cells, mesenchymal stem/progenitor cells (MSCs), and osteoblast-lineage cells (Figure 1C). Projection of cell-type labels onto the UMAP showed the distribution of these lineages in control and OP samples (Figure 1D). DotPlot analysis further supported the marker-based annotation of the major cell populations (Figure 1F).

Cell composition analysis showed a higher proportion of macrophages in OP than in controls (25.02% vs. 18.17%) and a lower proportion of osteoblast-lineage cells (35.27% vs. 42.52%) (both *p* < 0.05; Figure 1E). These results suggested disease-related changes in myeloid and bone-forming cell populations. Cell-type-stratified differential expression analysis also showed transcriptional differences across lineages, as summarized by the distribution of upregulated and downregulated genes in each cell type (Figure 1G). These findings provided the basis for subsequent cross-disease comparison.

### 2.2. Single-Cell Analysis in MASLD

**Figure 2:**
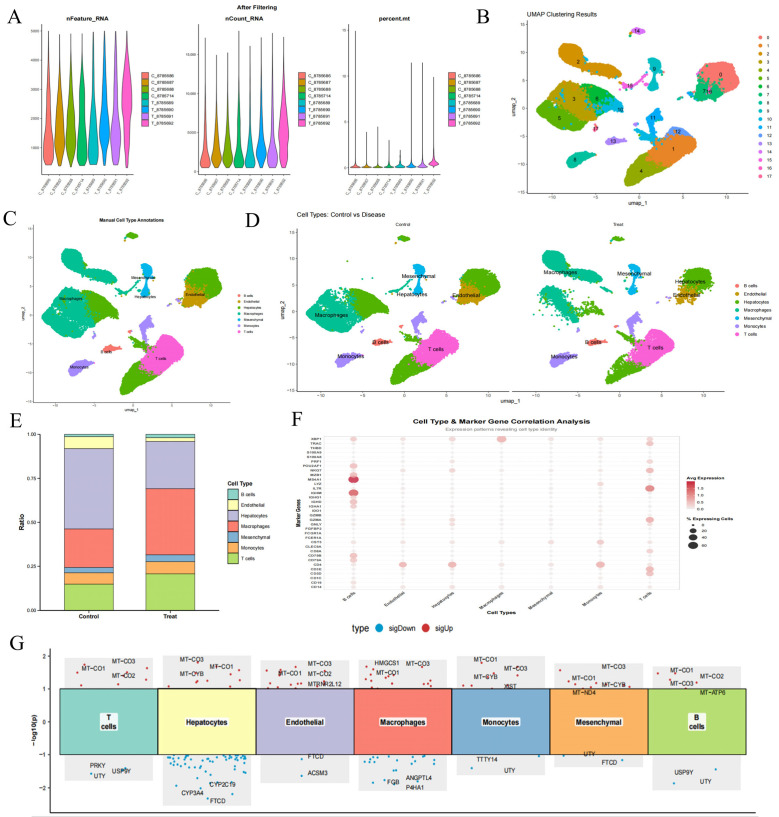
Single-cell analysis of metabolic dysfunction-associated steatotic liver disease. (**A**) Quality control assessment after filtering. (**B**) UMAP dimensionality reduction and clustering. (**C**) Cell-type annotation based on canonical marker genes. (**D**) Cell-type distribution in control and MASLD samples. (**E**) Cell-type proportions in control and MASLD samples. (**F**) Dot plot showing representative marker gene expression across annotated cell types. (**G**) Differentially expressed genes across cell types in MASLD versus control samples.

In the MASLD dataset (GSE289173), eight samples were merged and filtered using the following criteria: nFeature_RNA 300–5000, nCount_RNA 499–18,023, and percent.mt < 15%. After filtering, 42,438 cells/nuclei were retained, including 22,805 control cells/nuclei and 19,633 MASLD cells/nuclei. Post-filtering metrics were nFeature_RNA 313–4999, nCount_RNA 502–18,023, and percent.mt 0–14.91% (Figure 2A).

PCA was performed using 2000 highly variable genes, and 12 principal components were selected for downstream analysis. These components explained 91.46% of the cumulative variance. Unsupervised clustering identified 18 clusters, and UMAP visualization showed separation of the major clusters (Figure 2B). Using canonical marker genes and CellMarker 2.0 reference information, we annotated seven major cell types: hepatocytes, endothelial cells, mesenchymal cells, monocytes, macrophages, T cells, and B cells (Figure 2C,D). DotPlot visualization showed the expected lineage-specific marker expression patterns and supported the cell-type annotation (Figure 2F).

Cell composition analysis showed a higher macrophage fraction in MASLD than in controls (37.59% vs. 28.98%) and a lower hepatocyte fraction (48.95% vs. 63.57%) (both *p* < 0.05; Figure 2E). These results suggested disease-related myeloid enrichment and parenchymal cell reduction. Cell-type-stratified differential expression analysis further showed transcriptional differences across immune and parenchymal lineages (Figure 2G).

### 2.3. Shared Candidate Genes Between Osteoporosis and MASLD

To compare transcriptional features across diseases, we focused on macrophages. This myeloid lineage was consistently annotated in both datasets and showed disease-associated expansion in both OP and MASLD. Because the OP and MASLD datasets came from different tissue contexts, disease-control differential expression analyses were performed separately within each dataset. We then compared directionally concordant macrophage differentially expressed genes (DEGs) at the gene-set level. Within macrophages, DEGs were identified using the same criteria in both datasets: false discovery rate (FDR) < 0.05 and |log_2_FC| > 0.5.

After stratifying DEGs by direction and intersecting gene sets across diseases, we identified 76 commonly downregulated genes (MASLD: 1662; OP: 864; Figure 3A) and 71 commonly upregulated genes (MASLD: 688; OP: 2457; Figure 3B). These 147 shared candidate genes were used for downstream functional annotation and genetic prioritization. Macrophage subcluster marker expression supporting the macrophage-lineage annotation is shown in Supplementary Figure S1.

**Figure 3:**
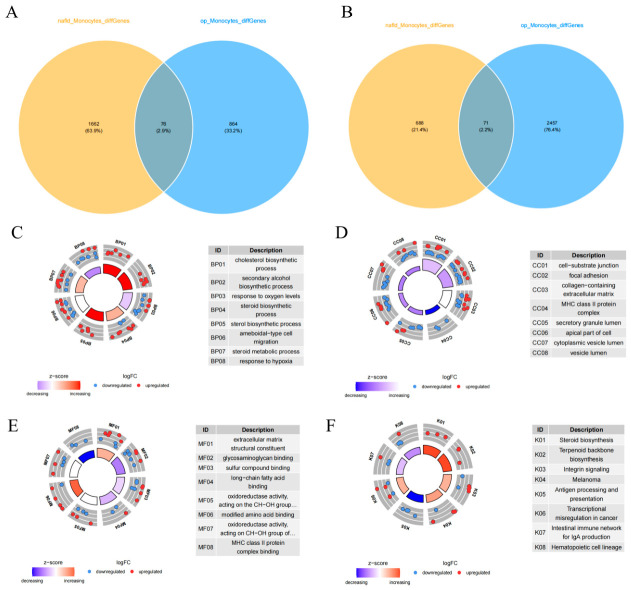
Shared macrophage-associated candidate genes and functional enrichment between MASLD and OP. (**A**) Venn diagram showing the overlap of downregulated macrophage DEGs between MASLD and OP. (**B**) Venn diagram showing the overlap of upregulated macrophage DEGs between MASLD and OP. (**C**–**E**) GO enrichment of the 147 shared candidate genes across biological process (**C**), cellular component (**D**), and molecular function (**E**), shown by GOCircle plots. (**F**) KEGG pathway enrichment of the shared candidate genes.

Functional enrichment analysis of the 147 shared candidates showed that Gene Ontology (GO) biological process terms were mainly related to cholesterol, sterol, and steroid biosynthesis, responses to oxygen levels or hypoxia, and amoeboidal-type cell migration (Figure 3C). GO cellular component terms included cell–substrate junctions, focal adhesions, collagen-containing extracellular matrix, and vesicle or granule lumen-related structures (Figure 3D). GO molecular function terms included extracellular matrix structural constituents, glycosaminoglycan binding, long-chain fatty acid binding, and redox-related activities (Figure 3E). Kyoto Encyclopedia of Genes and Genomes (KEGG) enrichment included steroid biosynthesis, terpenoid backbone biosynthesis, integrin signaling, antigen processing and presentation, intestinal immune network for immunoglobulin A production, and hematopoietic cell lineage (Figure 3F). Overall, these enrichment results suggested overlapping functional annotations related to lipid or sterol metabolism, extracellular matrix and adhesion processes, and immune-presentation pathways.

### 2.4. Mendelian Randomization and Colocalization Prioritize MUC20 as a Candidate Gene

**Figure 4:**
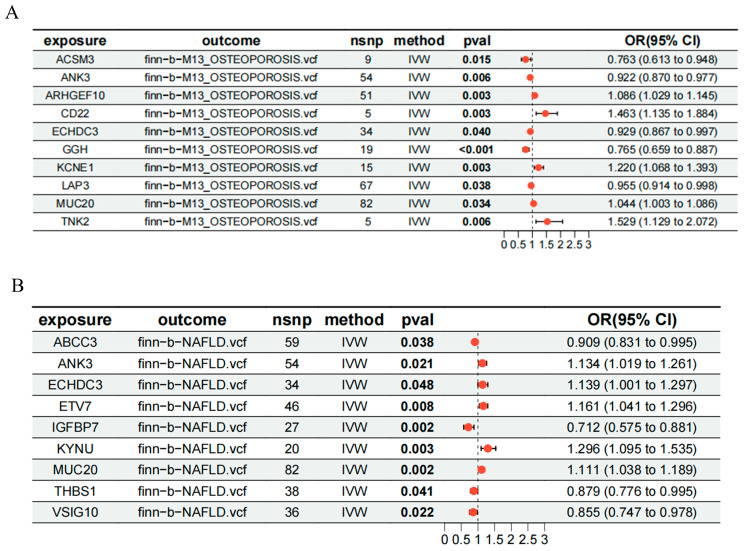
Forest plot of IVW-significant gene-level MR estimates for OP and MASLD. (**A**) Gene-level MR estimates for OP. (**B**) Gene-level MR estimates for MASLD. Estimates were obtained using IVW as the primary MR method. Points indicate ORs and horizontal lines indicate 95% CIs.

Using expression quantitative trait locus (eQTL)-based instruments derived from shared macrophage candidate genes, we performed two-sample MR. Inverse-variance weighting (IVW) was used as the primary estimator. The IVW analysis identified 10 genes associated with osteoporosis (OP) and nine genes associated with MASLD at *p* < 0.05 (Figure 4A,B). The corresponding IVW estimates and robustness diagnostics, including MR-Egger intercept and heterogeneity tests, are summarized in Table 1.

Among these genes, *MUC20* was the only candidate with concordant odds ratios (ORs) > 1 for both OP and MASLD. The IVW estimate was OR = 1.044 for OP (95% confidence interval [CI] 1.003–1.086; *p* = 0.034; nSNP = 82) and OR = 1.111 for MASLD (95% CI 1.038–1.189; *p* = 0.002; nSNP = 82). Sensitivity analyses showed similar effect directions across complementary MR methods. Leave-one-out analyses did not suggest that the estimates were driven by a single variant. Detailed single-nucleotide polymorphism (SNP)-level robustness analyses for *MUC20* are provided in Supplementary Figure S2. Retained instrument characteristics are provided in Supplementary Tables S1–S4.

**Figure 5:**
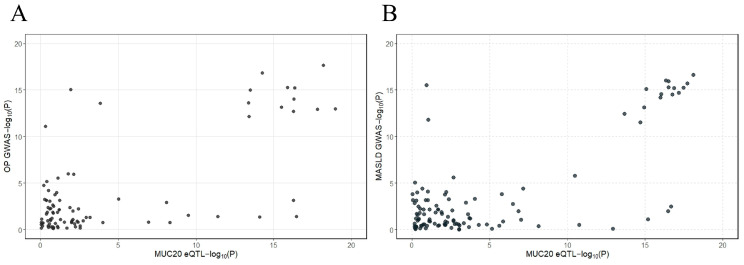
Colocalization analysis of *MUC20* in OP and MASLD. (**A**) Scatter plot comparing *MUC20* eQTL significance and OP GWAS significance across shared SNPs. (**B**) Scatter plot comparing *MUC20* eQTL significance and MASLD GWAS significance across shared SNPs. The x-axis indicates *MUC20* eQTL −log_10_(*p*), and the y-axis indicates disease GWAS −log_10_(*p*).

We further performed colocalization analysis to assess whether *MUC20* eQTL and disease genome-wide association study (GWAS) signals shared genetic signals. For OP, the posterior probability supporting a shared causal variant was PP.H4 = 0.855, whereas the probability supporting distinct causal variants was PP.H3 = 0.014. For MASLD, PP.H4 was 0.816 and PP.H3 was 0.022 (Figure 5; Table 2). Together, these MR and colocalization results prioritized *MUC20* for subsequent functional interpretation in OP–MASLD comorbidity.

### 2.5. Network-Based Functional Annotation of MUC20

**Figure 6:**
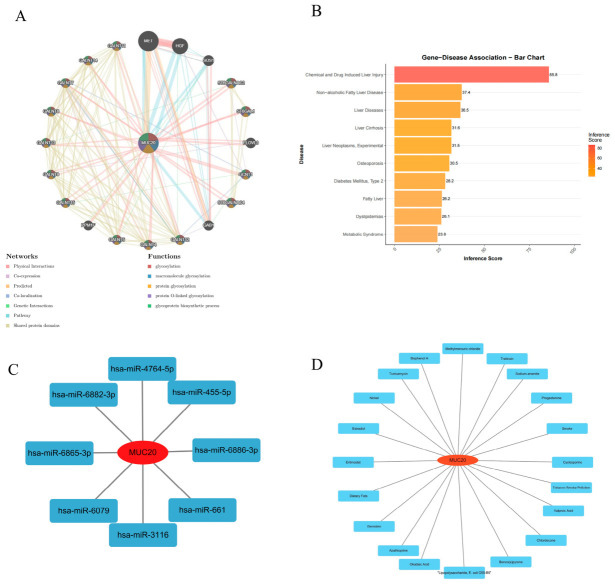
Network-based and knowledge-based characterization of *MUC20*. (**A**) GeneMANIA interaction network of *MUC20* and its functional neighborhood, highlighting glycosylation-related modules. (**B**) Gene–disease associations for *MUC20* curated from the CTD. (**C**) Predicted miRNA regulators of *MUC20* from miRDB. (**D**) Gene–chemical associations for *MUC20* from CTD. These analyses provide functional and translational context for subsequent hypothesis testing.

After MR prioritization of *MUC20*, we performed multi-source network annotation to describe its functional context. GeneMANIA placed *MUC20* within a network involving MET, HGF, SOS1, and several glycosylation-related genes. The enriched functions were mainly related to glycosylation and protein O-linked glycosylation (Figure 6A). Comparative Toxicogenomics Database (CTD) annotations linked *MUC20* to liver injury, metabolic liver disease, OP, and metabolic phenotypes (Figure 6B). miRDB predictions and CTD chemical association data provided additional database-derived information on possible upstream regulatory and environmental contexts (Figure 6C,D). Overall, these database-derived observations provided functional context for *MUC20* and helped define hypotheses for future experimental evaluation.

### 2.6. In Silico Perturbation Suggests Candidate Lysosomal, Efferocytosis-Related, and Osteoclast-Associated Programs

**Figure 7:**
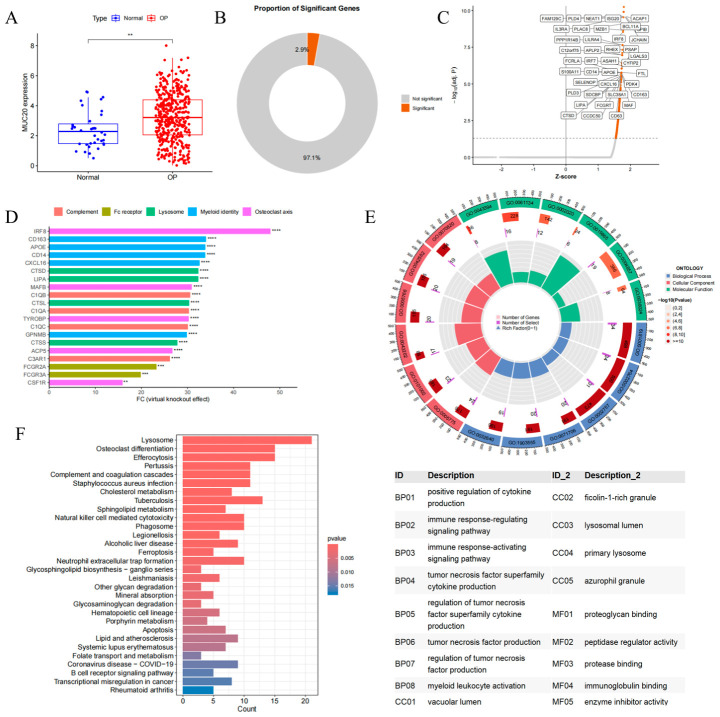
Macrophage-focused in silico perturbation of *MUC20* in osteoporosis and predicted downstream transcriptional programs. (**A**) *MUC20* expression in macrophages from control and OP samples. (**B**) Proportion of significantly altered genes following simulated *MUC20* perturbation. (**C**) Distribution of predicted downstream transcriptional changes after *MUC20* perturbation, with representative genes labeled. (**D**) Top predicted downstream genes ranked by perturbation effect size and grouped by functional modules. (**E**) GO enrichment of perturbation-associated genes. (**F**) KEGG enrichment of perturbation-associated genes. ** *p* < 0.01, *** *p* < 0.001, **** *p* < 0.0001.

To explore candidate downstream transcriptional programs associated with *MUC20*, we performed macrophage-focused in silico perturbation of *MUC20* in OP macrophages. At the single-cell level, *MUC20* expression was higher in disease macrophages than in control macrophages (Figure 7A). After simulated perturbation, a small fraction of genes reached statistical significance, accounting for approximately 2.9% of analyzed genes (Figure 7B). Representative predicted downstream genes included ACAP1, SPIB, JCHAIN, BCL11A, IRF8, LILRA4, PLD4, FAM129C, NEAT1, ISG20, and IL3RA (Figure 7C). High-effect predicted downstream genes were mainly annotated to modules related to the complement/Fc receptor axis, lysosome–phagocytosis, myeloid identity, and osteoclast-associated pathways (Figure 7D).

Enrichment analysis of perturbation-associated genes showed related functional patterns. Gene Ontology (GO) biological process terms included positive regulation of cytokine production, immune response regulation and activation, and myeloid leukocyte activation. GO cellular component terms included vacuolar lumen, lysosomal lumen, primary lysosome, and azurophil granule. GO molecular function terms included proteoglycan binding, peptidase regulation or binding, and immunoglobulin binding (Figure 7E). KEGG pathways included lysosome, osteoclast differentiation, efferocytosis, complement and coagulation cascades, phagosome, sphingolipid metabolism, cholesterol metabolism, and natural killer cell-mediated cytotoxicity (Figure 7F). Overall, the in silico perturbation analysis indicated a limited but pathway-enriched predicted transcriptional footprint associated with *MUC20*. These results support further functional assessment of *MUC20* in macrophage-related models of OP–MASLD comorbidity.

### 2.7. Exploratory Serum Assessment of MUC20 in the Clinical Cohort

A total of 52 participants were included in this exploratory clinical cohort, including 20 healthy controls and 32 patients with OP+MASLD. Baseline characteristics are summarized in Table 3. Age and body mass index (BMI) were comparable between the two groups. The OP+MASLD group had a lower lumbar spine T-score and higher levels of alanine aminotransferase (ALT), aspartate aminotransferase (AST), triglycerides (TG), and total cholesterol (TC) than healthy controls. These differences were consistent with the skeletal and metabolic liver disease features of the cohort.

**Figure 8:**
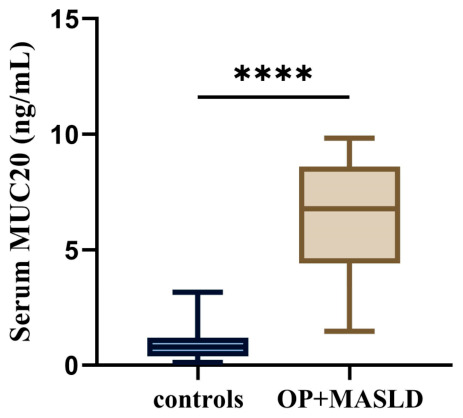
Comparison of serum MUC20 levels between the two groups. **** *p* < 0.0001.

Serum MUC20 levels were measured by enzyme-linked immunosorbent assay (ELISA). The mean serum MUC20 level was 1.02 ± 0.90 in healthy controls and 6.37 ± 2.57 in the OP+MASLD group. In this two-group exploratory comparison, serum MUC20 levels were higher in patients with OP+MASLD than in healthy controls (*p* < 0.001; Figure 8). This finding was consistent with the bioinformatics prioritization of MUC20 at the serum level.

## 3. Discussion

The association between MASLD and low bone mass or OP has been reported in epidemiological studies and systematic reviews. However, this relationship is difficult to interpret because obesity, insulin resistance, systemic inflammation, and other metabolic comorbidities are closely linked to both conditions. The direction of this association also remains uncertain [17]. In this study, we examined MASLD and OP at cellular and molecular levels. We focused on macrophages as a shared myeloid context and assessed whether the two diseases showed directionally concordant transcriptional features. We then used genetic and computational analyses to prioritize candidate molecules for further study [6,7].

Previous genetic studies have suggested shared genetic architecture or possible directional relationships between MASLD-related traits and bone outcomes, including bone mineral density (BMD), OP, and fractures. However, their conclusions have varied because of differences in phenotype definitions, instrument selection, outcome datasets, and adjustment strategies [18]. In some analyses, effect estimates were attenuated after adjustment for body mass index or glycemic traits, suggesting that the MASLD–bone relationship may be embedded in a broader metabolic and inflammatory network rather than driven by a single pathway [19]. Therefore, our study aimed to identify shared myeloid transcriptional features at cell-type resolution and to use genetic evidence to prioritize candidate genes for downstream validation [20].

Myeloid cells are important regulators of metabolic stress, inflammation, and tissue remodeling. Single-cell studies of MASLD and metabolic dysfunction-associated steatohepatitis (MASH) have described hepatic macrophage states related to lipid handling, inflammation, repair, and fibrosis [21]. TREM2-associated lipid-associated macrophage programs have also been linked to immune homeostasis under metabolic stress and fibrotic niche remodeling [22,23]. In addition, lysosomal lipid load, efferocytosis, and inflammation-resolution capacity have been proposed as key myeloid functions in steatohepatitis progression and regression [24,25,26]. In bone, myeloid-lineage cells give rise to osteoclasts and also contribute to immune regulation and tissue remodeling [27,28]. Changes in bone-resident macrophages and monocyte-lineage states may affect the balance between bone resorption and formation, especially under inflammatory conditions [29]. These findings support the use of macrophages as a relevant cell type for cross-disease comparison.

Within this matched macrophage lineage, we identified 147 directionally concordant genes between MASLD and OP. Because the datasets differed in tissue source, cell composition, metabolic environment, and matrix structure, this shared gene set should not be interpreted as evidence of identical macrophage states across tissues. Instead, it may reflect overlapping myeloid transcriptional themes under chronic metabolic stress and tissue remodeling [30,31]. Functional annotation of these genes highlighted lipid handling, lysosomal processing, phagocytic clearance, inflammatory regulation, extracellular matrix remodeling, and adhesion-related pathways [28]. These pathways are biologically plausible in both liver injury and bone remodeling contexts, but they should be viewed as functional annotations rather than disease-specific mechanisms.

We next used two-sample MR to add a genetic layer to the transcriptomic findings. Unlike previous MR studies that mainly tested relationships between MASLD phenotypes and bone outcomes [32], our analysis used MR to help prioritize genes emerging from shared macrophage-associated modules [33]. Following Strengthening the Reporting of Observational Studies in Epidemiology using Mendelian Randomization (STROBE-MR) recommendations [34], we combined inverse variance-weighted estimates with sensitivity analyses, heterogeneity and pleiotropy tests, and *MUC20*-focused colocalization. *MUC20* was the only gene with concordant odds ratios greater than 1 for both outcomes in the primary analyses. Colocalization further supported shared genetic signals between *MUC20* expression and both OP and MASLD. These findings support *MUC20* as a candidate gene for further functional evaluation, but they do not establish a large biological effect or a confirmed disease mechanism [35].

We then used macrophage-focused in silico perturbation to explore the functional context of *MUC20*. This analysis was intended to identify predicted downstream programs, not to validate a mechanism [36]. The perturbation footprint was modest, with approximately 2.9% of genes reaching statistical significance. The predicted changes were mainly annotated to myeloid programs related to phagocytosis, lysosomal processing, inflammatory responses, and osteoclast differentiation. This pattern was consistent with the enrichment results from the shared candidate genes and suggested a possible link between *MUC20* and macrophage-related clearance or remodeling programs [26]. However, in silico perturbation depends on model assumptions and data coverage. Future experiments should test *MUC20* knockdown or overexpression in macrophages and assess efferocytosis, lysosomal acidification, lipid load, inflammatory mediator production, and osteoclastogenic capacity [37].

*MUC20* is a transmembrane mucin family member that has been associated with receptor signaling and stress-related cellular responses. Transmembrane mucins can participate in barrier function, adhesion, and immune-response regulation through their glycosylated extracellular domains and cytoplasmic tails [20]. Previous studies have suggested a possible relationship between *MUC20* and hepatocyte growth factor (HGF)/c-MET signaling, a pathway involved in liver injury, repair, inflammation, and chemotaxis [38,39,40]. The cyclic GMP-AMP synthase–stimulator of interferon genes (cGAS–STING) axis may also provide a stress inflammation interface in metabolic disease contexts [41]. Public expression resources suggest that baseline *MUC20* expression is generally low in peripheral immune cells and may be tissue- or cell-type-specific [42]. Therefore, the macrophage-associated signal observed here may reflect disease-related expression changes or enrichment of specific macrophage states. Independent replication and tissue-level localization are needed. Overall, these findings suggest that *MUC20* may be linked to macrophage-associated signaling and clearance programs in MASLD–OP comorbidity, but this hypothesis requires direct testing in myeloid systems and animal models [43,44,45].

From a cross-organ perspective, MASLD is increasingly viewed as a systemic metabolic condition. Its associations with low bone mass, OP, and fracture risk have been described in reviews and meta-analyses, although effect sizes vary across populations and metabolic subgroups [46]. Current liver–bone axis concepts suggest that hepatokines, inflammatory mediators, and lipid-metabolic stress may affect the balance between bone formation and resorption, while bone-derived signals may also influence hepatic metabolism [47]. In this context, our analysis provides a candidate molecular entry point centered on macrophage-associated programs and *MUC20*, complementing studies based mainly on phenotypic associations [48]. Outputs from the Comparative Toxicogenomics Database and GeneMANIA should be interpreted as database-derived functional context rather than evidence of clinical efficacy [49]. A practical next step is to test whether *MUC20* perturbation affects macrophage immune-metabolic phenotypes, followed by in vivo assessment of liver pathology and bone mass or remodeling endpoints [50,51]. In the exploratory clinical cohort, serum MUC20 levels were higher in patients with OP+MASLD than in healthy controls. This finding was consistent with the bioinformatics prioritization of *MUC20*, but it should be interpreted as preliminary serum-level evidence.

Several limitations should be considered. First, the public single-cell datasets differed in donor scale, disease definition, tissue source, and sequencing strategy [30]. Cross-tissue comparison cannot fully remove the influence of tissue niche, sample composition, and platform differences [31]. Second, the shared candidate gene set was derived from cell-level differential expression analysis. Because cells from the same donor are not fully independent observations, this approach may introduce pseudoreplication, and some identified genes may reflect donor or sample imbalance rather than genuine disease-associated transcriptional changes [52]. Future studies should validate the shared macrophage signatures using donor-level pseudobulk or mixed-model frameworks in independent cohorts [53]. Third, MR inference remains affected by instrument validity, linkage disequilibrium structure, expression quantitative trait locus tissue context, and assumptions about horizontal pleiotropy. Although we used sensitivity analyses, heterogeneity and pleiotropy tests, and *MUC20*-focused colocalization, residual bias cannot be excluded. Fourth, in silico perturbation provides computational evidence only and cannot replace experimental knockdown or overexpression. Fifth, the exploratory serum analysis was based on a single-center, two-group cohort that included healthy controls and patients with OP+MASLD. Because OP-only and MASLD-only groups were not included, we could not determine the relative contributions of OP, MASLD, combined disease status, metabolic abnormalities, systemic inflammation, medication exposure, or other clinical covariates. Larger and better-characterized cohorts are needed. Finally, cell–cell communication analysis and spatial localization may help determine whether *MUC20*-associated myeloid states are enriched in fibrotic liver niches or bone remodeling sites.

In summary, we identified shared macrophage-associated immunometabolic transcriptional themes between MASLD and OP and prioritized *MUC20* as a candidate gene using complementary evidence from MR, colocalization, and computational perturbation. These findings provide a basis for future studies of *MUC20*-related myeloid programs in MASLD–OP comorbidity. Further work should replicate the shared macrophage module in independent and spatially resolved datasets and test the functional relevance of *MUC20* in efferocytosis, lysosomal homeostasis, inflammatory output, and bone remodeling-related macrophage functions.

## 4. Materials and Methods

### 4.1. Data Sources and Study Design

**Figure 9:**
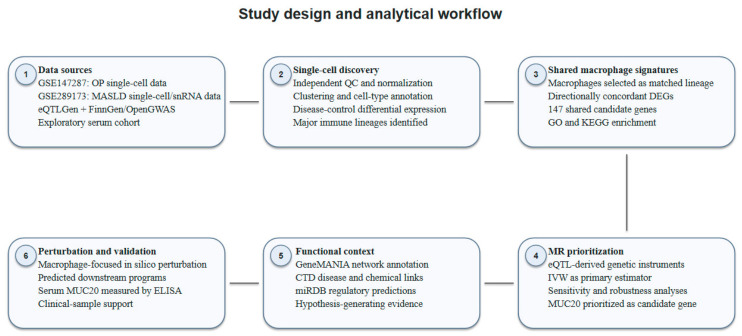
Study design and analytical workflow.

The overall study design and analytical workflow are summarized in Figure 9. Single-cell transcriptomic data for OP and MASLD were obtained from the Gene Expression Omnibus (GEO; https://www.ncbi.nlm.nih.gov/geo/; accessed on 18 January 2026) under accession numbers GSE147287 and GSE289173, respectively [54,55,56]. We used an integrative framework that combined cell-type-resolved single-cell analysis, two-sample MR, colocalization analysis, network-based functional annotation, and macrophage-focused computational perturbation [57,58].

Each single-cell dataset was processed independently for quality control, cell-type annotation, and cell-type-stratified differential expression analysis [12]. Shared candidate genes were identified within immune lineages present in both diseases. eQTL instruments for candidate genes were used as exposures, and GWAS summary statistics for OP and MASLD were used as outcomes in two-sample MR analyses [59,60,61]. For MR-prioritized genes, we performed network-based annotation and assessed predicted downstream transcriptional effects in macrophages [62]. This study used publicly available de-identified single-cell and summary-level genetic data, together with a single-center exploratory serum cohort. No intervention was performed, and the clinical component was limited to peripheral blood sampling.

### 4.2. Single-Cell Data Processing

The OP single-cell dataset GSE147287 and the MASLD single-cell or single-nucleus dataset GSE289173 were processed independently in R version 4.2.2 (R Foundation for Statistical Computing, Vienna, Austria) using Seurat version 5.4.0. Seurat objects were generated from raw count matrices. Standard quality control (QC) metrics were calculated, including the number of detected features (nFeature_RNA), total counts (nCount_RNA), and the percentage of mitochondrial transcripts (percent.mt). Dataset-specific QC thresholds were selected based on metric distributions to remove low-quality cells or nuclei and outliers with abnormally high counts.

Data were normalized using LogNormalize with a scale factor of 10,000. Highly variable genes were selected using the variance-stabilizing transformation method, and the top 2000 features were retained. The data were then scaled, and PCA was performed. For multi-sample datasets, batch effects were addressed using Seurat’s anchor-based integration workflow, including FindIntegrationAnchors and IntegrateData. A shared nearest-neighbor (SNN) graph was constructed in the integrated space, and clusters were identified using Louvain community detection. Clusters were visualized using UMAP. Cell types were annotated using canonical marker expression and cluster-specific marker genes.

### 4.3. Differential Expression and Functional Enrichment Analyses

Within each major cell type or subpopulation, disease-control differential expression analysis was performed using Seurat’s FindMarkers function with the Wilcoxon rank-sum test. Multiple testing was controlled using the Benjamini–Hochberg procedure. DEGs were defined as genes with an FDR-adjusted *p*-value < 0.05 and |log_2_ fold change| > 0.5.

To identify cross-disease candidate signatures, we first aligned cell-type labels between datasets. The analysis was then restricted to immune lineages present in both diseases, mainly macrophages, monocytes, T cells, and B cells. For each matched lineage, DEGs were stratified as upregulated or downregulated. Directionally concordant DEGs were intersected across diseases. The union of common upregulated and common downregulated genes was defined as the shared candidate gene set. Functional enrichment analysis was performed using clusterProfiler for GO biological process, cellular component, and molecular function terms, as well as KEGG pathway analysis [63,64,65]. Enrichment significance was defined as FDR-adjusted *p* < 0.05.

### 4.4. Two-Sample Mendelian Randomization and Colocalization Analyses

Two-sample MR analyses were performed in R version 4.2.2 using the TwoSampleMR package version 0.6.29. Genetic instruments were derived from candidate gene-related eQTL associations obtained from eQTLGen (https://www.eqtlgen.org/; accessed on 18 January 2026) and OpenGWAS (https://opengwas.io/; accessed on 18 January 2026) [61]. GWAS summary statistics for OP and MASLD were obtained from FinnGen/OpenGWAS resources (FinnGen: https://www.finngen.fi/en; OpenGWAS: https://opengwas.io/; all accessed on 18 January 2026). The OpenGWAS eQTL identifiers ranged from eqtl-a-ENSG00000000003 to eqtl-a-ENSG00000270149.

Single-nucleotide polymorphisms (SNPs) associated with gene expression at genome-wide significance were selected using *p* < 5 × 10^−8^. To reduce linkage disequilibrium (LD) among instruments, clumping was performed using an *r^2^* threshold of 0.001 within a 10,000 kb window. Instrument strength was assessed using the *F-*statistic, and only instruments with *F-*statistics > 10 were retained. Exposure and outcome datasets were harmonized to ensure consistent effect–allele orientation [59]. Detailed SNP-level information, including exposure and outcome effect estimates, standard errors, effect allele frequencies, allele alignment, palindromic status, and *F-*statistics, is provided in the Supplementary Tables. The primary MR estimate was obtained using IVW and reported as ORs with 95% CIs. Robustness was evaluated using complementary MR estimators, including MR-Egger, weighted median, simple mode, and weighted mode, with emphasis on consistency in effect direction. Potential bias was assessed using Cochran’s Q test for heterogeneity, the MR-Egger intercept for horizontal pleiotropy, and leave-one-out analysis.

Bayesian colocalization analysis was performed using the coloc package version 5.2.3 in R to assess whether *MUC20* expression and disease associations shared genetic signals. Shared SNPs between the *MUC20* eQTL dataset and the corresponding OP or MASLD GWAS dataset were aligned and analyzed. Posterior probabilities were calculated for five hypotheses: no association with either trait (H0), association with *MUC20* expression only (H1), association with disease only (H2), association with both traits through distinct causal variants (H3), and association with both traits through a shared causal variant (H4). PP.H4 was used to assess support for a shared genetic signal between *MUC20* expression and each disease outcome.

### 4.5. Network-Based Functional Annotation of Prioritized Genes

Network- and knowledge-based annotation of MR-prioritized genes was performed using GeneMANIA (https://genemania.org/; accessed on 18 January 2026), miRDB (https://mirdb.org/; accessed on 18 January 2026), and the Comparative Toxicogenomics Database (CTD; http://ctdbase.org/; accessed on 18 January 2026). Gene–gene interaction networks were constructed using GeneMANIA to describe functional neighborhoods [62]. High-confidence microRNA–messenger RNA regulatory relationships were predicted using miRDB [66]. Gene–disease and gene–chemical associations were extracted from CTD [49]. These analyses were used to provide database-derived functional context for prioritized genes and to support hypothesis generation.

### 4.6. In Silico Perturbation of MUC20 and Downstream Effect Assessment

Macrophage-focused in silico perturbation of MUC20 was performed using the scTenifoldKnk framework version 1.0.2 [36]. The OP disease-state macrophage subset was used as the perturbation context. Raw count data were extracted from the Seurat object, and highly variable genes were identified using the variance-stabilizing transformation method. The input matrix included the top 20,000 highly variable genes and *MUC20*. *MUC20* was retained during feature filtering to ensure inclusion of the perturbation target. Quality control within the perturbation workflow used a mitochondrial transcript threshold of 0.1 and a minimum library size of 1000. Genes detected in at least 5% of cells were retained when the number of cells exceeded 500. *MUC20* was retained regardless of this filtering criterion. Gene regulatory networks were reconstructed using 10 subsampled networks with 500 cells per network, *q* = 0.9, and three principal components. Tensor decomposition was performed with *K* = 3, a maximum of 1000 iterations, and a convergence error threshold of 1 × 10^−5^. *MUC20* perturbation was simulated by setting the *MUC20* regulatory row to zero in the reconstructed network, followed by manifold alignment between the original and perturbed networks. Differentially regulated genes after *MUC20* perturbation were identified from the scTenifoldKnk differential regulation output. Genes with adjusted *p* < 0.05 were considered perturbation-associated downstream genes. The proportion of significant genes was calculated relative to all genes included in the perturbation output after excluding *MUC20* itself. Perturbation-associated genes were then used for GO and KEGG enrichment analyses with Benjamini–Hochberg correction. This analysis was used to prioritize candidate downstream transcriptional programs associated with *MUC20* perturbation.

### 4.7. Exploratory Serum Assessment Using Clinical Samples

To provide exploratory serum-level support for the bioinformatics findings, postmenopausal women consecutively recruited from the Second Affiliated Hospital of Hunan University of Chinese Medicine between December 2025 and March 2026 were enrolled. Participants were assigned to an OP+MASLD group or a healthy control group. OP was defined as a lumbar spine T-score ≤ −2.5 on dual-energy X-ray absorptiometry (DXA). MASLD was diagnosed based on imaging-confirmed hepatic steatosis with concomitant metabolic abnormalities. Individuals with other definite chronic liver diseases, malignancy, acute or chronic infection, severe cardiac or renal dysfunction, or recent use of medications that could substantially affect bone or lipid metabolism were excluded.

Peripheral venous blood was collected in the morning after overnight fasting. Serum was separated by centrifugation and stored at −80 °C until analysis. Serum MUC20 levels were measured using a Human *MUC20* QuickTest ELISA Kit (FineTest, Wuhan, China, Cat. No. QT-EH1227) according to the manufacturer’s instructions. All samples were assayed in duplicate. Laboratory personnel were blinded to group assignment, and measurements were performed in the same batch whenever possible. The study was approved by the Ethics Committee of the Second Affiliated Hospital of Hunan University of Chinese Medicine before participant enrollment and sample collection (approval no. 2024-KY03; approval date: 15 March 2024). Written informed consent was obtained from all participants before enrollment.

### 4.8. Statistical Analysis and Software

Single-cell analyses, enrichment analyses, and MR analyses were performed in R version 4.2.2. Figure assembly was completed using Adobe Illustrator 2023. Clinical data and serum MUC20 levels from the exploratory cohort were also analyzed in R. Continuous variables are presented as mean ± standard deviation (SD) or median (interquartile range [IQR]), as appropriate. Normality was assessed using the Shapiro–Wilk test. Between-group comparisons were performed using Student’s *t* test or the Mann–Whitney U test, as appropriate. Categorical variables were compared using the chi-square test or Fisher’s exact test, where applicable. Multiple testing was controlled using FDR-adjusted *p*-values for differential expression and enrichment analyses. Unless otherwise specified, all tests were two-sided, and *p* < 0.05 was considered statistically significant.

## 5. Conclusions

Using an integrative framework combining cell-type-resolved transcriptomics, two-sample Mendelian randomization, colocalization analysis, and macrophage-focused computational perturbation, we identified shared macrophage-associated immunometabolic transcriptional themes between metabolic dysfunction-associated steatotic liver disease (MASLD) and osteoporosis (OP). *MUC20* was prioritized as a candidate gene associated with both outcomes. Exploratory serum assessment by enzyme-linked immunosorbent assay (ELISA) showed higher circulating *MUC20* levels in patients with OP+MASLD than in healthy controls. Overall, these findings support *MUC20* as a candidate molecule for future studies of liver–bone crosstalk, with further experimental and clinical validation needed to clarify its functional relevance.

## Figures and Tables

**Table 1:** Summary of gene-level MR estimates and robustness diagnostics for OP and MASLD.

Gene	Outcome	nSNP	IVW OR	95% CI	IVW *p*-Value	MR-Egger Intercept	MR-Egger Intercept *p*-Value	Heterogeneity *p*-Value
*ACSM3*	OP	9	0.763	0.613–0.948	0.0149	−0.0302	0.2676	0.5757
*ANK3*	OP	54	0.922	0.870–0.977	0.0061	−0.0144	0.1700	0.8992
*ARHGEF10*	OP	51	1.086	1.029–1.145	0.0026	0.0041	0.6843	0.4687
*CD22*	OP	5	1.463	1.135–1.884	0.0033	0.0037	0.9017	0.9042
*ECHDC3*	OP	34	0.929	0.867–0.997	0.0401	−0.0104	0.3907	0.9676
*GGH*	OP	19	0.765	0.659–0.887	0.0004	0.0200	0.4288	0.8960
*KCNE1*	OP	15	1.220	1.068–1.393	0.0035	0.0063	0.6985	0.6754
*LAP3*	OP	67	0.955	0.914–0.998	0.0384	0.0056	0.5452	0.6946
*MUC20*	OP	82	1.044	1.003–1.086	0.0344	0.0056	0.4499	0.1506
*TNK2*	OP	5	1.529	1.129–2.072	0.0061	0.0147	0.7745	0.9893
*ABCC3*	MASLD	59	0.909	0.831–0.995	0.0377	−0.0108	0.5566	0.9680
*ANK3*	MASLD	54	1.134	1.019–1.261	0.0213	−0.0226	0.2422	0.9388
*ECHDC3*	MASLD	34	1.139	1.001–1.297	0.0483	−0.0111	0.6224	0.4608
*ETV7*	MASLD	46	1.161	1.041–1.296	0.0075	−0.0118	0.5768	0.4864
*IGFBP7*	MASLD	27	0.712	0.575–0.881	0.0018	−0.0246	0.4940	0.2447
*KYNU*	MASLD	20	1.296	1.095–1.535	0.0026	0.0420	0.2028	0.7332
*MUC20*	MASLD	82	1.111	1.038–1.189	0.0024	0.0141	0.2685	0.9742
*THBS1*	MASLD	38	0.879	0.776–0.995	0.0415	−0.0155	0.4696	0.5772
*VSIG10*	MASLD	36	0.855	0.747–0.978	0.0225	0.0013	0.9549	0.5811

Note: Genes shown in this table reached nominal significance in the IVW analysis (IVW *p* < 0.05) for OP or MASLD. MR, Mendelian randomization; IVW, inverse variance-weighted; OR, odds ratio; CI, confidence interval; nSNP, number of instrumental variables; OP, osteoporosis; MASLD, metabolic dysfunction-associated steatotic liver disease. The MR-Egger intercept was used to assess directional horizontal pleiotropy. Heterogeneity *p*-values were calculated using Cochran’s Q test.

**Table 2:** Colocalization analysis for *MUC20* in OP and MASLD.

Gene	Outcome	nsnps	PP.H0	PP.H1	PP.H2	PP.H3	PP.H4
*MUC20*	OP	82	0.000	0.061	0.000	0.014	0.855
*MUC20*	MASLD	82	0.000	0.076	0.000	0.022	0.816

Note: PP.H0–PP.H4 represent posterior probabilities for five hypotheses: no association with either trait, association with *MUC20* expression only, association with disease only, association with both traits through distinct causal variants, and association with both traits through a shared causal variant, respectively.

**Table 3:** Baseline characteristics of participants in the exploratory clinical cohort.

Characteristic	Healthy Controls (*n* = 20)	OP+MASLD (*n* = 32)	*p*-Value
Age, years	53.00 ± 7.21	57.44 ± 12.21	0.106
BMI, kg/m^2^	23.15 ± 3.45	24.19 ± 3.13	0.282
Lumbar spine T-score	0.03 ± 0.56	−3.19 ± 0.42	<0.001
ALT, U/L	37.43 ± 13.77	56.16 ± 9.36	<0.001
AST, U/L	17.90 (15.75, 20.73)	42.05 (32.27, 49.80)	<0.001
TG, mmol/L	1.16 (0.80, 1.44)	3.59 (2.94, 4.13)	<0.001
TC, mmol/L	4.12 ± 0.66	6.26 ± 0.46	<0.001

Note: Data are presented as mean ± SD or median (IQR), as appropriate. *p*-values were calculated using the independent-samples *t* test or Mann–Whitney U test.

## Data Availability

The public single-cell transcriptomic datasets analyzed in this study are available from the Gene Expression Omnibus under accession numbers GSE147287 and GSE289173. Genetic association and eQTL data used for Mendelian randomization were obtained from publicly available FinnGen/OpenGWAS and eQTLGen resources.

## Supplementary Materials

The following supporting information can be downloaded at: https://www.mdpi.com/article/10.3390/ijms27125453/s1.

## Abbreviations

Abbreviation	Full term
MASLD	Metabolic dysfunction-associated steatotic liver disease
OP	Osteoporosis
GEO	Gene Expression Omnibus
GO	Gene Ontology
BP	Biological process
CC	Cellular component
MF	Molecular function
KEGG	Kyoto Encyclopedia of Genes and Genomes
MR	Mendelian randomization
GWAS	Genome-wide association study
eQTL	Expression quantitative trait locus
LD	Linkage disequilibrium
KO	Knockout
CTD	Comparative Toxicogenomics Database
miRDB	microRNA target prediction database
STROBE-MR	Strengthening the Reporting of Observational Studies in Epidemiology using Mendelian Randomization
MUC20	Mucin 20, cell surface associated
TREM2	Triggering receptor expressed on myeloid cells 2
HGF	Hepatocyte growth factor
MET	MET proto-oncogene, receptor tyrosine kinase
cGAS	Cyclic GMP-AMP synthase
STING	Stimulator of interferon genes
