# Supplementary material for: Integrative Single-Cell Transcriptomic, Mendelian Randomization and In Silico Perturbation Analyses Prioritize MUC20 as a Candidate Gene Associated with Osteoporosis and Metabolic Dysfunction-Associated Steatotic Liver Disease in the Liver–Bone Axis
Source: Int J Mol Sci. 2026 Jun 16;27(12):5453. doi: 10.3390/ijms27125453 (PMC13300151; doi:10.3390/ijms27125453)
Supplement: Supplementary file 1 [file ijms-27-05453-s001.zip › Supplementary Figure S1-2.pdf]

Supplementary Figure S1. Macrophage subcluster marker expression in OP and MASLD.

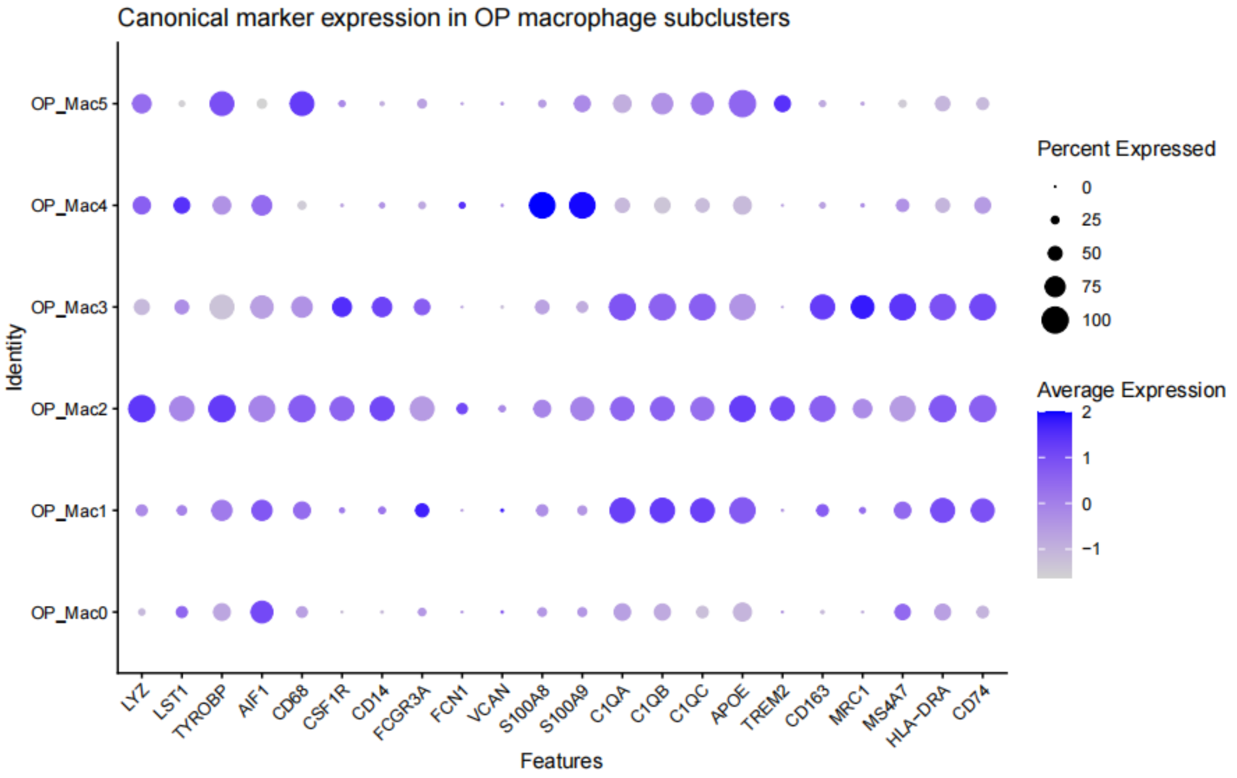

S1A. Marker expression in OP macrophage subclusters

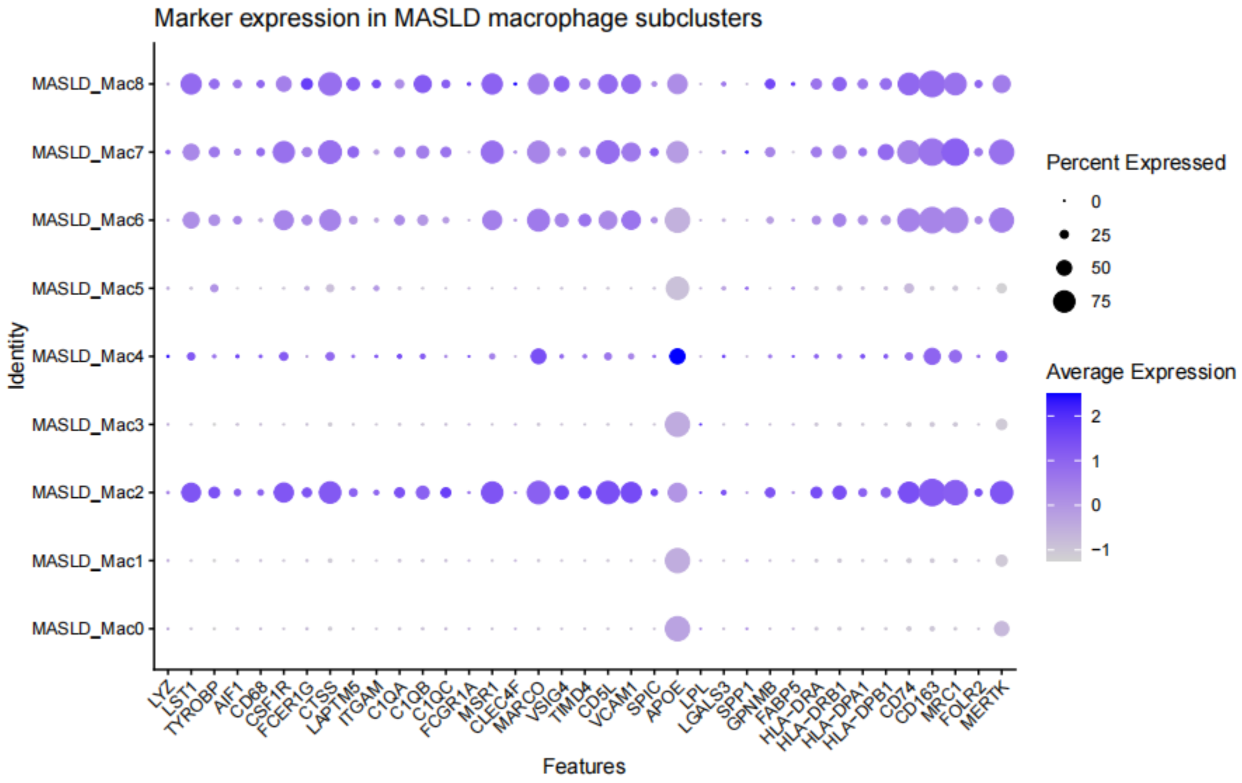

S1B. Marker expression in MASLD macrophage subclusters

Supplementary Figure S2. MUC20 SNP-level MR robustness analyses

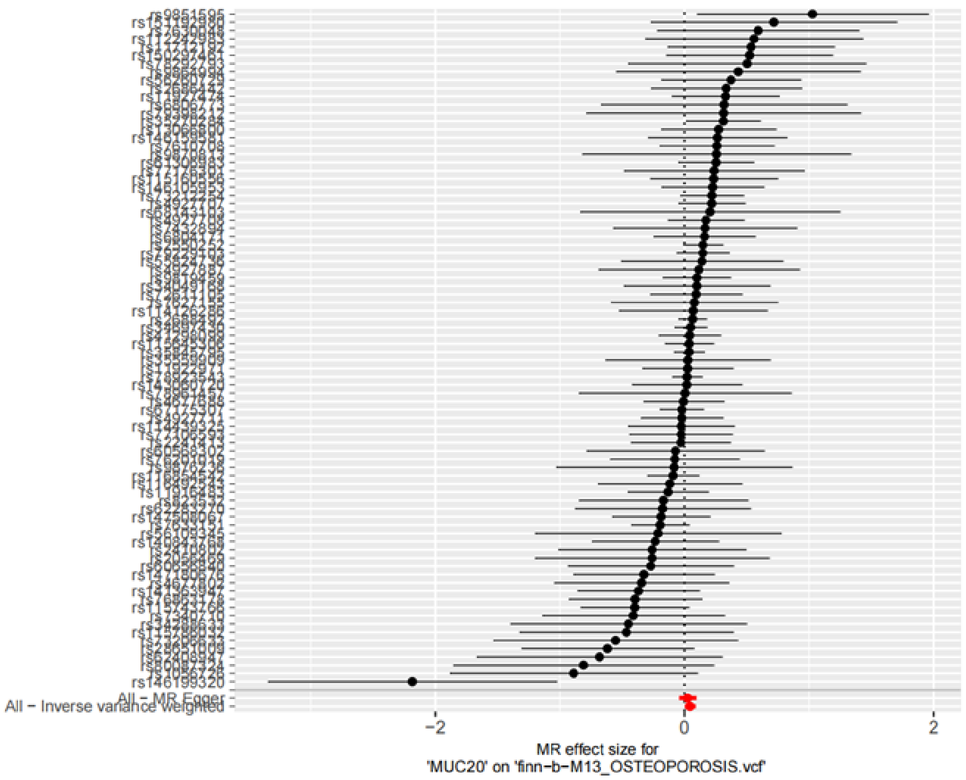

S2A. OP single-SNP MR estimates

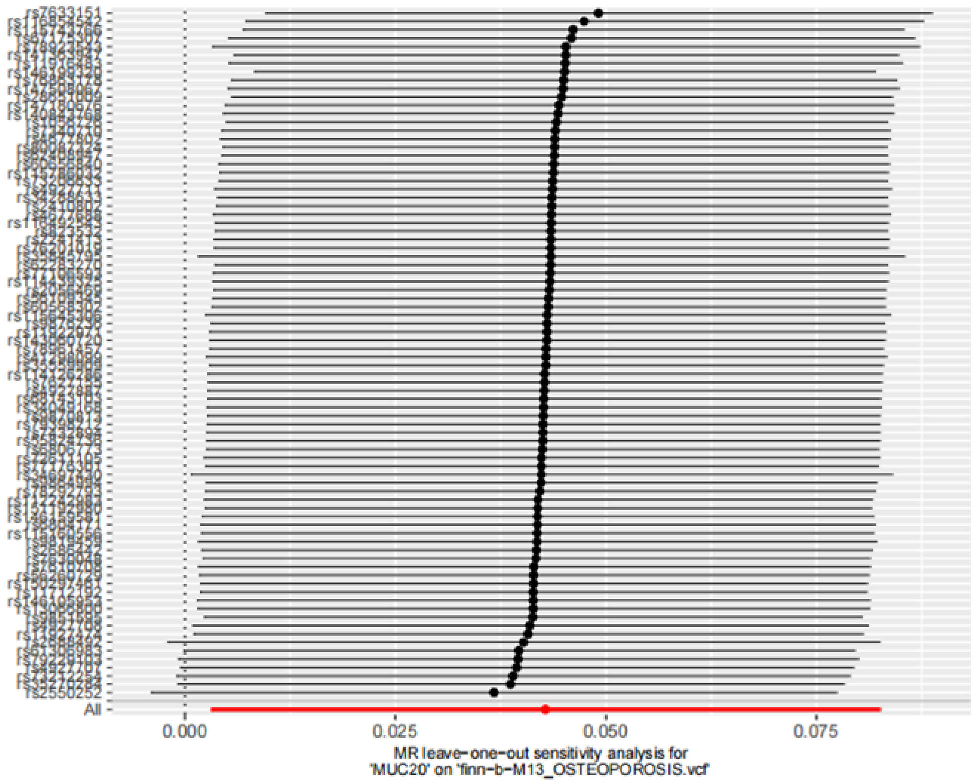

S2B. OP leave-one-out sensitivity analysis

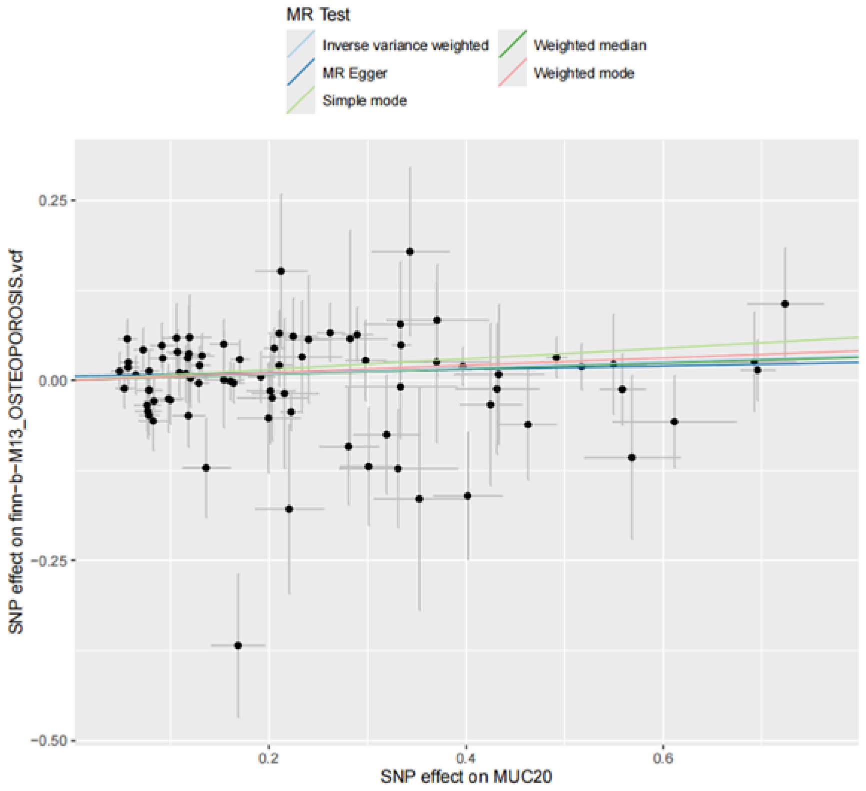

S2C. OP SNP-effect scatter plot

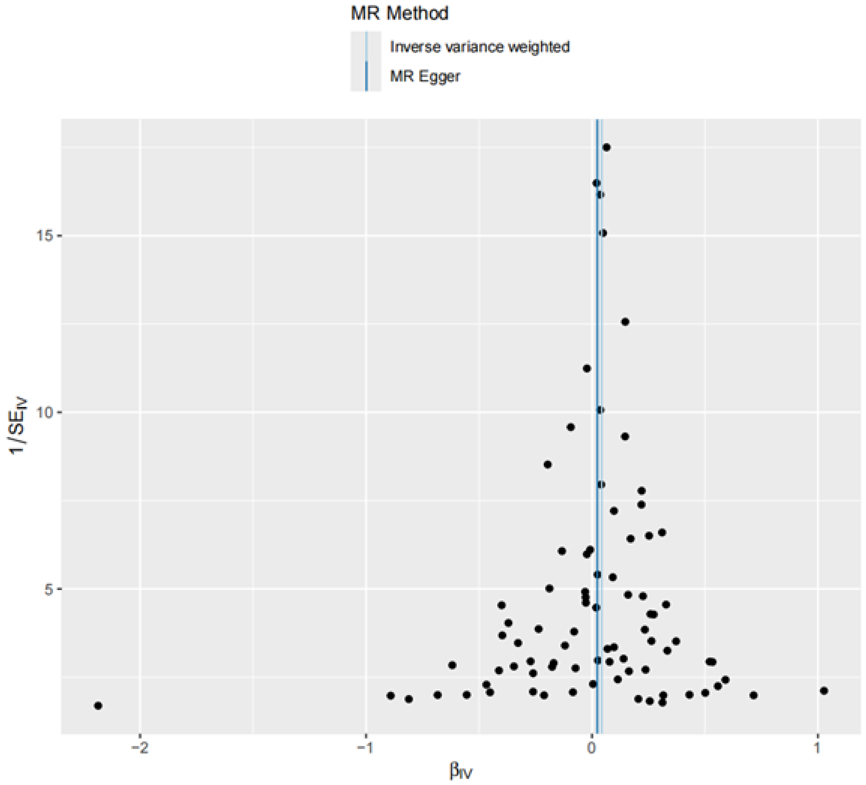

S2D. OP funnel plot

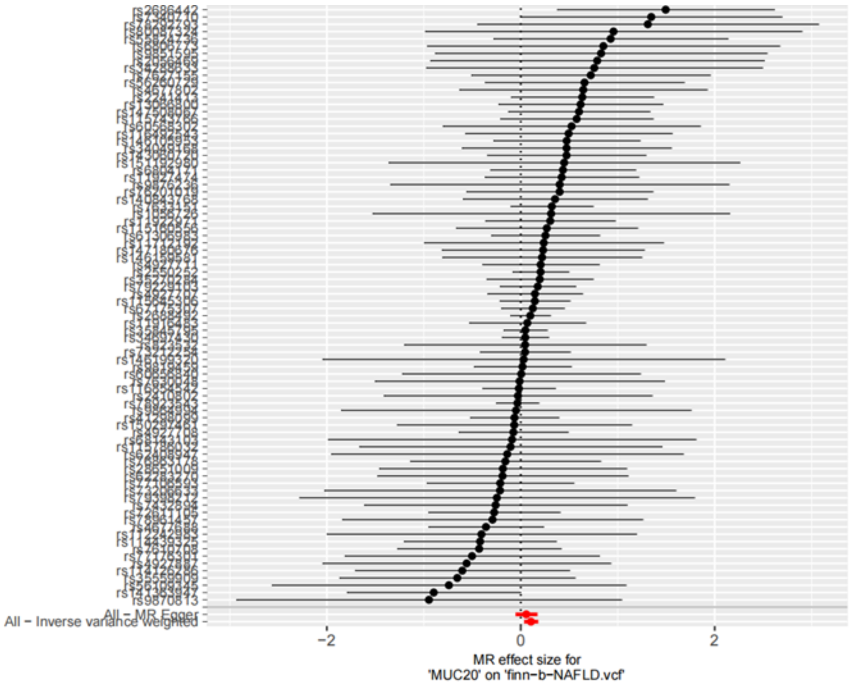

S2E. MASLD single-SNP MR estimates

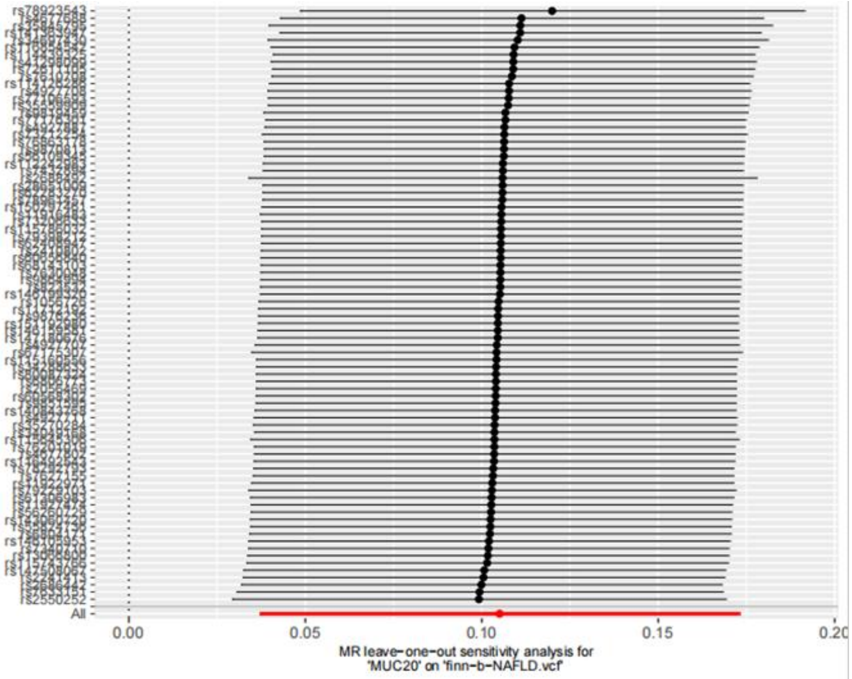

S2F. MASLD leave-one-out sensitivity analysis

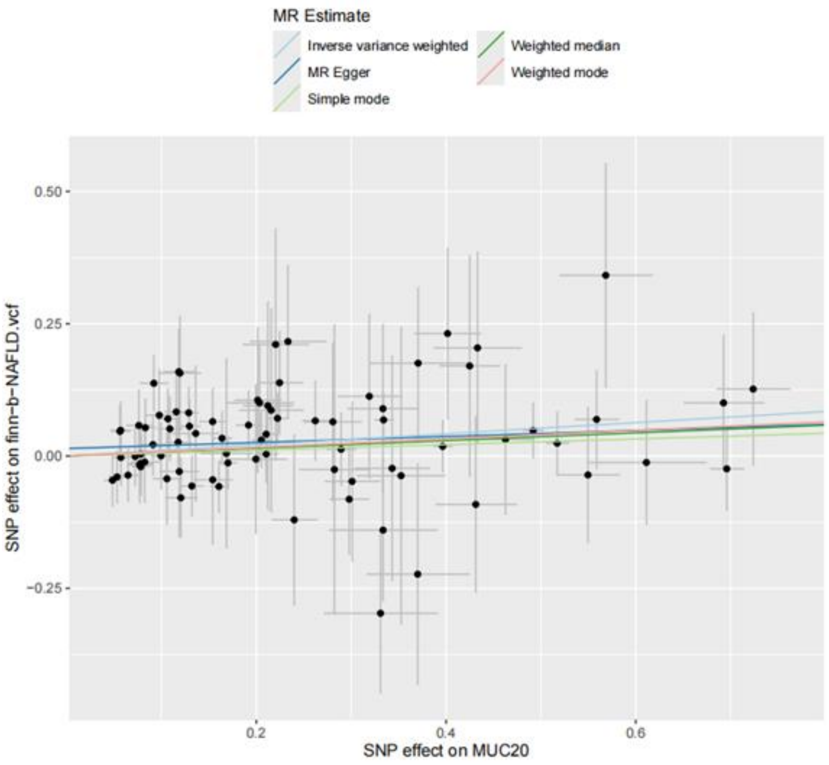

S2G. MASLD SNP-effect scatter plot

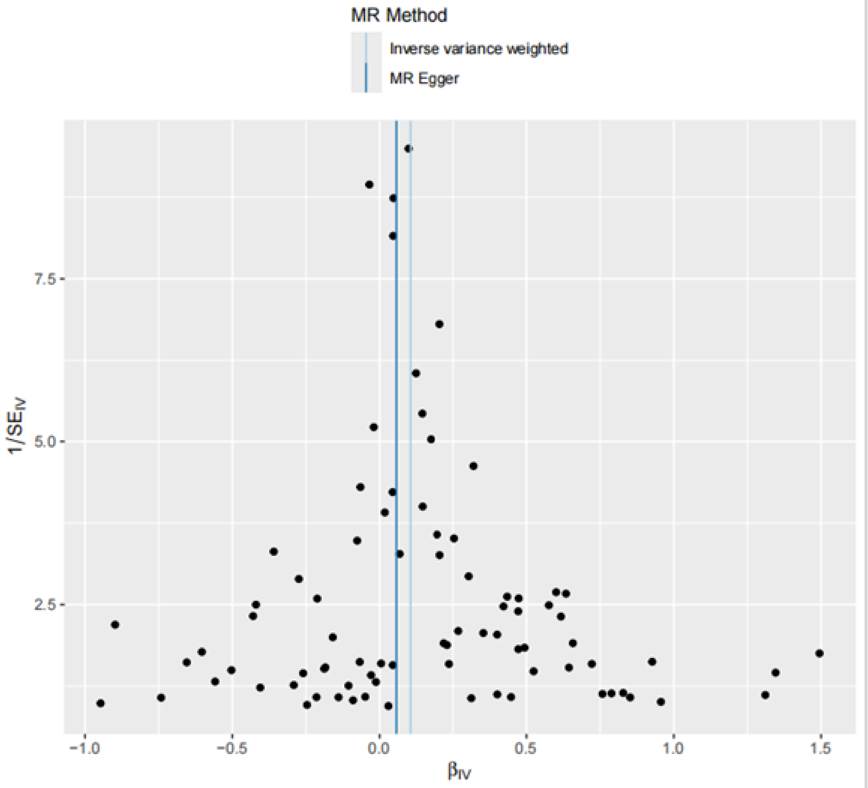

S2H. MASLD funnel plot
