# Supplementary material for: Integrative Single-Cell Transcriptomic, Mendelian Randomization and In Silico Perturbation Analyses Prioritize MUC20 as a Candidate Gene Associated with Osteoporosis and Metabolic Dysfunction-Associated Steatotic Liver Disease in the Liver–Bone Axis
Source: Int J Mol Sci. 2026 Jun 16;27(12):5453. doi: 10.3390/ijms27125453 (PMC13300151; doi:10.3390/ijms27125453)
Supplement: Supplementary file 1 [file ijms-27-05453-s001.zip › Supplementary Tables S1-4.pdf]

Supplementary Table S1. MR genes significantly associated with OP outcomes (IVW, p<0.05; n=10)

Gene	Outcome	nSNP	OR	CI_low	CI_high	P	FDR-adjusted P value	Direction
GGH	OP (finn-b-M13_OSTEOPOROSIS)	19	0.765	0.659	0.887	0.0004	0.0041	Protective (OR<1)
ARHGEF10	OP (finn-b-M13_OSTEOPOROSIS)	51	1.086	1.029	1.145	0.0026	0.0087	Risk (OR>1)
CD22	OP (finn-b-M13_OSTEOPOROSIS)	5	1.463	1.135	1.884	0.0033	0.0087	Risk (OR>1)
KCNE1	OP (finn-b-M13_OSTEOPOROSIS)	15	1.220	1.068	1.393	0.0035	0.0087	Risk (OR>1)
ANK3	OP (finn-b-M13_OSTEOPOROSIS)	54	0.922	0.870	0.977	0.0061	0.0102	Protective (OR<1)
TNK2	OP (finn-b-M13_OSTEOPOROSIS)	5	1.529	1.129	2.072	0.0061	0.0102	Risk (OR>1)
ACSM3	OP (finn-b-M13_OSTEOPOROSIS)	9	0.763	0.613	0.948	0.0149	0.0212	Protective (OR<1)
MUC20	OP (finn-b-M13_OSTEOPOROSIS)	82	1.044	1.003	1.086	0.0344	0.0401	Risk (OR>1)
LAP3	OP (finn-b-M13_OSTEOPOROSIS)	67	0.955	0.914	0.998	0.0384	0.0401	Protective (OR<1)
ECHDC3	OP (finn-b-M13_OSTEOPOROSIS)	34	0.929	0.867	0.997	0.0401	0.0401	Protective (OR<1)

Note: OR, odds ratio; CI, confidence interval; nSNP, number of instrumental variables; IVW, inverse-variance weighted; FDR, false discovery rate. FDR-adjusted P values were calculated using the Benjamini–Hochberg method based on the IVW P values. OR > 1 indicates a risk effect, and OR < 1 indicates a protective effect.

Supplementary Table S2. Instrumental Variable SNPs and Exposure/Outcome Effects of Genes Significantly Associated with OP Outcomes (Aligned by Allele; n=343 rows

Gene	SNP	EA_exposure	OA_exposure	Beta_exposure	SE_exposure	P_exposure	EAF_exposure	F_value	Beta_outcome_aligned	SE_outcome	P_outcome	EAF_outcome_aligned	Palindromic	Allele_alignment
ACSM3	rs2071524	A	G	-0.2248	0.0115	9.32e-85	0.138	65407871228.00	0.0115	0.0341	0.7346	0.178	No	Aligned
ACSM3	rs2245256	G	A	0.0957	0.0092	2.13e-25	0.249	4880000000.00	-0.0382	0.0304	0.2089	0.240	No	Flipped
ACSM3	rs3826157	G	A	0.0605	0.0091	2.96e-11	0.256	138000000000.00	-0.0432	0.0285	0.1295	0.294	No	Aligned
ACSM3	rs139518024	T	C	-0.2193	0.0332	4.08e-11	0.017	5724836003 7.00	0.1475	0.1153	0.2006	0.013	No	Aligned
ACSM3	rs111810458	C	T	-0.1782	0.0272	5.64e-11	0.026	86587950502.00	0.0770	0.1360	0.5715	0.010	No	Aligned
ACSM3	rs117608881	C	T	-0.2322	0.0363	1.61e-10	0.017	50668559225.00	0.1968	0.0899	0.0286	0.021	No	Aligned
ACSM3	rs72780826	T	C	-0.0539	0.0087	5.09e-10	0.699	9320000000.00	0.0242	0.0270	0.3709	0.362	No	Aligned
ACSM3	rs77964417	A	G	0.0738	0.0121	1.16e-09	0.124	9600000000.00	-0.0368	0.0364	0.3120	0.149	No	Aligned
ACSM3	rs143400234	C	T	-0.1480	0.0266	2.64e-08	0.031	1200000000.00	-0.0088	0.0877	0.9198	0.025	No	Aligned

ANK3	rs11817236	G	A	-0.3441	0.0080	0.00e+00	0.521	294537789501.00	0.0346	0.0261	0.1848	0.460	No	Aligned
ANK3	rs16915196	G	A	-0.4895	0.0116	0.00e+00	0.138	145475119204.00	0.0370	0.0373	0.3211	0.143	No	Aligned
ANK3	rs7905531	T	C	0.3138	0.0082	0.00e+00	0.376	391004660104.00	-0.0065	0.0273	0.8116	0.341	No	Aligned
ANK3	rs114768836	G	T	0.5508	0.0197	2.06e-172	0.044	129443027700.00	-0.0183	0.0815	0.8227	0.026	No	Aligned
ANK3	rs78581455	C	T	-0.6559	0.0239	2.47e-165	0.032	7901200084.00	-0.0739	0.0800	0.3556	0.028	No	Aligned
ANK3	rs10994296	G	A	-0.2812	0.0144	1.40e-84	0.083	41786312266.00	0.0298	0.0355	0.4003	0.160	No	Aligned
ANK3	rs57450815	C	T	-0.2196	0.0115	6.69e-82	0.143	68413328056.00	-0.0060	0.0363	0.8693	0.151	No	Aligned
ANK3	rs6479706	G	A	0.1547	0.0086	1.73e-72	0.314	17100000000.00	-0.0799	0.0280	0.0043	0.316	No	Flipped
ANK3	rs61856263	T	C	-0.2324	0.0131	4.34e-70	0.103	60586379510.00	0.0399	0.0359	0.2658	0.156	No	Aligned
ANK3	rs79867207	C	T	-0.3582	0.0216	5.52e-62	0.037	25326154405.00	0.0023	0.0800	0.9766	0.029	No	Aligned
ANK3	rs2279942	T	C	-0.1314	0.0088	1.28e-50	0.710	18600000000.00	0.0164	0.0282	0.5622	0.302	No	Aligned
ANK3	rs78839797	C	T	-0.1879	0.0128	4.81e-49	0.111	90709145177.00	0.0567	0.0402	0.1587	0.126	No	Aligned
ANK3	rs56068978	C	G	-0.1155	0.0085	2.71e-42	0.333	23800000000.00	-0.0018	0.0262	0.9454	0.420	Yes	Aligned
ANK3	rs12784166	G	A	-0.2797	0.0216	2.92e-38	0.038	40218573221.00	-0.0104	0.0567	0.8550	0.057	No	Aligned
ANK3	rs55683151	C	T	-0.4303	0.0342	2.60e-36	0.018	16922686267.00	0.0536	0.1493	0.7194	0.008	No	Aligned
ANK3	rs117026052	A	G	-0.8452	0.0679	1.46e-35	0.013	4379449003.00	0.1469	0.2911	0.6138	0.002	No	Aligned
ANK3	rs10821767	A	T	0.1513	0.0124	3.98e-34	0.883	18900000000.00	-0.0636	0.0418	0.1281	0.110	Yes	Aligned
ANK3	rs4542352	T	C	-0.0970	0.0082	1.42e-32	0.397	33000000000.00	-0.0008	0.0267	0.9755	0.387	No	Flipped
ANK3	rs10994281	G	A	-0.1193	0.0102	9.45e-32	0.203	21800000000.00	0.0421	0.0320	0.1887	0.214	No	Aligned
ANK3	rs141837303	A	G	0.2559	0.0223	2.23e-30	0.034	66945639912.00	-0.0642	0.0781	0.4113	0.029	No	Aligned
ANK3	rs55838521	G	A	0.1291	0.0116	7.29e-29	0.140	26400000000.00	-0.0128	0.0421	0.7609	0.107	No	Aligned
ANK3	rs7910802	A	G	-0.2415	0.0218	1.38e-28	0.038	52702513713.00	0.0793	0.0711	0.2644	0.035	No	Aligned
ANK3	rs3793860	A	G	-0.1909	0.0177	3.25e-27	0.054	83975770773.00	0.1025	0.0558	0.0664	0.057	No	Aligned
AN	rs32	C	T	0.120	0.01	4.37	0.146	30900	-0.0189	0.03	0.5	0.197	No	Align

K3	13025			1	14	e-26		0000000.00		25	620			ed
ANK3	rs7073658	T	G	-0.0839	0.0081	4.07e-25	0.414	43100000000.00	0.0065	0.0260	0.8037	0.486	No	Flipped
ANK3	rs79102984	T	C	0.1791	0.0177	3.62e-24	0.058	14000000000.00	0.0141	0.0669	0.8328	0.039	No	Aligned
ANK3	rs3213017	T	G	-0.2801	0.0280	1.63e-23	0.023	38457943751.00	0.0073	0.1676	0.9653	0.006	No	Aligned
ANK3	rs10994453	T	G	0.2052	0.0213	6.20e-22	0.039	10800000000.00	0.0560	0.0832	0.5009	0.025	No	Aligned
ANK3	rs72811877	G	A	0.1696	0.0189	2.50e-19	0.058	16100000000.00	-0.0646	0.0554	0.2436	0.058	No	Aligned
ANK3	rs80263212	A	T	-0.1747	0.0199	1.97e-18	0.045	96388204403.00	-0.0521	0.0573	0.3625	0.056	Yes	Aligned
ANK3	rs138052944	G	A	-0.3163	0.0374	2.95e-17	0.015	29189848598.00	-0.1794	0.1506	0.2336	0.008	No	Aligned
ANK3	rs41274674	A	G	-0.1779	0.0213	7.49e-17	0.039	91969226738.00	-0.0272	0.0588	0.6437	0.052	No	Aligned
ANK3	rs10740011	G	T	0.0658	0.0080	2.04e-16	0.454	109000000000.00	-0.0177	0.0265	0.5036	0.423	No	Flipped
ANK3	rs112686360	C	T	0.1784	0.0219	3.38e-16	0.037	14900000000.00	0.0625	0.0962	0.5159	0.019	No	Aligned
ANK3	rs79217840	A	G	-0.1548	0.0190	4.40e-16	0.051	12100000000.00	0.0953	0.0706	0.1768	0.036	No	Aligned
ANK3	rs149297290	C	T	-0.1797	0.0224	1.01e-15	0.034	89403693157.00	0.0403	0.0773	0.6018	0.029	No	Aligned
ANK3	rs6479688	G	A	-0.0651	0.0082	2.95e-15	0.376	67900000000.00	0.0064	0.0262	0.8060	0.444	No	Aligned
ANK3	rs75026145	T	C	-0.2312	0.0295	5.16e-15	0.024	53724340304.00	0.0880	0.1691	0.6030	0.007	No	Aligned
ANK3	rs140656002	C	T	-0.1912	0.0245	5.68e-15	0.031	78484163047.00	-0.0246	0.0820	0.7638	0.026	No	Aligned
ANK3	rs3213056	A	G	0.1518	0.0207	2.54e-13	0.046	21300000000.00	-0.0334	0.0714	0.6403	0.034	No	Aligned
ANK3	rs73261169	G	T	0.0778	0.0106	2.58e-13	0.177	80900000000.00	-0.0175	0.0374	0.6405	0.145	No	Aligned
ANK3	rs76667912	T	C	0.0771	0.0106	4.38e-13	0.826	82700000000.00	0.0094	0.0346	0.7860	0.170	No	Aligned
ANK3	rs10994141	C	A	-0.1979	0.0279	1.33e-12	0.031	71646773157.00	0.0869	0.0492	0.0773	0.075	No	Aligned
ANK3	rs75686970	T	C	-0.1831	0.0263	3.31e-12	0.026	83322189857.00	-0.0109	0.0854	0.8982	0.023	No	Aligned
ANK3	rs1183347	T	C	0.0614	0.0090	6.94e-12	0.274	133000000000.00	-0.0303	0.0317	0.3394	0.211	No	Flipped
ANK3	rs10994406	C	T	0.1849	0.0277	2.67e-11	0.024	14800000000.00	-0.0537	0.0836	0.5203	0.026	No	Aligned
ANK3	rs108216	C	T	0.0885	0.0134	4.34e-11	0.098	6480000000	0.0595	0.0553	0.2815	0.059	No	Aligned

	83							00.00						
ANK3	rs117908493	G	A	-0.1961	0.0306	1.42e-10	0.020	71057951755.00	0.0962	0.1156	0.4056	0.012	No	Aligned
ANK3	rs10821844	A	G	-0.0533	0.0084	2.30e-10	0.362	958000000000.00	0.0190	0.0268	0.4794	0.385	No	Aligned
ANK3	rs9415604	A	G	0.1288	0.0207	4.77e-10	0.042	312000000000.00	-0.0816	0.0658	0.2150	0.040	No	Aligned
ANK3	rs146953336	T	G	-0.3689	0.0602	8.88e-10	0.013	19837370875.00	-0.3022	0.2087	0.1476	0.004	No	Aligned
ANK3	rs117717606	A	G	-0.3373	0.0582	6.80e-09	0.013	23340268772.00	-0.3370	0.2038	0.0982	0.004	No	Aligned
ANK3	rs10994282	G	A	0.0659	0.0116	1.48e-08	0.145	1240000000000.00	-0.0076	0.0383	0.8419	0.135	No	Aligned
ANK3	rs76932066	A	G	0.0766	0.0137	2.10e-08	0.098	923000000000.00	-0.0105	0.0486	0.8288	0.080	No	Aligned
ARHGEF10	rs4875950	C	A	0.5478	0.0098	0.00e+00	0.715	126146062300.00	0.0412	0.0292	0.1580	0.271	No	Aligned
ARHGEF10	rs7003708	C	G	-0.3380	0.0096	2.26e-269	0.287	30210387425.00	-0.0596	0.0287	0.0380	0.283	Yes	Flipped
ARHGEF10	rs28643346	A	G	0.2569	0.0101	1.06e-141	0.248	59969949183.00	0.0251	0.0298	0.4000	0.261	No	Flipped
ARHGEF10	rs74415378	T	C	0.3120	0.0145	5.87e-103	0.102	41247199563.00	0.1067	0.0457	0.0194	0.088	No	Aligned
ARHGEF10	rs74304056	A	G	0.4357	0.0207	1.60e-98	0.952	21202877785.00	-0.0135	0.0543	0.8035	0.061	No	Aligned
ARHGEF10	rs117680534	T	C	-0.2731	0.0135	5.27e-91	0.142	44445142164.00	0.0301	0.0404	0.4554	0.122	No	Aligned
ARHGEF10	rs56040809	T	C	0.4935	0.0249	1.12e-87	0.035	16628823331.00	0.0494	0.0830	0.5519	0.026	No	Aligned
ARHGEF10	rs117522150	T	C	0.4725	0.0250	2.04e-79	0.037	18238582969.00	0.0042	0.1345	0.9753	0.010	No	Aligned
ARHGEF10	rs3735870	A	G	-0.1662	0.0089	5.99e-78	0.526	119000000000.00	-0.0190	0.0268	0.4790	0.597	No	Flipped
ARHGEF10	rs7013741	A	G	0.2273	0.0123	8.75e-77	0.148	78942188979.00	0.0819	0.0372	0.0279	0.147	No	Aligned
ARHGEF10	rs11784076	C	A	0.1658	0.0094	3.51e-69	0.653	149000000000.00	-0.0307	0.0265	0.2471	0.402	No	Aligned
ARHGEF10	rs62477479	T	C	0.1503	0.0088	6.56e-65	0.416	183000000000.00	-0.0176	0.0264	0.5049	0.406	No	Aligned

ARHGEF10	rs132711 33	T	C	-0.1903	0.0113	2.05e-63	0.184	8983430144 7.00	0.0080	0.0302	0.7926	0.241	No	Aligned
ARHGEF10	rs2272713	C	T	0.1635	0.0106	4.59e-54	0.783	1560000000 00.00	0.0746	0.0331	0.0242	0.191	No	Aligned
ARHGEF10	rs2280902	A	G	0.1342	0.0088	6.46e-53	0.443	2320000000 00.00	-0.0145	0.0264	0.5819	0.417	No	Flipped
ARHGEF10	rs2977194	G	A	-0.1072	0.0093	1.30e-30	0.329	2690000000 00.00	-0.0251	0.0271	0.3542	0.364	No	Flipped
ARHGEF10	rs2977197	G	C	0.1875	0.0167	3.39e-29	0.082	1250000000 00.00	0.0049	0.0488	0.9205	0.078	Yes	Flipped
ARHGEF10	rs118144264	A	C	-0.1684	0.0154	9.79e-28	0.088	1080000000 00.00	-0.0378	0.0428	0.3777	0.103	No	Aligned
ARHGEF10	rs3779697	G	A	0.2634	0.0248	2.41e-26	0.033	6414736736 8.00	0.0490	0.0548	0.3707	0.059	No	Aligned
ARHGEF10	rs11782618	C	A	0.1629	0.0154	3.47e-26	0.897	1680000000 00.00	-0.0372	0.0368	0.3117	0.153	No	Aligned
ARHGEF10	rs73180724	G	A	0.4913	0.0465	4.47e-26	0.015	1845567563 3.00	-0.0383	0.1704	0.8224	0.006	No	Aligned
ARHGEF10	rs141683526	G	A	-0.4241	0.0402	5.16e-26	0.020	1693711790 5.00	0.0503	0.1222	0.6807	0.012	No	Aligned
ARHGEF10	rs149730281	A	G	-0.3618	0.0368	7.94e-23	0.023	2298352036 6.00	-0.1903	0.1164	0.1022	0.014	No	Aligned
ARHGEF10	rs10503169	G	A	0.1860	0.0197	2.98e-21	0.065	1320000000 00.00	0.1113	0.0541	0.0399	0.062	No	Aligned
ARHGEF10	rs2977180	T	A	-0.0844	0.0091	2.24e-20	0.589	4170000000 00.00	-0.0148	0.0268	0.5802	0.396	Yes	Flipped
ARHGEF10	rs111801715	G	C	-0.1352	0.0149	1.14e-19	0.101	1620000000 00.00	-0.0681	0.0443	0.1245	0.093	Yes	Aligned
ARHGEF10	rs117416957	A	G	-0.4231	0.0475	5.10e-19	0.017	1648699897 9.00	0.1267	0.0749	0.0907	0.033	No	Aligned
ARHGEF10	rs112158571	T	G	-0.2346	0.0267	1.62e-18	0.035	5347752637 5.00	0.0152	0.0677	0.8229	0.038	No	Aligned
ARHGEF10	rs118005533	A	G	-0.2246	0.0260	5.57e-18	0.035	5816544868 8.00	-0.1499	0.0952	0.1155	0.020	No	Aligned
ARHG	rs142314	T	C	0.3107	0.0367	2.74e-17	0.017	4866318734	0.0113	0.0921	0.9020	0.020	No	Aligned

EF10	372							9.00						
ARHGEF10	rs111723745	T	G	0.2867	0.0398	5.98e-13	0.016	59898361026.00	-0.0352	0.1167	0.7628	0.013	No	Aligned
ARHGEF10	rs150711293	T	C	-0.2584	0.0362	1.01e-12	0.018	42059151555.00	-0.0645	0.0604	0.2856	0.048	No	Aligned
ARHGEF10	rs13279485	C	T	0.1242	0.0174	1.05e-12	0.080	32000000000.00	0.0066	0.0521	0.8989	0.071	No	Aligned
ARHGEF10	rs147583915	T	C	0.1368	0.0193	1.42e-12	0.055	26500000000.00	-0.0194	0.0619	0.7537	0.048	No	Aligned
ARHGEF10	rs111994531	G	A	-0.2032	0.0297	7.56e-12	0.028	67312077436.00	0.0036	0.0556	0.9487	0.059	No	Aligned
ARHGEF10	rs144144577	C	T	0.3455	0.0507	9.31e-12	0.014	42017833086.00	0.0584	0.1044	0.5763	0.016	No	Aligned
ARHGEF10	rs13279665	C	A	0.0900	0.0133	1.19e-11	0.142	62000000000.00	0.0190	0.0337	0.5728	0.192	No	Aligned
ARHGEF10	rs35266351	G	C	0.1180	0.0186	2.25e-10	0.058	36900000000.00	-0.0408	0.0620	0.5098	0.046	Yes	Aligned
ARHGEF10	rs62477527	C	T	-0.0847	0.0137	6.81e-10	0.115	37700000000.00	0.0053	0.0394	0.8931	0.130	No	Aligned
ARHGEF10	rs7835707	C	A	-0.0622	0.0102	9.77e-10	0.754	69700000000.00	-0.0253	0.0280	0.3671	0.313	No	Aligned
ARHGEF10	rs17749739	T	G	-0.1408	0.0233	1.49e-09	0.047	13600000000.00	0.0373	0.0576	0.5175	0.057	No	Aligned
ARHGEF10	rs7013586	T	C	-0.1478	0.0247	2.26e-09	0.033	12300000000.00	-0.1876	0.1447	0.1947	0.008	No	Aligned
ARHGEF10	rs7001454	G	A	0.0917	0.0154	2.52e-09	0.089	62600000000.00	0.0906	0.0411	0.0275	0.115	No	Aligned
ARHGEF10	rs142696840	A	G	-0.1881	0.0319	3.59e-09	0.023	75460404420.00	0.1785	0.1042	0.0867	0.016	No	Aligned
ARHGEF10	rs73672956	C	G	-0.1080	0.0186	6.28e-09	0.063	22800000000.00	-0.0437	0.0506	0.3879	0.071	Yes	Aligned
ARHGEF10	rs3758018	T	C	0.0575	0.0100	7.77e-09	0.585	1610000000000.00	0.0142	0.0267	0.5946	0.431	No	Aligned
ARHGEF10	rs7461958	T	C	-0.0520	0.0090	8.41e-09	0.368	98000000000.00	-0.0025	0.0267	0.9256	0.383	No	Aligned

ARHGEF10	rs9657384	G	T	-0.0587	0.0103	1.11e-08	0.258	768000000000.00	-0.0061	0.0298	0.8377	0.254	No	Flipped
ARHGEF10	rs7835584	A	T	0.1367	0.0242	1.57e-08	0.045	289000000000.00	0.0165	0.0647	0.7982	0.046	Yes	Aligned
ARHGEF10	rs1006520	G	A	0.0590	0.0107	3.58e-08	0.245	1570000000000.00	0.0068	0.0289	0.8138	0.295	No	Aligned
ARHGEF10	rs138632090	T	C	-0.3325	0.0607	4.39e-08	0.014	23619720301.00	0.0447	0.1152	0.6982	0.014	No	Aligned
CD22	rs12327820	C	A	0.2222	0.0136	7.33e-60	0.114	83926821750.00	0.0705	0.0399	0.0768	0.122	No	Aligned
CD22	rs11666282	T	C	-0.0962	0.0104	1.51e-20	0.773	322000000000.00	-0.0600	0.0317	0.0584	0.213	No	Aligned
CD22	rs10422744	T	C	0.0801	0.0099	4.14e-16	0.390	739000000000.00	0.0407	0.0266	0.1262	0.408	No	Aligned
CD22	rs1978014	G	A	0.0699	0.0089	4.97e-15	0.417	984000000000.00	0.0191	0.0264	0.4688	0.432	No	Flipped
CD22	rs4805119	G	A	-0.0523	0.0094	2.72e-08	0.688	958000000000.00	-0.0095	0.0278	0.7341	0.328	No	Aligned
ECHDC3	rs718641	C	T	-0.4659	0.0086	0.00e+00	0.683	16224082228.00	0.0221	0.0283	0.4333	0.300	No	Flipped
ECHDC3	rs11257331	A	G	0.1960	0.0086	1.56e-114	0.319	104000000000.00	-0.0016	0.0280	0.9531	0.318	No	Aligned
ECHDC3	rs11257300	G	A	0.3147	0.0143	1.54e-107	0.086	40474757610.00	-0.0710	0.0447	0.1121	0.092	No	Aligned
ECHDC3	rs7477429	G	A	-0.3619	0.0172	8.73e-98	0.059	25405085117.00	0.0455	0.0604	0.4506	0.049	No	Aligned
ECHDC3	rs4747928	T	C	0.1593	0.0090	1.77e-70	0.270	162000000000.00	-0.0334	0.0288	0.2468	0.292	No	Aligned
ECHDC3	rs113165185	A	G	-0.4847	0.0283	8.36e-66	0.024	13877935439.00	-0.0119	0.0812	0.8836	0.027	No	Aligned
ECHDC3	rs60806452	A	G	-0.3474	0.0214	2.40e-59	0.039	26845423559.00	0.0506	0.0563	0.3696	0.056	No	Aligned
ECHDC3	rs76046976	T	G	-0.4615	0.0317	6.08e-48	0.020	15007661053.00	0.0444	0.0840	0.5968	0.025	No	Aligned
ECHDC3	rs118165782	A	G	-0.2752	0.0193	3.63e-46	0.045	42090955792.00	0.0422	0.0600	0.4819	0.050	No	Aligned
ECHDC3	rs36066784	A	G	-0.1619	0.0114	3.80e-46	0.145	122000000000.00	0.0080	0.0326	0.8060	0.197	No	Aligned
ECHDC3	rs11257236	G	A	0.1077	0.0083	8.37e-39	0.372	370000000000.00	0.0030	0.0270	0.9114	0.360	No	Aligned
ECHDC3	rs17150721	G	C	-0.1136	0.0089	1.10e-37	0.284	243000000000.00	0.0106	0.0277	0.7028	0.332	Yes	Aligned
ECHDC3	rs79044909	T	C	0.2783	0.0220	1.24e-36	0.034	55599439053.00	0.0290	0.0640	0.6507	0.043	No	Aligned

ECHDC3	rs4747939	G	T	-0.5174	0.0424	2.84e-34	0.014	11653524643.00	-0.0269	0.1070	0.8016	0.015	No	Aligned
ECHDC3	rs139899818	A	G	0.2417	0.0222	1.74e-27	0.035	75847724542.00	0.0596	0.0971	0.5393	0.019	No	Aligned
ECHDC3	rs2399726	T	C	-0.0794	0.0087	5.39e-20	0.365	471000000000.00	0.0240	0.0277	0.3866	0.338	No	Aligned
ECHDC3	rs61844929	A	C	0.1411	0.0157	2.57e-19	0.072	232000000000.00	0.1203	0.0534	0.0242	0.064	No	Aligned
ECHDC3	rs117927419	T	C	-0.2360	0.0270	2.27e-18	0.026	52803429890.00	0.0580	0.0778	0.4560	0.029	No	Aligned
ECHDC3	rs240068	G	A	0.0702	0.0082	9.19e-18	0.399	948000000000.00	0.0072	0.0265	0.7846	0.426	No	Aligned
ECHDC3	rs35283504	T	C	-0.3258	0.0382	1.49e-17	0.014	27566790619.00	-0.0358	0.1144	0.7545	0.013	No	Aligned
ECHDC3	rs138462460	C	T	-0.3993	0.0469	1.61e-17	0.012	18343017401.00	0.0340	0.1095	0.7560	0.015	No	Aligned
ECHDC3	rs72779871	A	G	0.2834	0.0340	7.49e-17	0.016	58709112974.00	-0.1049	0.1002	0.2952	0.018	No	Aligned
ECHDC3	rs112559601	C	T	0.1890	0.0227	7.96e-17	0.036	132000000000.00	0.0453	0.0912	0.6194	0.022	No	Aligned
ECHDC3	rs41311226	C	T	-0.5959	0.0735	4.92e-16	0.014	8149542935.00	0.0059	0.1835	0.9745	0.005	No	Aligned
ECHDC3	rs7083962	A	G	-0.0672	0.0084	9.45e-16	0.352	639000000000.00	0.0184	0.0276	0.5053	0.328	No	Flipped
ECHDC3	rs7087572	A	G	0.0868	0.0121	7.90e-13	0.124	655000000000.00	-0.0510	0.0392	0.1933	0.126	No	Flipped
ECHDC3	rs12416487	A	T	-0.0558	0.0083	2.20e-11	0.640	889000000000.00	-0.0253	0.0268	0.3459	0.380	Yes	Flipped
ECHDC3	rs11524817	C	T	-0.0581	0.0088	4.70e-11	0.707	816000000000.00	0.0128	0.0286	0.6560	0.295	No	Flipped
ECHDC3	rs12779264	C	T	0.0894	0.0147	1.09e-09	0.094	654000000000.00	-0.0540	0.0397	0.1745	0.122	No	Aligned
ECHDC3	rs147520972	G	A	-0.1628	0.0278	4.57e-09	0.025	101000000000.00	0.1696	0.1358	0.2118	0.010	No	Aligned
ECHDC3	rs76011191	T	C	-0.2070	0.0356	6.22e-09	0.016	62004752813.00	0.1151	0.1098	0.2946	0.015	No	Aligned
ECHDC3	rs117238864	C	T	0.1139	0.0197	7.29e-09	0.050	411000000000.00	0.0337	0.0515	0.5127	0.070	No	Aligned
ECHDC3	rs115725431	C	T	0.1798	0.0313	9.21e-09	0.019	166000000000.00	-0.0463	0.0976	0.6348	0.019	No	Aligned
ECHDC3	rs142823982	C	T	0.1525	0.0266	9.94e-09	0.025	230000000000.00	-0.0013	0.0664	0.9847	0.039	No	Aligned
GGH	rs615183338	A	C	0.2974	0.0134	5.12e-109	0.098	45286747692.00	-0.1237	0.0541	0.0223	0.061	No	Aligned
GGH	rs75931208	A	G	0.5550	0.0348	3.87e-57	0.015	13494179567.00	-0.2134	0.1243	0.0860	0.012	No	Aligned
GG	rs74	A	G	-	0.01	6.72	0.928	57097	0.0368	0.04	0.4	0.095	No	Align

H	329104			0.2374	55	e-53		781062.00		41	038			ed
GGH	rs7006471	G	A	-0.1215	0.0080	1.94e-52	0.495	2180000000.00	0.0050	0.0265	0.8494	0.599	No	Aligned
GGH	rs72621442	G	A	-0.1323	0.0124	1.43e-26	0.124	1740000000.00	0.0124	0.0365	0.7338	0.151	No	Aligned
GGH	rs149997066	C	A	-0.2616	0.0338	1.03e-14	0.016	41846272662.00	0.1634	0.0874	0.0614	0.022	No	Aligned
GGH	rs143675371	T	C	0.2169	0.0287	4.04e-14	0.022	1030000000.00	0.0075	0.0683	0.9131	0.037	No	Aligned
GGH	rs4738991	A	G	0.0708	0.0098	4.65e-13	0.790	9820000000.00	-0.0047	0.0307	0.8781	0.234	No	Aligned
GGH	rs13261534	A	G	0.0978	0.0138	1.38e-12	0.097	5180000000.00	-0.0668	0.0560	0.2331	0.057	No	Aligned
GGH	rs79701164	T	C	-0.1113	0.0161	4.19e-12	0.069	2250000000.00	0.0317	0.0442	0.4735	0.097	No	Aligned
GGH	rs28651093	C	T	-0.1731	0.0269	1.20e-10	0.025	91350429060.00	0.0521	0.1240	0.6744	0.011	No	Aligned
GGH	rs139769108	C	T	0.2068	0.0327	2.50e-10	0.020	1200000000.00	0.0111	0.1237	0.9283	0.012	No	Aligned
GGH	rs58814013	A	G	0.0848	0.0135	3.80e-10	0.096	7190000000.00	-0.0853	0.0815	0.2953	0.027	No	Aligned
GGH	rs112906173	C	T	0.1783	0.0294	1.40e-09	0.021	1650000000.00	-0.1677	0.0757	0.0267	0.031	No	Aligned
GGH	rs117228301	C	T	-0.1372	0.0232	3.12e-09	0.033	1420000000.00	0.1179	0.0885	0.1831	0.022	No	Aligned
GGH	rs149838736	G	A	0.1910	0.0325	4.12e-09	0.018	1450000000.00	0.0764	0.1307	0.5592	0.010	No	Aligned
GGH	rs147477407	G	A	-0.0826	0.0144	8.63e-09	0.086	3880000000.00	0.0051	0.0409	0.9000	0.112	No	Aligned
GGH	rs117411647	T	C	0.1837	0.0323	1.27e-08	0.020	1590000000.00	-0.0575	0.0863	0.5053	0.023	No	Aligned
GGH	rs12056842	A	G	0.0662	0.0117	1.62e-08	0.134	1230000000000.00	-0.0213	0.0487	0.6616	0.077	No	Aligned
KCNE1	rs13050900	T	C	-0.3014	0.0087	5.05e-265	0.694	37992478360.00	-0.0375	0.0282	0.1844	0.304	No	Aligned
KCNE1	rs79538573	G	A	0.1698	0.0147	6.62e-31	0.080	1520000000.00	0.0782	0.0484	0.1060	0.077	No	Aligned
KCNE1	rs2284599	A	G	0.0954	0.0088	1.31e-27	0.293	4870000000.00	0.0274	0.0300	0.3605	0.251	No	Aligned
KCNE1	rs116851524	C	T	-0.1827	0.0173	3.62e-26	0.058	91342041871.00	-0.0822	0.0609	0.1769	0.048	No	Aligned
KCNE1	rs149179329	T	C	-0.3583	0.0372	5.38e-22	0.015	23350039618.00	0.0477	0.1160	0.6810	0.013	No	Aligned
KCNE1	rs41313001	A	G	-0.2470	0.0269	4.15e-20	0.025	48667282352.00	-0.1202	0.0653	0.0658	0.042	No	Aligned
KCNE1	rs765610	T	C	-0.066	0.0080	9.18e-17	0.552	6550000000	-0.0338	0.0267	0.2068	0.381	No	Aligned

				7				00.00						
KCNE1	rs2834609	A	G	0.0696	0.0085	1.75e-16	0.334	975000000000.00	0.0069	0.0274	0.8005	0.344	No	Aligned
KCNE1	rs2006998	G	A	0.1147	0.0141	4.46e-16	0.088	361000000000.00	0.0194	0.0394	0.6227	0.124	No	Aligned
KCNE1	rs7282586	A	G	-0.0954	0.0124	1.39e-14	0.124	314000000000.00	-0.0886	0.0381	0.0199	0.139	No	Flipped
KCNE1	rs118041668	T	C	-0.1119	0.0166	1.38e-11	0.069	221000000000.00	0.0432	0.0553	0.4345	0.059	No	Aligned
KCNE1	rs8129272	A	G	0.0786	0.0123	1.68e-10	0.119	830000000000.00	0.0092	0.0388	0.8123	0.129	No	Aligned
KCNE1	rs12626835	T	A	-0.0610	0.0105	7.00e-09	0.363	714000000000.00	-0.0203	0.0265	0.4436	0.410	Yes	Aligned
KCNE1	rs1010667	T	A	0.0733	0.0127	7.29e-09	0.112	993000000000.00	0.0017	0.0426	0.9683	0.105	Yes	Flipped
KCNE1	rs8129632	G	A	-0.0486	0.0089	4.00e-08	0.337	1110000000000.00	0.0194	0.0270	0.4730	0.395	No	Flipped
LAP3	rs10939731	T	C	0.6377	0.0092	0.00e+00	0.313	9245662416.00	-0.0438	0.0295	0.1385	0.266	No	Flipped
LAP3	rs13110646	G	T	0.3903	0.0103	0.00e+00	0.219	25291092276.00	-0.0316	0.0383	0.4093	0.134	No	Aligned
LAP3	rs28553924	A	G	-0.3736	0.0098	0.00e+00	0.236	24840407121.00	0.0074	0.0294	0.8023	0.268	No	Aligned
LAP3	rs41268399	A	G	0.6554	0.0208	8.31e-218	0.047	9066931643.00	-0.0755	0.0717	0.2925	0.034	No	Aligned
LAP3	rs113897883	T	C	0.6532	0.0210	4.52e-212	0.046	9137177210.00	-0.0855	0.0552	0.1216	0.060	No	Aligned
LAP3	rs41268395	A	G	0.6638	0.0240	1.35e-168	0.034	8921266518.00	-0.0577	0.0507	0.2557	0.070	No	Aligned
LAP3	rs6856261	T	C	-0.2567	0.0103	3.09e-138	0.215	51233004222.00	0.0107	0.0302	0.7238	0.248	No	Aligned
LAP3	rs2535224	C	T	-0.1720	0.0083	1.92e-94	0.522	112000000000.00	-0.0083	0.0259	0.7473	0.493	No	Aligned
LAP3	rs2658752	C	A	0.4554	0.0228	7.03e-89	0.038	19515228873.00	0.0301	0.0808	0.7098	0.028	No	Aligned
LAP3	rs77726502	T	C	-0.4012	0.0206	4.49e-84	0.048	20523893957.00	0.0704	0.0750	0.3480	0.032	No	Aligned
LAP3	rs76160459	T	C	-0.3463	0.0181	1.05e-81	0.061	27506273692.00	-0.0261	0.0520	0.6153	0.068	No	Aligned
LAP3	rs71603374	A	G	0.6908	0.0386	1.56e-71	0.015	8584577628.00	0.2190	0.1295	0.0908	0.011	No	Aligned
LAP3	rs17526078	T	C	0.3466	0.0198	1.15e-68	0.050	34187964844.00	-0.0198	0.0989	0.8413	0.018	No	Aligned
LAP3	rs2610989	T	C	-0.1609	0.0094	2.69e-65	0.292	126000000000.00	0.0301	0.0277	0.2771	0.324	No	Flipped
LAP3	rs41268403	T	G	0.5615	0.0340	3.04e-61	0.017	13124013832.00	-0.0707	0.1086	0.5149	0.014	No	Aligned

LAP3	rs115189991	G	A	-0.3522	0.0221	2.94e-57	0.042	26062772503.00	-0.0683	0.0509	0.1798	0.070	No	Aligned
LAP3	rs77506232	G	T	0.4015	0.0272	2.56e-49	0.026	26069978719.00	-0.0355	0.0681	0.6020	0.038	No	Aligned
LAP3	rs189101031	T	C	-0.4683	0.0319	1.22e-48	0.022	14594178512.00	0.0734	0.0685	0.2837	0.038	No	Aligned
LAP3	rs76625886	A	C	-0.3616	0.0251	5.55e-47	0.032	24410537611.00	-0.0675	0.0640	0.2917	0.043	No	Aligned
LAP3	rs79365683	C	T	0.3731	0.0274	2.88e-42	0.028	30558528251.00	0.0659	0.1304	0.6131	0.010	No	Aligned
LAP3	rs75282622	T	C	0.5538	0.0416	2.24e-40	0.014	13919565914.00	-0.0323	0.1821	0.8592	0.005	No	Aligned
LAP3	rs11735144	G	T	-0.2084	0.0161	4.17e-38	0.072	72436897544.00	-0.0238	0.0425	0.5763	0.106	No	Aligned
LAP3	rs7684006	A	G	0.1079	0.0085	4.87e-37	0.400	369000000000.00	-0.0066	0.0275	0.8106	0.337	No	Aligned
LAP3	rs12647355	C	T	0.1042	0.0086	1.86e-33	0.378	400000000000.00	0.0495	0.0269	0.0660	0.369	No	Aligned
LAP3	rs138961994	T	C	-0.3517	0.0299	6.14e-32	0.021	25074394451.00	0.0462	0.1367	0.7357	0.009	No	Aligned
LAP3	rs17521310	T	G	-0.2265	0.0194	1.98e-31	0.052	60359004628.00	0.0321	0.0565	0.5698	0.057	No	Aligned
LAP3	rs7663121	C	T	-0.1190	0.0103	8.01e-31	0.215	218000000000.00	-0.0528	0.0293	0.0722	0.273	No	Aligned
LAP3	rs148858957	C	A	-0.1733	0.0162	9.38e-27	0.080	102000000000.00	-0.0459	0.0431	0.2865	0.102	No	Aligned
LAP3	rs78261017	T	C	-0.2126	0.0202	6.87e-26	0.046	67346455909.00	0.0521	0.0647	0.4206	0.043	No	Aligned
LAP3	rs76421231	T	C	0.2136	0.0207	4.71e-25	0.048	98095436305.00	-0.1243	0.0757	0.1006	0.033	No	Aligned
LAP3	rs1901169	G	A	0.1090	0.0106	1.01e-24	0.196	377000000000.00	-0.0181	0.0312	0.5615	0.226	No	Aligned
LAP3	rs150530659	C	T	-0.3543	0.0352	7.64e-24	0.017	24069676363.00	-0.0012	0.1163	0.9920	0.013	No	Aligned
LAP3	rs75218392	G	A	-0.1720	0.0177	2.39e-22	0.060	102000000000.00	-0.0357	0.0553	0.5185	0.059	No	Aligned
LAP3	rs57499163	G	A	0.3425	0.0355	5.16e-22	0.017	38750289034.00	-0.0767	0.0968	0.4277	0.018	No	Aligned
LAP3	rs79792134	A	G	-0.1197	0.0129	1.55e-20	0.882	208000000000.00	0.0319	0.0374	0.3939	0.141	No	Aligned
LAP3	rs2597778	C	T	0.0820	0.0091	1.39e-19	0.698	687000000000.00	-0.0222	0.0313	0.4790	0.222	No	Flipped
LAP3	rs2518632	G	C	-0.1708	0.0194	1.09e-18	0.049	101000000000.00	0.0243	0.0491	0.6199	0.076	Yes	Flipped
LAP3	rs62410180	C	T	0.3660	0.0418	2.04e-18	0.014	34747007696.00	0.2174	0.1806	0.2286	0.005	No	Aligned
LAP	rs28	A	G	-	0.00	2.01	0.349	51800	0.0225	0.02	0.4	0.361	No	Flippe

3	60033			0.0751	88	e-17		0000000.00		72	071			d
LAP3	rs114517053	T	C	-0.2907	0.0351	1.09e-16	0.021	34409411090.00	0.0764	0.0859	0.3743	0.025	No	Aligned
LAP3	rs115808526	A	G	0.2969	0.0367	6.59e-16	0.016	53972714555.00	0.1579	0.1315	0.2298	0.010	No	Aligned
LAP3	rs73800224	A	G	-0.2507	0.0315	1.57e-15	0.019	45862010379.00	-0.1070	0.0920	0.2448	0.021	No	Aligned
LAP3	rs79720570	G	A	0.6059	0.0765	2.29e-15	0.017	13028975973.00	-0.7160	0.4398	0.1035	0.001	No	Aligned
LAP3	rs115188491	C	T	-0.2723	0.0353	1.33e-14	0.019	38592738934.00	0.0187	0.0864	0.8286	0.024	No	Aligned
LAP3	rs139263177	C	T	-0.2952	0.0389	3.02e-14	0.015	32724467098.00	0.0951	0.1159	0.4115	0.013	No	Aligned
LAP3	rs73230782	T	C	0.0928	0.0123	4.10e-14	0.868	563000000000.00	-0.0861	0.0430	0.0452	0.101	No	Aligned
LAP3	rs7690643	C	T	-0.0732	0.0099	1.37e-13	0.235	530000000000.00	-0.0246	0.0285	0.3885	0.297	No	Flipped
LAP3	rs147391655	G	T	0.3694	0.0545	1.16e-11	0.012	36802775396.00	-0.2724	0.2018	0.1770	0.005	No	Aligned
LAP3	rs77137476	G	A	-0.1781	0.0270	4.25e-11	0.026	86789872792.00	-0.0575	0.0980	0.5575	0.019	No	Aligned
LAP3	rs78471118	T	G	0.2020	0.0311	8.39e-11	0.022	125000000000.00	-0.0775	0.0851	0.3625	0.024	No	Aligned
LAP3	rs2192521	A	G	0.0549	0.0085	1.08e-10	0.397	1700000000000.00	-0.0142	0.0278	0.6085	0.327	No	Aligned
LAP3	rs111615986	T	C	-0.2148	0.0334	1.28e-10	0.021	59283031171.00	-0.0429	0.0533	0.4211	0.064	No	Aligned
LAP3	rs11947111	T	A	0.3413	0.0532	1.38e-10	0.028	43995404066.00	0.0187	0.0647	0.7725	0.042	Yes	Aligned
LAP3	rs76182690	T	C	0.2264	0.0353	1.41e-10	0.017	99956381367.00	0.0357	0.1293	0.7825	0.010	No	Aligned
LAP3	rs76428778	G	T	-0.2267	0.0354	1.51e-10	0.018	53169773867.00	0.0368	0.0779	0.6366	0.029	No	Aligned
LAP3	rs112016613	T	C	-0.0890	0.0144	7.30e-10	0.113	342000000000.00	0.0262	0.0330	0.4282	0.191	No	Aligned
LAP3	rs1981693	G	A	0.0597	0.0097	7.37e-10	0.757	1460000000000.00	0.0175	0.0302	0.5626	0.254	No	Flipped
LAP3	rs73232831	G	A	0.1225	0.0201	9.98e-10	0.051	348000000000.00	-0.0886	0.0711	0.2125	0.035	No	Aligned
LAP3	rs2597749	G	T	-0.0594	0.0098	1.18e-09	0.238	764000000000.00	0.0261	0.0290	0.3692	0.278	No	Flipped
LAP3	rs116188448	G	A	0.1411	0.0240	4.14e-09	0.034	266000000000.00	-0.1040	0.0829	0.2097	0.026	No	Aligned
LAP3	rs115883966	T	G	-0.2261	0.0385	4.29e-09	0.018	52147627723.00	0.0132	0.0764	0.8628	0.029	No	Aligned
LAP3	rs112374	A	G	-0.233	0.0406	8.87e-09	0.014	4870607388	0.0255	0.1514	0.8661	0.008	No	Aligned

	782			3				6.00						
LAP3	rs73241668	A	G	0.2917	0.0514	1.43e-08	0.013	63279426180.00	-0.2955	0.2037	0.1469	0.004	No	Aligned
LAP3	rs74559214	T	C	0.1748	0.0310	1.64e-08	0.023	176000000000.00	-0.1354	0.1606	0.3991	0.007	No	Aligned
LAP3	rs13148600	T	C	0.0931	0.0167	2.32e-08	0.073	625000000000.00	0.0955	0.0509	0.0605	0.075	No	Aligned
LAP3	rs145271633	C	A	-0.2324	0.0417	2.45e-08	0.013	48605078788.00	-0.1880	0.1672	0.2607	0.006	No	Aligned
LAP3	rs138278869	G	A	0.2157	0.0387	2.54e-08	0.015	117000000000.00	-0.0559	0.1512	0.7113	0.008	No	Aligned
MUC20	rs2688492	C	T	0.4918	0.0099	0.00e+00	0.259	15725832431.00	0.0317	0.0281	0.2582	0.315	No	Flipped
MUC20	rs34697430	T	C	0.3965	0.0096	0.00e+00	0.490	24403534296.00	0.0193	0.0263	0.4635	0.542	No	Flipped
MUC20	rs35845795	A	C	0.5172	0.0115	0.00e+00	0.172	14281924091.00	0.0190	0.0320	0.5532	0.207	No	Aligned
MUC20	rs78923543	G	C	0.6958	0.0172	0.00e+00	0.083	7929701511.00	0.0141	0.0422	0.7381	0.107	Yes	Aligned
MUC20	rs2550252	C	T	0.3342	0.0095	8.83e-272	0.424	34643001173.00	0.0492	0.0266	0.0648	0.386	No	Flipped
MUC20	rs67175307	T	C	-0.5587	0.0229	6.81e-131	0.044	10794336645.00	0.0125	0.0497	0.8015	0.076	No	Aligned
MUC20	rs7633151	A	G	0.2224	0.0108	5.18e-94	0.528	81569889658.00	-0.0436	0.0261	0.0954	0.488	No	Flipped
MUC20	rs4927707	T	C	-0.2053	0.0101	4.00e-91	0.292	78659687937.00	-0.0449	0.0278	0.1070	0.333	No	Flipped
MUC20	rs61306983	C	T	-0.2622	0.0136	1.44e-82	0.119	48015451390.00	-0.0662	0.0403	0.1006	0.117	No	Aligned
MUC20	rs73212254	T	C	-0.2893	0.0151	1.43e-81	0.884	39396784032.00	-0.0636	0.0372	0.0871	0.146	No	Aligned
MUC20	rs35270284	T	C	0.2104	0.0110	4.64e-81	0.805	91830591850.00	0.0653	0.0319	0.0407	0.217	No	Aligned
MUC20	rs79229103	T	C	0.7237	0.0382	4.38e-80	0.020	7770332567.00	0.1060	0.0777	0.1726	0.029	No	Aligned
MUC20	rs4927708	C	T	0.1701	0.0091	1.14e-78	0.598	141000000000.00	0.0291	0.0265	0.2718	0.447	No	Aligned
MUC20	rs9819459	T	C	-0.2105	0.0121	2.43e-67	0.753	73676174314.00	-0.0205	0.0292	0.4839	0.277	No	Aligned
MUC20	rs4677688	T	C	0.1606	0.0093	6.77e-67	0.563	159000000000.00	-0.0013	0.0263	0.9602	0.453	No	Aligned
MUC20	rs41298099	C	T	0.5496	0.0325	5.10e-64	0.025	13658807569.00	0.0223	0.0691	0.7468	0.038	No	Aligned
MUC20	rs115645306	T	C	0.6925	0.0418	1.28e-61	0.017	8625482718.00	0.0256	0.0688	0.7100	0.038	No	Aligned
MUC20	rs4927711	T	C	-0.1639	0.0101	1.37e-59	0.352	121000000000.00	0.0037	0.0274	0.8912	0.370	No	Aligned

MUC20	rs11916483	C	T	0.4626	0.0286	1.07e-58	0.027	19390566915.00	-0.0614	0.0762	0.4205	0.030	No	Aligned
MUC20	rs72611105	C	T	0.2979	0.0203	8.21e-49	0.943	47381611460.00	0.0274	0.0559	0.6235	0.057	No	Aligned
MUC20	rs11922971	A	G	-0.1918	0.0133	2.98e-47	0.853	86788930945.00	-0.0048	0.0355	0.8928	0.167	No	Aligned
MUC20	rs762010019	T	C	-0.4249	0.0312	3.11e-42	0.026	17555040271.00	0.0336	0.1121	0.7641	0.014	No	Aligned
MUC20	rs2241413	C	T	0.1288	0.0095	8.08e-42	0.463	25600000000.00	-0.0039	0.0262	0.8819	0.471	No	Aligned
MUC20	rs6804171	A	G	0.1295	0.0104	1.04e-35	0.514	25700000000.00	0.0207	0.0268	0.4389	0.520	No	Aligned
MUC20	rs147508067	C	T	0.5683	0.0484	8.18e-32	0.019	13508352303.00	-0.1070	0.1134	0.3455	0.013	No	Aligned
MUC20	rs115743766	A	G	0.4016	0.0346	3.32e-31	0.021	27108271000.00	-0.1605	0.0885	0.0698	0.023	No	Aligned
MUC20	rs7610708	T	G	0.1320	0.0117	2.06e-29	0.212	25200000000.00	0.0342	0.0308	0.2663	0.243	No	Aligned
MUC20	rs11927474	C	A	-0.1540	0.0139	2.19e-28	0.166	12900000000.00	-0.0505	0.0338	0.1352	0.185	No	Aligned
MUC20	rs76863178	C	T	-0.3010	0.0287	1.15e-25	0.032	33581355295.00	0.1195	0.0817	0.1437	0.027	No	Aligned
MUC20	rs77106593	A	G	-0.4314	0.0421	1.35e-24	0.015	16287528501.00	0.0120	0.0906	0.8947	0.022	No	Aligned
MUC20	rs77176301	C	T	-0.2400	0.0235	1.83e-24	0.042	52591969233.00	-0.0569	0.0885	0.5205	0.022	No	Aligned
MUC20	rs116854542	A	C	-0.6112	0.0623	1.03e-22	0.028	8050691882.00	0.0573	0.0638	0.3684	0.046	No	Aligned
MUC20	rs56260729	A	G	0.1069	0.0111	5.51e-22	0.208	39800000000.00	0.0398	0.0304	0.1900	0.248	No	Aligned
MUC20	rs140843768	T	C	0.3192	0.0334	1.29e-21	0.022	44724902174.00	-0.0754	0.0826	0.3613	0.025	No	Aligned
MUC20	rs7627155	T	C	0.1157	0.0121	1.61e-21	0.151	34100000000.00	0.0090	0.0394	0.8192	0.125	No	Aligned
MUC20	rs143060720	T	C	-0.4332	0.0455	1.75e-21	0.016	15938074849.00	-0.0081	0.0969	0.9330	0.018	No	Aligned
MUC20	rs13066800	G	A	0.2245	0.0239	5.55e-21	0.048	90748044361.00	0.0613	0.0525	0.2427	0.066	No	Aligned
MUC20	rs147180676	G	T	-0.2807	0.0299	6.65e-21	0.028	37849219638.00	0.0919	0.0809	0.2558	0.027	No	Aligned
MUC20	rs147180676	G	T	-0.2807	0.0299	6.65e-21	0.028	37849219638.00	0.0919	0.0809	0.2558	0.027	No	Aligned
MUC20	rs60656840	G	A	-0.0997	0.0107	1.04e-20	0.211	30000000000.00	0.0271	0.0338	0.4221	0.182	No	Aligned
MUC20	rs115160556	T	C	0.3335	0.0370	2.01e-19	0.019	41546481771.00	0.0780	0.0867	0.3685	0.024	No	Aligned
MU	rs14	C	T	0.117	0.01	1.38	0.155	33500	0.0310	0.03	0.3	0.197	No	Align

C20	6159581			7	34	e-18		0000000.00		34	543			ed
MUC20	rs150297461	C	A	0.3431	0.0391	1.57e-18	0.020	39492580369.00	0.1787	0.1167	0.1256	0.013	No	Aligned
MUC20	rs34049168	A	G	0.1088	0.0127	1.16e-17	0.203	395000000000.00	0.0106	0.0325	0.7443	0.222	No	Aligned
MUC20	rs7432894	C	T	-0.0782	0.0093	3.78e-17	0.328	477000000000.00	-0.0128	0.0293	0.6622	0.272	No	Flipped
MUC20	rs60568302	A	G	0.2015	0.0244	1.57e-16	0.034	116000000000.00	-0.0147	0.0731	0.8404	0.033	No	Aligned
MUC20	rs151192980	G	A	0.2122	0.0259	2.43e-16	0.036	105000000000.00	0.1518	0.1068	0.1551	0.016	No	Aligned
MUC20	rs4677802	G	A	0.0830	0.0102	3.21e-16	0.686	688000000000.00	-0.0287	0.0296	0.3323	0.271	No	Flipped
MUC20	rs28651009	T	C	0.0781	0.0097	7.99e-16	0.356	780000000000.00	-0.0483	0.0275	0.0794	0.365	No	Flipped
MUC20	rs11712192	A	G	-0.0911	0.0117	6.18e-15	0.207	345000000000.00	-0.0486	0.0311	0.1181	0.232	No	Aligned
MUC20	rs78961457	G	A	-0.1540	0.0198	6.94e-15	0.054	121000000000.00	-0.0007	0.0668	0.9912	0.041	No	Aligned
MUC20	rs7630048	A	G	0.0723	0.0094	1.17e-14	0.326	923000000000.00	0.0427	0.0298	0.1515	0.260	No	Flipped
MUC20	rs115786032	T	C	0.3526	0.0462	2.38e-14	0.015	38902913938.00	-0.1647	0.1541	0.2852	0.007	No	Aligned
MUC20	rs68143103	A	G	0.2823	0.0371	2.75e-14	0.020	60721152241.00	0.0579	0.1499	0.6994	0.008	No	Aligned
MUC20	rs823532	C	T	0.0783	0.0105	7.87e-14	0.672	793000000000.00	-0.0133	0.0270	0.6218	0.383	No	Aligned
MUC20	rs35559909	G	A	0.1205	0.0165	2.46e-13	0.093	337000000000.00	0.0032	0.0405	0.9367	0.121	No	Aligned
MUC20	rs7340710	T	C	-0.1183	0.0162	2.84e-13	0.079	202000000000.00	0.0487	0.0440	0.2685	0.096	No	Aligned
MUC20	rs146105953	C	T	0.3706	0.0515	6.40e-13	0.019	35870273219.00	0.0836	0.0773	0.2796	0.029	No	Aligned
MUC20	rs112242983	A	C	-0.1059	0.0149	1.41e-12	0.094	250000000000.00	-0.0590	0.0471	0.2105	0.086	No	Aligned
MUC20	rs114126286	C	T	-0.3701	0.0539	6.43e-12	0.015	20316620193.00	-0.0254	0.1121	0.8207	0.014	No	Aligned
MUC20	rs4927887	T	C	0.0648	0.0099	5.49e-11	0.359	1210000000000.00	0.0074	0.0266	0.7798	0.405	No	Aligned
MUC20	rs2056469	G	A	-0.0978	0.0151	8.46e-11	0.111	287000000000.00	0.0255	0.0468	0.5863	0.086	No	Aligned
MUC20	rs9876236	A	G	0.2157	0.0335	1.19e-10	0.025	110000000000.00	-0.0182	0.1039	0.8609	0.016	No	Aligned
MUC20	rs2686442	G	A	0.0920	0.0143	1.20e-10	0.299	605000000000.00	0.0307	0.0283	0.2784	0.346	No	Aligned
MUC20	rs800873	T	C	-0.220	0.0344	1.45e-10	0.022	5623885958	0.1787	0.1173	0.1275	0.013	No	Aligned

	24			4				3.00						
MUC20	rs2410802	G	A	-0.1995	0.0318	3.53e-10	0.023	68226607805.00	0.0520	0.0764	0.4964	0.030	No	Aligned
MUC20	rs146199320	G	A	0.1684	0.0270	4.40e-10	0.032	1830000000.00	-0.3681	0.0995	0.0002	0.019	No	Aligned
MUC20	rs9851595	T	C	0.0563	0.0091	6.42e-10	0.628	16400000000.00	0.0578	0.0266	0.0299	0.400	No	Aligned
MUC20	rs9864994	A	C	0.0573	0.0093	7.64e-10	0.663	15900000000.00	0.0247	0.0286	0.3873	0.302	No	Aligned
MUC20	rs62283270	C	T	0.0782	0.0127	8.68e-10	0.654	8530000000.00	-0.0138	0.0280	0.6216	0.357	No	Aligned
MUC20	rs73206633	G	A	-0.0770	0.0128	1.85e-09	0.866	4520000000.00	0.0427	0.0385	0.2674	0.131	No	Aligned
MUC20	rs56109345	G	A	-0.0533	0.0090	3.05e-09	0.611	9420000000.00	0.0113	0.0268	0.6738	0.369	No	Aligned
MUC20	rs114439325	A	G	-0.3336	0.0565	3.56e-09	0.022	23992316511.00	0.0090	0.0724	0.9008	0.035	No	Aligned
MUC20	rs55824736	T	C	-0.2334	0.0398	4.37e-09	0.022	48947617144.00	-0.0326	0.0772	0.6733	0.030	No	Aligned
MUC20	rs34288633	C	T	-0.0763	0.0130	4.61e-09	0.232	4580000000.00	0.0344	0.0368	0.3496	0.160	No	Aligned
MUC20	rs79398212	C	T	0.1189	0.0203	4.65e-09	0.052	3760000000.00	0.0371	0.0667	0.5780	0.040	No	Aligned
MUC20	rs116492543	T	C	-0.2034	0.0358	1.35e-08	0.025	63817297682.00	0.0243	0.0599	0.6849	0.051	No	Aligned
MUC20	rs1056726	A	G	0.1362	0.0242	1.84e-08	0.042	2910000000.00	-0.1213	0.0689	0.0781	0.038	No	Aligned
MUC20	rs78292793	T	C	-0.1195	0.0213	2.10e-08	0.052	1840000000.00	-0.0599	0.0581	0.3025	0.055	No	Aligned
MUC20	rs141363947	A	G	-0.3309	0.0596	2.80e-08	0.022	23945734153.00	0.1224	0.0820	0.1353	0.026	No	Aligned
MUC20	rs62408947	A	G	-0.0824	0.0149	3.01e-08	0.095	3860000000.00	0.0563	0.0413	0.1731	0.115	No	Aligned
MUC20	rs9870813	C	T	0.0485	0.0088	3.88e-08	0.409	23200000000.00	0.0124	0.0266	0.6408	0.422	No	Flipped
MUC20	rs6806773	A	G	-0.0570	0.0105	4.86e-08	0.224	8010000000.00	-0.0180	0.0287	0.5318	0.296	No	Flipped
TNK2	rs2278034	C	T	0.1057	0.0087	3.41e-34	0.508	3880000000.00	0.0382	0.0262	0.1459	0.494	No	Aligned
TNK2	rs11713826	G	T	0.1981	0.0219	1.43e-19	0.044	1180000000.00	0.0863	0.0763	0.2579	0.031	No	Aligned
TNK2	rs147180676	G	T	0.2445	0.0299	3.04e-16	0.028	79277979822.00	0.0919	0.0809	0.2558	0.027	No	Aligned
TNK2	rs147180676	G	T	0.2445	0.0299	3.04e-16	0.028	79277979822.00	0.0919	0.0809	0.2558	0.027	No	Aligned
TNK2	rs34530970	T	C	0.0807	0.0130	5.24e-10	0.132	7970000000.00	0.0487	0.0354	0.1691	0.163	No	Aligned

TNK2	rs2278032	A	G	0.0749	0.0126	2.62e-09	0.177	94100000000.00	0.0401	0.0361	0.2666	0.159	No	Aligned

Note: Beta_outcome_aligned and EAF_outcome_aligned were aligned to the effect allele at the exposure level (EA_exposure); Allele_alignment = Flipped indicates that the outcome allele is in the opposite direction to the exposure allele.

Supplementary Table S3. MR genes significantly associated with MASLD outcomes (IVW, p<0.05; n=9)

Gene	Outcome	nSNP	OR	CI_low	CI_high	P	FDR-adjusted P value	Direction
IGFBP7	MASLD	27	0.712	0.575	0.881	0.0018	0.0078	Protective (OR<1)
MUC20	MASLD	82	1.111	1.038	1.189	0.0024	0.0078	Risk (OR>1)
KYNU	MASLD	20	1.296	1.095	1.535	0.0026	0.0078	Risk (OR>1)
ETV7	MASLD	46	1.161	1.041	1.296	0.0075	0.0169	Risk (OR>1)
ANK3	MASLD	54	1.134	1.019	1.261	0.0213	0.0338	Risk (OR>1)
VSIG10	MASLD	36	0.855	0.747	0.978	0.0225	0.0338	Protective (OR<1)
ABCC3	MASLD	59	0.909	0.831	0.995	0.0377	0.0467	Protective (OR<1)
THBS1	MASLD	38	0.879	0.776	0.995	0.0415	0.0467	Protective (OR<1)
ECHDC3	MASLD	34	1.139	1.001	1.297	0.0483	0.0483	Risk (OR>1)

Note: OR, odds ratio; CI, confidence interval; nSNP, number of instrumental variables; IVW, inverse-variance weighted; FDR, false discovery rate. FDR-adjusted P values were calculated using the Benjamini–Hochberg method based on the IVW P values. OR > 1 indicates a risk effect, and OR < 1 indicates a protective effect.

Supplementary Table S4. Instrumental Variable SNPs and Exposure/Outcome Effects of Genes Significantly Associated with MASLD Outcomes (Aligned by Allele; n=399 rows)

Gene	SNP	EA_exposure	OA_exposure	Beta_exposure	SE_exposure	P_exposure	EAF_exposure	F_value	Beta_outcome_aligned	SE_outcome	P_outcome	EAF_outcome_aligned	Palindromic	Allele_alignment
ABCC3	rs2189595	G	A	0.4117	0.0083	0.00e+00	0.379	22431632689.00	-0.0256	0.0515	0.6184	0.322	No	Flipped
ABCC3	rs2240802	A	G	0.3578	0.0087	0.00e+00	0.703	29966962380.00	-0.0439	0.0534	0.4110	0.281	No	Aligned
ABCC3	rs739923	A	G	-0.4111	0.0090	0.00e+00	0.264	20690525413.00	0.0465	0.0504	0.3562	0.351	No	Aligned
ABCC3	rs11079922	C	T	0.3298	0.0107	3.61e-208	0.165	35866906822.00	-0.0153	0.0791	0.8470	0.102	No	Aligned
ABCC3	rs73344347	C	G	0.4245	0.0143	3.84e-193	0.085	21700015788.00	0.0961	0.1167	0.4103	0.044	Yes	Aligned
ABCC3	rs61479331	G	T	0.2550	0.0096	7.30e-154	0.219	60675194030.00	-0.0200	0.0576	0.7285	0.224	No	Aligned
ABCC3	rs12451964	G	A	-0.2886	0.0117	2.41e-135	0.138	40508594263.00	0.0996	0.0652	0.1268	0.162	No	Aligned
AB	rs11	A	G	-	0.01	2.65	0.045	16265	0.0063	0.15	0.96	0.023	No	Align

CC3	568581			0.4540	98	e-116		845216.00		77	80			ed
ABCC3	rs72837520	A	G	0.4000	0.0175	2.36e-115	0.057	2496407468 4.00	-0.1050	0.0971	0.2796	0.066	No	Aligned
ABCC3	rs1989983	A	G	0.2819	0.0129	2.18e-106	0.125	5044272756 5.00	0.0770	0.0716	0.2822	0.129	No	Aligned
ABCC3	rs80063067	C	T	0.2928	0.0161	2.94e-74	0.073	4767789348 5.00	-0.0371	0.1270	0.7700	0.037	No	Aligned
ABCC3	rs117355256	T	G	-0.3389	0.0186	3.16e-74	0.055	2858002348 1.00	0.0677	0.0985	0.4917	0.065	No	Aligned
ABCC3	rs116972558	T	C	-0.3787	0.0220	2.40e-66	0.043	2273837858 7.00	0.0498	0.0879	0.5713	0.081	No	Aligned
ABCC3	rs12601390	T	G	0.4596	0.0287	7.37e-58	0.024	1966206758 2.00	0.0388	0.1335	0.7714	0.034	No	Aligned
ABCC3	rs4794168	G	A	0.1433	0.0089	1.00e-57	0.639	2020000000 00.00	-0.0582	0.0503	0.2471	0.354	No	Flipped
ABCC3	rs55870403	A	G	-0.2786	0.0189	4.51e-49	0.055	4126119193 9.00	-0.0155	0.0881	0.8602	0.083	No	Aligned
ABCC3	rs12602060	A	G	-0.1364	0.0096	3.14e-46	0.224	1720000000 00.00	0.0186	0.0571	0.7441	0.230	No	Aligned
ABCC3	rs113400824	A	G	-0.3438	0.0253	4.53e-42	0.028	2680176340 2.00	0.0561	0.1551	0.7174	0.024	No	Aligned
ABCC3	rs76521597	A	G	-0.2782	0.0205	6.03e-42	0.043	4093328363 4.00	-0.0584	0.1091	0.5926	0.052	No	Aligned
ABCC3	rs4793661	A	G	0.1496	0.0111	1.58e-41	0.152	1900000000 00.00	0.0436	0.0754	0.5626	0.114	No	Aligned
ABCC3	rs141274954	C	T	-0.1850	0.0142	1.23e-38	0.090	9202733018 5.00	-0.0231	0.0754	0.7595	0.115	No	Aligned
ABCC3	rs145058193	C	T	0.4712	0.0372	8.14e-37	0.017	1938489129 5.00	-0.5181	0.3605	0.1507	0.004	No	Aligned
ABCC3	rs2526535	A	G	-0.1086	0.0086	1.04e-36	0.311	2660000000 00.00	0.0314	0.0509	0.5376	0.333	No	Flipped
ABCC3	rs117601101	T	G	0.4309	0.0347	2.57e-35	0.017	2327019949 8.00	-0.3068	0.2193	0.1618	0.012	No	Aligned
ABCC3	rs74710086	A	G	-0.4647	0.0383	8.32e-34	0.016	1442892848 8.00	0.1988	0.1317	0.1314	0.035	No	Aligned
ABCC3	rs62061895	T	C	0.2805	0.0247	7.59e-30	0.033	5581402697 2.00	-0.1848	0.1215	0.1284	0.041	No	Aligned
ABCC3	rs11653728	A	T	0.0880	0.0084	6.83e-26	0.653	5760000000 00.00	-0.0435	0.0500	0.3843	0.355	Yes	Aligned
ABCC3	rs72842223	A	G	-0.2529	0.0245	6.77e-25	0.032	4743397420 8.00	0.1374	0.1120	0.2201	0.049	No	Aligned
ABCC	rs14957	A	G	0.4044	0.0410	6.68e-23	0.014	2765749961	-0.0485	0.2476	0.8448	0.009	No	Aligned

3	0333							3.00						
ABCC3	rs141032183	A	G	-0.4163	0.0425	1.16e-22	0.015	17347478764.00	-0.1727	0.2359	0.4640	0.011	No	Aligned
ABCC3	rs78056364	A	G	0.3749	0.0391	9.00e-22	0.016	32382243110.00	0.1856	0.2768	0.5026	0.007	No	Aligned
ABCC3	rs9898731	T	C	0.0812	0.0086	3.17e-21	0.448	6920000000000.00	0.0357	0.0485	0.4615	0.423	No	Aligned
ABCC3	rs887377	T	C	0.1049	0.0119	1.40e-18	0.144	4230000000000.00	-0.0717	0.0628	0.2540	0.177	No	Flipped
ABCC3	rs34958340	T	C	-0.4876	0.0564	5.39e-18	0.013	12338453696.00	0.0951	0.2979	0.7495	0.007	No	Aligned
ABCC3	rs77449141	A	G	0.1299	0.0154	3.61e-17	0.076	2790000000000.00	0.0606	0.0927	0.5134	0.072	No	Aligned
ABCC3	rs198556	T	G	0.1590	0.0192	1.29e-16	0.049	1870000000000.00	0.0107	0.1088	0.9218	0.051	No	Aligned
ABCC3	rs879778	T	C	-0.0785	0.0096	3.97e-16	0.258	4710000000000.00	-0.0013	0.0545	0.9805	0.270	No	Aligned
ABCC3	rs34817726	A	G	-0.0778	0.0098	2.20e-15	0.741	4760000000000.00	0.0827	0.0539	0.1250	0.270	No	Aligned
ABCC3	rs2412325	C	G	-0.0752	0.0096	3.55e-15	0.224	5080000000000.00	0.0664	0.0585	0.2565	0.214	Yes	Aligned
ABCC3	rs112819464	T	C	0.3610	0.0461	5.00e-15	0.015	36830619838.00	-0.0707	0.2224	0.7505	0.012	No	Aligned
ABCC3	rs77651559	A	G	0.3931	0.0506	7.81e-15	0.016	31123433871.00	0.1662	0.2846	0.5594	0.007	No	Aligned
ABCC3	rs198551	A	G	-0.0616	0.0080	1.14e-14	0.452	7530000000000.00	-0.0153	0.0482	0.7505	0.440	No	Flipped
ABCC3	rs113287138	G	T	0.4156	0.0544	2.23e-14	0.016	27989852474.00	0.0338	0.2675	0.8995	0.008	No	Aligned
ABCC3	rs142932106	A	G	-0.3320	0.0442	5.75e-14	0.016	25811149725.00	0.0846	0.3678	0.8182	0.004	No	Aligned
ABCC3	rs4257246	A	G	0.1985	0.0268	1.30e-13	0.032	1240000000000.00	0.0352	0.1597	0.8255	0.024	No	Aligned
ABCC3	rs56994118	C	T	-0.0772	0.0105	1.71e-13	0.174	4750000000000.00	0.0241	0.0669	0.7186	0.152	No	Aligned
ABCC3	rs62059698	G	A	-0.0728	0.0102	1.06e-12	0.185	5290000000000.00	0.0045	0.0565	0.9372	0.235	No	Aligned
ABCC3	rs141252368	T	C	0.2259	0.0321	1.87e-12	0.019	97199307293.00	0.0276	0.2314	0.9052	0.011	No	Aligned
ABCC3	rs183428517	T	G	-0.2196	0.0324	1.21e-11	0.037	57498955185.00	-0.6449	0.2822	0.0223	0.007	No	Aligned
ABCC	rs14477	A	G	0.2808	0.0424	3.47e-11	0.019	6426074277	0.0420	0.1175	0.7208	0.045	No	Aligned

3	8037							6.00						
ABCC3	rs55768061	T	C	-0.1278	0.0195	5.93e-11	0.051	1680000000000.00	0.0661	0.0958	0.4903	0.067	No	Aligned
ABCC3	rs7213655	G	C	0.0621	0.0095	6.63e-11	0.230	1320000000000.00	-0.0593	0.0531	0.2648	0.285	Yes	Aligned
ABCC3	rs8065514	A	G	0.0835	0.0131	1.61e-10	0.105	7360000000000.00	0.0570	0.0838	0.4966	0.089	No	Flipped
ABCC3	rs139941293	T	C	-0.2973	0.0470	2.47e-10	0.019	30806532962.00	-0.1414	0.1516	0.3511	0.026	No	Aligned
ABCC3	rs4148408	A	G	-0.1139	0.0183	5.29e-10	0.050	2090000000000.00	-0.0749	0.0927	0.4187	0.072	No	Aligned
ABCC3	rs74869391	A	G	0.2220	0.0368	1.64e-09	0.015	1070000000000.00	0.1263	0.1942	0.5154	0.016	No	Aligned
ABCC3	rs12938101	T	A	-0.0534	0.0090	2.73e-09	0.342	9380000000000.00	0.0517	0.0497	0.2983	0.373	Yes	Aligned
ABCC3	rs150333964	T	G	0.1400	0.0242	6.96e-09	0.030	2720000000000.00	-0.2475	0.1413	0.0799	0.030	No	Aligned
ABCC3	rs1105343	C	A	-0.1347	0.0233	7.24e-09	0.031	1460000000000.00	0.1306	0.1508	0.3867	0.026	No	Aligned
ANK3	rs11817236	G	A	-0.3441	0.0080	0.00e+00	0.521	29453778951.00	-0.0768	0.0482	0.1112	0.460	No	Aligned
ANK3	rs16915196	G	A	-0.4895	0.0116	0.00e+00	0.138	14547511924.00	-0.0571	0.0685	0.4050	0.143	No	Aligned
ANK3	rs7905531	T	C	0.3138	0.0082	0.00e+00	0.376	39100466014.00	0.0726	0.0505	0.1503	0.341	No	Aligned
ANK3	rs114768836	G	T	0.5508	0.0197	2.06e-172	0.044	12944302770.00	0.0421	0.1492	0.7777	0.026	No	Aligned
ANK3	rs78581455	C	T	-0.6559	0.0239	2.47e-165	0.032	7901200084.00	0.1406	0.1484	0.3434	0.028	No	Aligned
ANK3	rs10994296	G	A	-0.2812	0.0144	1.40e-84	0.083	41786312266.00	-0.0307	0.0657	0.6406	0.160	No	Aligned
ANK3	rs57450815	C	T	-0.2196	0.0115	6.69e-82	0.143	68413328056.00	-0.0755	0.0671	0.2608	0.151	No	Aligned
ANK3	rs6479706	G	A	0.1547	0.0086	1.73e-72	0.314	1710000000000.00	0.0360	0.0516	0.4863	0.317	No	Flipped
ANK3	rs61856263	T	C	-0.2324	0.0131	4.34e-70	0.103	60586379510.00	-0.0943	0.0662	0.1545	0.156	No	Aligned
ANK3	rs79867207	C	T	-0.3582	0.0216	5.52e-62	0.037	25326154405.00	-0.0130	0.1441	0.9280	0.029	No	Aligned
ANK3	rs2279942	T	C	-0.1314	0.0088	1.28e-50	0.710	1860000000000.00	0.0545	0.0521	0.2955	0.302	No	Aligned
ANK3	rs78839797	C	T	-0.1879	0.0128	4.81e-49	0.111	90709145177.00	0.0024	0.0737	0.9737	0.126	No	Aligned
ANK3	rs56068	C	G	-0.115	0.0085	2.71e-42	0.333	2380000000	-0.0147	0.0484	0.7613	0.420	Yes	Aligned

	978			5				00.00						
ANK3	rs12784166	G	A	-0.2797	0.0216	2.92e-38	0.038	40218573221.00	-0.0710	0.1036	0.4935	0.057	No	Aligned
ANK3	rs55683151	C	T	-0.4303	0.0342	2.60e-36	0.018	16922686267.00	0.0498	0.2717	0.8546	0.008	No	Aligned
ANK3	rs117026052	A	G	-0.8452	0.0679	1.46e-35	0.013	4379449003.00	-0.5443	0.5176	0.2930	0.002	No	Aligned
ANK3	rs10821767	A	T	0.1513	0.0124	3.98e-34	0.883	1890000000000.00	0.0162	0.0763	0.8315	0.110	Yes	Aligned
ANK3	rs4542352	T	C	-0.0970	0.0082	1.42e-32	0.397	3300000000000.00	0.0255	0.0491	0.6033	0.387	No	Flipped
ANK3	rs10994281	G	A	-0.1193	0.0102	9.45e-32	0.203	2180000000000.00	-0.0525	0.0590	0.3738	0.213	No	Aligned
ANK3	rs141837303	A	G	0.2559	0.0223	2.23e-30	0.034	66945639912.00	0.1869	0.1441	0.1947	0.029	No	Aligned
ANK3	rs55838521	G	A	0.1291	0.0116	7.29e-29	0.140	2640000000000.00	0.0021	0.0774	0.9781	0.107	No	Aligned
ANK3	rs7910802	A	G	-0.2415	0.0218	1.38e-28	0.038	52702513713.00	-0.2232	0.1313	0.0892	0.034	No	Aligned
ANK3	rs3793860	A	G	-0.1909	0.0177	3.25e-27	0.054	83975770773.00	-0.1483	0.1040	0.1538	0.057	No	Aligned
ANK3	rs3213025	C	T	0.1201	0.0114	4.37e-26	0.146	3090000000000.00	0.0661	0.0603	0.2734	0.197	No	Aligned
ANK3	rs7073658	T	G	-0.0839	0.0081	4.07e-25	0.414	4310000000000.00	-0.0288	0.0481	0.5491	0.486	No	Flipped
ANK3	rs79102984	T	C	0.1791	0.0177	3.62e-24	0.058	1400000000000.00	0.1116	0.1242	0.3688	0.038	No	Aligned
ANK3	rs3213017	T	G	-0.2801	0.0280	1.63e-23	0.023	38457943751.00	0.3728	0.3038	0.2198	0.006	No	Aligned
ANK3	rs10994453	T	G	0.2052	0.0213	6.20e-22	0.039	1080000000000.00	-0.0754	0.1543	0.6252	0.025	No	Aligned
ANK3	rs72811877	G	A	0.1696	0.0189	2.50e-19	0.058	1610000000000.00	0.0125	0.1027	0.9029	0.058	No	Aligned
ANK3	rs80263212	A	T	-0.1747	0.0199	1.97e-18	0.045	96388204403.00	0.1552	0.1042	0.1366	0.056	Yes	Aligned
ANK3	rs138052944	G	A	-0.3163	0.0374	2.95e-17	0.015	29189848598.00	-0.5094	0.2714	0.0605	0.008	No	Aligned
ANK3	rs41274674	A	G	-0.1779	0.0213	7.49e-17	0.039	91969226738.00	0.0359	0.1086	0.7406	0.052	No	Aligned
ANK3	rs10740011	G	T	0.0658	0.0080	2.04e-16	0.454	10900000000000.00	0.0063	0.0489	0.8969	0.423	No	Flipped
ANK3	rs112686360	C	T	0.1784	0.0219	3.38e-16	0.037	1490000000000.00	-0.2555	0.1761	0.1467	0.019	No	Aligned
ANK3	rs79217	A	G	-0.154	0.0190	4.40e-16	0.051	1210000000	-0.0553	0.1294	0.6690	0.036	No	Aligned

	840			8				00.00						
ANK3	rs149297290	C	T	-0.1797	0.0224	1.01e-15	0.034	89403693157.00	0.1095	0.1434	0.4450	0.029	No	Aligned
ANK3	rs6479688	G	A	-0.0651	0.0082	2.95e-15	0.376	6790000000000.00	-0.0284	0.0482	0.5559	0.444	No	Aligned
ANK3	rs75026145	T	C	-0.2312	0.0295	5.16e-15	0.024	53724340304.00	-0.2105	0.3005	0.4837	0.007	No	Aligned
ANK3	rs140656002	C	T	-0.1912	0.0245	5.68e-15	0.031	78484163047.00	0.0449	0.1509	0.7663	0.026	No	Aligned
ANK3	rs3213056	A	G	0.1518	0.0207	2.54e-13	0.046	2130000000000.00	0.0328	0.1316	0.8030	0.034	No	Aligned
ANK3	rs73261169	G	T	0.0778	0.0106	2.58e-13	0.177	8090000000000.00	0.0281	0.0692	0.6848	0.144	No	Aligned
ANK3	rs76667912	T	C	0.0771	0.0106	4.38e-13	0.826	8270000000000.00	-0.0331	0.0640	0.6051	0.169	No	Aligned
ANK3	rs10994141	C	A	-0.1979	0.0279	1.33e-12	0.031	71646773157.00	0.0285	0.0916	0.7555	0.075	No	Aligned
ANK3	rs75686970	T	C	-0.1831	0.0263	3.31e-12	0.026	83322189857.00	0.1253	0.1607	0.4355	0.023	No	Aligned
ANK3	rs1183347	T	C	0.0614	0.0090	6.94e-12	0.274	13300000000000.00	-0.1130	0.0587	0.0542	0.211	No	Flipped
ANK3	rs10994406	C	T	0.1849	0.0277	2.67e-11	0.024	1480000000000.00	0.0372	0.1530	0.8080	0.026	No	Aligned
ANK3	rs10821683	C	T	0.0885	0.0134	4.34e-11	0.098	6480000000000.00	-0.0837	0.1013	0.4084	0.059	No	Aligned
ANK3	rs117908493	G	A	-0.1961	0.0306	1.42e-10	0.020	71057951755.00	-0.2736	0.2184	0.2102	0.012	No	Aligned
ANK3	rs10821844	A	G	-0.0533	0.0084	2.30e-10	0.362	9580000000000.00	0.0246	0.0495	0.6196	0.384	No	Aligned
ANK3	rs9415604	A	G	0.1288	0.0207	4.77e-10	0.042	3120000000000.00	0.0173	0.1214	0.8869	0.040	No	Aligned
ANK3	rs146953336	T	G	-0.3689	0.0602	8.88e-10	0.013	19837370875.00	0.1066	0.3771	0.7775	0.004	No	Aligned
ANK3	rs117717606	A	G	-0.3373	0.0582	6.80e-09	0.013	23340268772.00	0.0583	0.3688	0.8743	0.004	No	Aligned
ANK3	rs10994282	G	A	0.0659	0.0116	1.48e-08	0.145	12400000000000.00	-0.0720	0.0705	0.3068	0.135	No	Aligned
ANK3	rs76932066	A	G	0.0766	0.0137	2.10e-08	0.098	9230000000000.00	-0.0381	0.0902	0.6730	0.080	No	Aligned
ECHDC3	rs718641	C	T	-0.4659	0.0086	0.00e+00	0.683	16224082228.00	-0.0805	0.0523	0.1241	0.300	No	Flipped
ECHDC3	rs11257331	A	G	0.1960	0.0086	1.56e-114	0.319	1040000000000.00	0.0136	0.0516	0.7923	0.318	No	Aligned
EC	rs11	G	A	0.314	0.01	1.54	0.086	40474	0.1253	0.08	0.13	0.092	No	Align

HDC3	257300			7	43	e-107		757610.00		30	10			ed
ECHDC3	rs7477429	G	A	-0.3619	0.0172	8.73e-98	0.059	25405085117.00	-0.0534	0.1111	0.6312	0.049	No	Aligned
ECHDC3	rs4747928	T	C	0.1593	0.0090	1.77e-70	0.270	16200000000.00	0.0525	0.0531	0.3223	0.292	No	Aligned
ECHDC3	rs113165185	A	G	-0.4847	0.0283	8.36e-66	0.024	13877935439.00	-0.1968	0.1489	0.1864	0.027	No	Aligned
ECHDC3	rs60806452	A	G	-0.3474	0.0214	2.40e-59	0.039	26845423559.00	-0.0557	0.1044	0.5937	0.056	No	Aligned
ECHDC3	rs76046976	T	G	-0.4615	0.0317	6.08e-48	0.020	15007661053.00	-0.1762	0.1569	0.2616	0.025	No	Aligned
ECHDC3	rs118165782	A	G	-0.2752	0.0193	3.63e-46	0.045	42090955792.00	-0.0370	0.1120	0.7407	0.050	No	Aligned
ECHDC3	rs36066784	A	G	-0.1619	0.0114	3.80e-46	0.145	12200000000.00	0.1139	0.0601	0.0583	0.197	No	Aligned
ECHDC3	rs11257236	G	A	0.1077	0.0083	8.37e-39	0.372	37000000000.00	-0.0101	0.0500	0.8404	0.360	No	Aligned
ECHDC3	rs17150721	G	C	-0.1136	0.0089	1.10e-37	0.284	24300000000.00	0.1085	0.0510	0.0334	0.332	Yes	Aligned
ECHDC3	rs79044909	T	C	0.2783	0.0220	1.24e-36	0.034	55599439053.00	-0.1042	0.1178	0.3763	0.043	No	Aligned
ECHDC3	rs4747939	G	T	-0.5174	0.0424	2.84e-34	0.014	11653524643.00	0.0876	0.1977	0.6578	0.015	No	Aligned
ECHDC3	rs139899818	A	G	0.2417	0.0222	1.74e-27	0.035	75847724542.00	-0.0668	0.1763	0.7049	0.019	No	Aligned
ECHDC3	rs2399726	T	C	-0.0794	0.0087	5.39e-20	0.365	47100000000.00	0.0543	0.0513	0.2892	0.338	No	Aligned
ECHDC3	rs61844929	A	C	0.1411	0.0157	2.57e-19	0.072	23200000000.00	0.0563	0.0983	0.5671	0.064	No	Aligned
ECHDC3	rs117927419	T	C	-0.2360	0.0270	2.27e-18	0.026	52803429890.00	-0.1228	0.1449	0.3967	0.029	No	Aligned
ECHDC3	rs2440068	G	A	0.0702	0.0082	9.19e-18	0.399	94800000000.00	0.0629	0.0488	0.1981	0.426	No	Aligned
ECHDC3	rs35283504	T	C	-0.3258	0.0382	1.49e-17	0.014	27566790619.00	0.1093	0.2100	0.6027	0.013	No	Aligned
ECHDC3	rs138462460	C	T	-0.3993	0.0469	1.61e-17	0.012	18343017401.00	-0.0982	0.2015	0.6262	0.015	No	Aligned
ECHDC3	rs72779871	A	G	0.2834	0.0340	7.49e-17	0.016	58709112974.00	0.0001	0.1838	0.9995	0.018	No	Aligned
ECHDC3	rs112559601	C	T	0.1890	0.0227	7.96e-17	0.036	13200000000.00	0.1895	0.1661	0.2539	0.022	No	Aligned
ECHD	rs41311	C	T	-0.595	0.0735	4.92e-16	0.014	8149542935.	-0.0440	0.3392	0.8967	0.005	No	Aligned

C3	226			9				00						
ECHDC3	rs7083962	A	G	-0.0672	0.0084	9.45e-16	0.352	63900000000.00	-0.0037	0.0510	0.9427	0.328	No	Flipped
ECHDC3	rs7087572	A	G	0.0868	0.0121	7.90e-13	0.124	65500000000.00	0.1142	0.0724	0.1144	0.126	No	Flipped
ECHDC3	rs12416487	A	T	-0.0558	0.0083	2.20e-11	0.640	88900000000.00	0.0387	0.0495	0.4342	0.381	Yes	Flipped
ECHDC3	rs11524817	C	T	-0.0581	0.0088	4.70e-11	0.707	81600000000.00	0.0068	0.0525	0.8971	0.295	No	Flipped
ECHDC3	rs12779264	C	T	0.0894	0.0147	1.09e-09	0.094	65400000000.00	0.0645	0.0731	0.3779	0.122	No	Aligned
ECHDC3	rs147520972	G	A	-0.1628	0.0278	4.57e-09	0.025	10100000000.00	0.0306	0.2485	0.9021	0.010	No	Aligned
ECHDC3	rs76011191	T	C	-0.2070	0.0356	6.22e-09	0.016	62004752813.00	-0.1600	0.2032	0.4312	0.015	No	Aligned
ECHDC3	rs117238864	C	T	0.1139	0.0197	7.29e-09	0.050	41100000000.00	0.2101	0.0952	0.0274	0.070	No	Aligned
ECHDC3	rs115725431	C	T	0.1798	0.0313	9.21e-09	0.019	16600000000.00	-0.0233	0.1765	0.8951	0.019	No	Aligned
ECHDC3	rs142823982	C	T	0.1525	0.0266	9.94e-09	0.025	23000000000.00	-0.2293	0.1251	0.0668	0.039	No	Aligned
ETV7	rs6928048	G	A	-0.3155	0.0082	0.00e+00	0.543	34853042388.00	-0.0526	0.0484	0.2777	0.471	No	Flipped
ETV7	rs9394346	A	G	-0.4586	0.0097	0.00e+00	0.571	16653092151.00	-0.0767	0.0481	0.1108	0.482	No	Flipped
ETV7	rs13202984	G	A	0.2954	0.0088	1.07e-249	0.351	44442657944.00	0.0474	0.0502	0.3447	0.369	No	Aligned
ETV7	rs13202984	G	A	0.2954	0.0088	1.07e-249	0.351	44442657944.00	0.0474	0.0502	0.3447	0.369	No	Aligned
ETV7	rs111352602	T	C	-0.3409	0.0189	1.64e-72	0.052	28209458922.00	-0.1528	0.1003	0.1278	0.061	No	Aligned
ETV7	rs9368936	C	T	0.2939	0.0183	3.59e-58	0.056	48078460091.00	0.0179	0.1242	0.8854	0.039	No	Aligned
ETV7	rs6937326	A	G	0.2660	0.0170	5.92e-55	0.069	58910352912.00	0.0015	0.1348	0.9914	0.034	No	Aligned
ETV7	rs12201516	G	A	-0.3187	0.0205	1.20e-54	0.050	31739161837.00	0.1140	0.1061	0.2827	0.054	No	Aligned
ETV7	rs10947605	C	T	0.3313	0.0220	3.53e-51	0.042	38175285124.00	0.0381	0.1023	0.7095	0.062	No	Aligned
ETV7	rs7758422	C	T	-0.1358	0.0100	4.38e-42	0.274	17200000000.00	-0.0880	0.0530	0.0970	0.321	No	Flipped
ETV7	rs186890	A	C	-0.1154	0.0089	4.49e-38	0.720	23600000000.00	-0.0845	0.0531	0.1114	0.287	No	Flipped
ET	rs12	A	G	0.138	0.01	8.34	0.167	22400	-0.0418	0.06	0.50	0.187	No	Align

V7	198605			4	08	e-38		0000000.00		22	11			ed
ETV7	rs114910336	C	T	0.2657	0.0207	8.49e-38	0.047	60826921875.00	-0.1576	0.1302	0.2259	0.035	No	Aligned
ETV7	rs146313687	T	C	-0.2522	0.0199	6.35e-37	0.045	49329157808.00	-0.1704	0.1077	0.1135	0.053	No	Aligned
ETV7	rs112548021	C	T	0.2999	0.0245	1.67e-34	0.034	48153346701.00	0.2163	0.1395	0.1208	0.032	No	Aligned
ETV7	rs114892399	A	G	-0.3294	0.0281	8.45e-32	0.025	28580704555.00	-0.1991	0.1844	0.2803	0.017	No	Aligned
ETV7	rs236491	T	A	-0.1143	0.0099	4.36e-31	0.207	2370000000000.00	-0.0135	0.0645	0.8340	0.165	Yes	Aligned
ETV7	rs36114222	A	G	0.3011	0.0267	1.55e-29	0.030	48478990425.00	0.2008	0.2588	0.4378	0.009	No	Aligned
ETV7	rs145537395	A	G	-0.3509	0.0327	6.81e-27	0.019	24817772402.00	0.0849	0.1997	0.6708	0.015	No	Aligned
ETV7	rs113241919	C	T	-0.2508	0.0248	4.15e-24	0.031	48094117671.00	-0.1407	0.1071	0.1889	0.053	No	Aligned
ETV7	rs111414867	T	C	0.2463	0.0260	3.28e-21	0.031	75300090096.00	-0.0051	0.1564	0.9742	0.026	No	Aligned
ETV7	rs116616466	G	A	0.3258	0.0357	6.94e-20	0.022	43381313497.00	-0.0379	0.1322	0.7742	0.037	No	Aligned
ETV7	rs6457931	G	T	-0.0727	0.0082	5.06e-19	0.577	5580000000000.00	0.0116	0.0513	0.8205	0.330	No	Aligned
ETV7	rs76847127	G	A	0.3883	0.0441	1.26e-18	0.017	30819264526.00	0.2509	0.1483	0.0906	0.029	No	Aligned
ETV7	rs11751412	G	T	-0.0771	0.0090	9.55e-18	0.730	4930000000000.00	-0.0180	0.0505	0.7212	0.350	No	Aligned
ETV7	rs10947603	T	C	-0.0931	0.0109	1.78e-17	0.556	3370000000000.00	-0.0515	0.0495	0.2980	0.469	No	Flipped
ETV7	rs114876533	A	G	-0.4430	0.0527	4.21e-17	0.014	14864698805.00	0.0472	0.2063	0.8189	0.014	No	Aligned
ETV7	rs2071797	T	C	0.1160	0.0151	1.73e-14	0.083	3590000000000.00	0.0094	0.0815	0.9078	0.100	No	Aligned
ETV7	rs72852324	G	A	0.3399	0.0448	3.23e-14	0.015	41934539021.00	0.0690	0.2193	0.7529	0.013	No	Aligned
ETV7	rs62403700	G	T	-0.1874	0.0250	7.42e-14	0.030	80940418007.00	-0.1964	0.1272	0.1226	0.038	No	Aligned
ETV7	rs6457942	T	C	-0.0611	0.0082	1.05e-13	0.383	7600000000000.00	-0.0300	0.0504	0.5519	0.344	No	Aligned
ETV7	rs79809	G	A	0.0981	0.0137	9.61e-13	0.094	5130000000	-0.1568	0.0768	0.0411	0.111	No	Aligned

	702							00.00						
ETV7	rs611831	C	G	-0.2238	0.0316	1.53e-12	0.024	55948342716.00	0.4898	0.2885	0.0896	0.007	Yes	Flipped
ETV7	rs697749	G	A	0.0577	0.0086	2.25e-11	0.311	1520000000000.00	-0.0339	0.0539	0.5302	0.274	No	Flipped
ETV7	rs4236051	T	C	-0.2110	0.0319	3.60e-11	0.027	61920493827.00	0.5141	0.1995	0.0100	0.015	No	Aligned
ETV7	rs185562809	A	C	-0.2788	0.0430	8.80e-11	0.018	35272184845.00	0.0689	0.1338	0.6065	0.034	No	Aligned
ETV7	rs138694427	A	G	0.2248	0.0348	1.11e-10	0.018	101000000000.00	-0.1072	0.1562	0.4924	0.024	No	Aligned
ETV7	rs76173736	T	C	0.2530	0.0397	1.82e-10	0.016	80266012141.00	0.1727	0.1771	0.3294	0.020	No	Aligned
ETV7	rs236461	G	A	-0.0505	0.0080	2.60e-10	0.487	1070000000000.00	0.0232	0.0491	0.6368	0.599	No	Flipped
ETV7	rs148260186	A	C	0.1554	0.0256	1.29e-09	0.028	217000000000.00	0.0398	0.1322	0.7636	0.035	No	Aligned
ETV7	rs142665774	C	T	-0.1565	0.0262	2.34e-09	0.027	109000000000.00	-0.2531	0.1735	0.1446	0.019	No	Aligned
ETV7	rs933235	A	C	0.0650	0.0110	3.72e-09	0.155	1250000000000.00	-0.0029	0.0715	0.9672	0.129	No	Aligned
ETV7	rs142390964	G	A	-0.1899	0.0334	1.27e-08	0.017	73280500875.00	-0.2490	0.2695	0.3555	0.008	No	Aligned
ETV7	rs12526665	A	C	0.0628	0.0111	1.36e-08	0.154	1360000000000.00	-0.0755	0.0738	0.3063	0.121	No	Aligned
ETV7	rs79601514	T	C	-0.2799	0.0497	1.79e-08	0.063	33623786024.00	0.0520	0.0920	0.5722	0.074	No	Aligned
ETV7	rs75658816	G	T	0.1359	0.0243	2.11e-08	0.033	293000000000.00	0.0397	0.1816	0.8267	0.018	No	Aligned
ETV7	rs80017742	T	C	-0.1369	0.0245	2.35e-08	0.030	140000000000.00	0.0688	0.1927	0.7210	0.016	No	Aligned
ETV7	rs80017742	T	C	-0.1369	0.0245	2.35e-08	0.030	140000000000.00	0.0688	0.1927	0.7210	0.016	No	Aligned
IGFBP7	rs2271808	T	C	0.2082	0.0082	3.10e-141	0.624	91299805509.00	-0.0429	0.0505	0.3954	0.341	No	Aligned
IGFBP7	rs4865181	A	G	0.2250	0.0105	2.59e-101	0.173	79370663875.00	-0.1003	0.0638	0.1159	0.169	No	Aligned
IGFBP7	rs7673762	T	G	0.1630	0.0086	7.92e-80	0.313	153000000000.00	0.0002	0.0516	0.9973	0.312	No	Aligned
IGFBP7	rs67057259	G	A	-0.1615	0.0114	1.70e-45	0.145	122000000000.00	0.0538	0.0613	0.3802	0.191	No	Aligned
IGFBP7	rs13118410	C	T	0.1375	0.0105	4.16e-39	0.180	227000000000.00	-0.0699	0.0600	0.2434	0.204	No	Flipped
IG	rs75	G	T	-	0.01	6.04	0.085	86193	0.2229	0.10	0.04	0.053	No	Align

FBP7	882609			0.1908	50	e-37		765716.00		91	11			ed
IGFBP7	rs4865175	A	G	0.2254	0.0185	3.00e-34	0.050	85310548385.00	-0.1944	0.0988	0.0492	0.062	No	Aligned
IGFBP7	rs1277271	C	T	-0.0912	0.0080	8.99e-30	0.561	3710000000.00	0.0240	0.0479	0.6167	0.470	No	Aligned
IGFBP7	rs781561	G	A	0.0891	0.0080	7.44e-29	0.489	5560000000.00	-0.1238	0.0483	0.0103	0.507	No	Flipped
IGFBP7	rs1713949	T	C	-0.1350	0.0139	2.06e-22	0.091	1650000000.00	-0.0412	0.0891	0.6439	0.079	No	Flipped
IGFBP7	rs1713981	G	A	0.0774	0.0083	1.80e-20	0.356	7670000000.00	-0.0673	0.0491	0.1705	0.389	No	Flipped
IGFBP7	rs55987498	T	C	0.1099	0.0126	3.61e-18	0.114	3860000000.00	-0.0663	0.0665	0.3184	0.154	No	Aligned
IGFBP7	rs1718840	A	G	-0.2084	0.0241	4.90e-18	0.031	67569545495.00	-0.0560	0.0863	0.5161	0.086	No	Aligned
IGFBP7	rs7697699	A	G	0.1083	0.0126	8.40e-18	0.113	3990000000.00	-0.0769	0.0679	0.2571	0.146	No	Aligned
IGFBP7	rs1713963	A	G	0.0720	0.0085	1.86e-17	0.331	9040000000.00	-0.0402	0.0487	0.4091	0.412	No	Flipped
IGFBP7	rs115510849	A	G	0.1593	0.0221	5.81e-13	0.038	1940000000.00	0.1632	0.1687	0.3334	0.021	No	Aligned
IGFBP7	rs59562605	T	C	-0.0962	0.0137	2.45e-12	0.096	3020000000.00	-0.0957	0.0697	0.1700	0.137	No	Aligned
IGFBP7	rs6814936	A	G	0.1297	0.0187	3.67e-12	0.054	2960000000.00	-0.0441	0.1118	0.6934	0.049	No	Aligned
IGFBP7	rs11573121	T	G	0.1371	0.0201	9.81e-12	0.046	2670000000.00	0.0339	0.0917	0.7116	0.074	No	Aligned
IGFBP7	rs7677987	A	G	-0.0565	0.0093	1.24e-09	0.244	8450000000.00	0.0783	0.0564	0.1652	0.235	No	Aligned
IGFBP7	rs116906920	T	C	-0.0907	0.0151	1.78e-09	0.911	3260000000.00	0.0498	0.0778	0.5219	0.110	No	Aligned
IGFBP7	rs75473212	G	A	-0.2024	0.0343	3.75e-09	0.018	65182658244.00	0.2984	0.1769	0.0917	0.019	No	Aligned
IGFBP7	rs34862525	T	C	-0.0802	0.0139	7.98e-09	0.094	4120000000.00	-0.2320	0.0993	0.0195	0.063	No	Aligned
IGFBP7	rs35867715	C	T	-0.0587	0.0102	9.25e-09	0.773	7690000000.00	0.0610	0.0566	0.2811	0.243	No	Aligned
IGFBP7	rs111757556	T	C	-0.0695	0.0121	1.03e-08	0.125	5480000000.00	-0.0366	0.0776	0.6376	0.112	No	Aligned
IGFBP7	rs114150987	C	A	0.0797	0.0140	1.39e-08	0.089	8460000000.00	-0.0591	0.0754	0.4337	0.114	No	Aligned
IGFBP7	rs62309920	G	T	-0.1655	0.0299	3.27e-08	0.024	95602150345.00	0.1581	0.1943	0.4159	0.016	No	Aligned
KY	rs78	T	C	0.819	0.02	0.00	0.035	57443	0.0901	0.12	0.45	0.040	No	Align

NU	289644			0	17	e+00		24419.00		15	85			ed
KYNU	rs67414488	G	A	0.3181	0.0090	5.06e-276	0.270	38201992827.00	0.0926	0.0519	0.0746	0.306	No	Flipped
KYNU	rs115031185	A	C	0.9754	0.0393	1.08e-135	0.017	4167523017.00	0.4780	0.3895	0.2197	0.004	No	Aligned
KYNU	rs351678	A	C	0.1511	0.0082	2.64e-76	0.614	179000000000.00	0.0818	0.0482	0.0897	0.476	No	Aligned
KYNU	rs12472023	G	A	-0.1421	0.0093	6.34e-53	0.758	159000000000.00	0.0193	0.0549	0.7248	0.260	No	Aligned
KYNU	rs10496936	T	C	-0.2527	0.0195	1.44e-38	0.054	49281442423.00	0.0040	0.1112	0.9714	0.049	No	Aligned
KYNU	rs140005306	G	A	0.1784	0.0197	1.23e-19	0.044	145000000000.00	0.0602	0.0825	0.4655	0.093	No	Aligned
KYNU	rs77035103	C	A	-0.1801	0.0199	1.36e-19	0.045	91327886142.00	-0.1333	0.0796	0.0942	0.103	No	Aligned
KYNU	rs192182498	G	T	0.2694	0.0320	4.01e-17	0.018	64787132342.00	0.3615	0.1996	0.0701	0.015	No	Aligned
KYNU	rs34232804	G	A	0.0998	0.0124	1.03e-15	0.115	478000000000.00	0.0006	0.0845	0.9942	0.087	No	Aligned
KYNU	rs6718467	T	C	-0.0942	0.0127	1.17e-13	0.131	320000000000.00	-0.0404	0.0645	0.5316	0.171	No	Flipped
KYNU	rs115923597	A	G	0.1819	0.0247	1.62e-13	0.034	148000000000.00	-0.0509	0.1191	0.6693	0.043	No	Aligned
KYNU	rs111416788	A	G	0.1776	0.0241	1.80e-13	0.028	155000000000.00	0.1528	0.1775	0.3894	0.018	No	Aligned
KYNU	rs75004332	G	A	-0.1528	0.0216	1.42e-12	0.036	120000000000.00	-0.3343	0.1834	0.0683	0.018	No	Aligned
KYNU	rs75254563	C	T	0.1982	0.0304	6.97e-11	0.020	130000000000.00	-0.0625	0.1543	0.6856	0.025	No	Aligned
KYNU	rs4609990	G	A	0.1745	0.0280	4.40e-10	0.026	170000000000.00	-0.2151	0.4704	0.6474	0.003	No	Aligned
KYNU	rs148841722	T	G	-0.1613	0.0264	9.85e-10	0.027	104000000000.00	-0.0792	0.2140	0.7112	0.013	No	Aligned
KYNU	rs116636300	G	A	0.2475	0.0423	5.02e-09	0.013	86725872259.00	0.1202	0.3271	0.7132	0.006	No	Aligned
KYNU	rs706633	A	G	0.0489	0.0086	1.53e-08	0.311	2250000000000.00	0.0875	0.0489	0.0738	0.421	No	Flipped
KYNU	rs114063617	C	A	0.1440	0.0255	1.72e-08	0.031	260000000000.00	-0.1527	0.2051	0.4565	0.014	No	Aligned
MUC20	rs2688492	C	T	0.4918	0.0099	0.00e+00	0.259	15725832431.00	0.0483	0.0518	0.3512	0.315	No	Flipped

MUC20	rs34697430	T	C	0.3965	0.0096	0.00e+00	0.490	24403534296.00	0.0182	0.0486	0.7085	0.542	No	Flipped
MUC20	rs35845795	A	C	0.5172	0.0115	0.00e+00	0.172	14281924091.00	0.0243	0.0592	0.6813	0.206	No	Aligned
MUC20	rs78923543	G	C	0.6958	0.0172	0.00e+00	0.083	7929701511.00	-0.0242	0.0778	0.7553	0.107	Yes	Aligned
MUC20	rs2550252	C	T	0.3342	0.0095	8.83e-272	0.424	34643001173.00	0.0683	0.0491	0.1645	0.386	No	Flipped
MUC20	rs67175307	T	C	-0.5587	0.0229	6.81e-131	0.044	10794336645.00	-0.0691	0.0923	0.4540	0.076	No	Aligned
MUC20	rs7633151	A	G	0.2224	0.0108	5.18e-94	0.528	81569889658.00	0.0712	0.0481	0.1390	0.489	No	Flipped
MUC20	rs4927707	T	C	-0.2053	0.0101	4.00e-91	0.292	78659687937.00	-0.0300	0.0513	0.5582	0.333	No	Flipped
MUC20	rs61306983	C	T	-0.2622	0.0136	1.44e-82	0.119	48015451390.00	-0.0666	0.0746	0.3717	0.117	No	Aligned
MUC20	rs73212254	T	C	-0.2893	0.0151	1.43e-81	0.884	39396784032.00	-0.0128	0.0685	0.8524	0.146	No	Aligned
MUC20	rs35270284	T	C	0.2104	0.0110	4.64e-81	0.805	91830591850.00	0.0412	0.0589	0.4843	0.217	No	Aligned
MUC20	rs79229103	T	C	0.7237	0.0382	4.38e-80	0.020	7770332567.00	0.1265	0.1438	0.3791	0.029	No	Aligned
MUC20	rs4927708	C	T	0.1701	0.0091	1.14e-78	0.598	141000000000.00	-0.0130	0.0489	0.7910	0.447	No	Aligned
MUC20	rs9819459	T	C	-0.2105	0.0121	2.43e-67	0.753	73676174314.00	-0.0037	0.0538	0.9449	0.277	No	Aligned
MUC20	rs4677688	T	C	0.1606	0.0093	6.77e-67	0.563	159000000000.00	-0.0577	0.0485	0.2339	0.453	No	Aligned
MUC20	rs41298099	C	T	0.5496	0.0325	5.10e-64	0.025	13658807569.00	-0.0358	0.1278	0.7793	0.038	No	Aligned
MUC20	rs115645306	T	C	0.6925	0.0418	1.28e-61	0.017	8625482718.00	0.1004	0.1276	0.4313	0.038	No	Aligned
MUC20	rs4927711	T	C	-0.1639	0.0101	1.37e-59	0.352	121000000000.00	-0.0336	0.0503	0.5051	0.370	No	Aligned
MUC20	rs11916483	C	T	0.4626	0.0286	1.07e-58	0.027	19390566915.00	0.0314	0.1412	0.8239	0.030	No	Aligned
MUC20	rs72611105	C	T	0.2979	0.0203	8.21e-49	0.943	47381611460.00	-0.0817	0.1030	0.4273	0.057	No	Aligned
MUC20	rs11922971	A	G	-0.1918	0.0133	2.98e-47	0.853	86788930945.00	-0.0583	0.0654	0.3727	0.167	No	Aligned
MUC20	rs76201019	T	C	-0.4249	0.0312	3.11e-42	0.026	17555040271.00	-0.1702	0.2083	0.4137	0.014	No	Aligned
MUC20	rs2241413	C	T	0.1288	0.0095	8.08e-42	0.463	256000000000.00	0.0817	0.0483	0.0908	0.471	No	Aligned
MUC20	rs6804171	A	G	0.1295	0.0104	1.04e-35	0.514	257000000000.00	0.0563	0.0494	0.2544	0.520	No	Aligned

MUC20	rs147508067	C	T	0.5683	0.0484	8.18e-32	0.019	13508352303.00	0.3414	0.2113	0.1062	0.013	No	Aligned
MUC20	rs115743766	A	G	0.4016	0.0346	3.32e-31	0.021	27108271000.00	0.2314	0.1613	0.1515	0.023	No	Aligned
MUC20	rs7610708	T	G	0.1320	0.0117	2.06e-29	0.212	2520000000000.00	-0.0567	0.0568	0.3188	0.243	No	Aligned
MUC20	rs11927474	C	A	-0.1540	0.0139	2.19e-28	0.166	1290000000000.00	-0.0650	0.0623	0.2974	0.185	No	Aligned
MUC20	rs76863178	C	T	-0.3010	0.0287	1.15e-25	0.032	33581355295.00	0.0479	0.1506	0.7503	0.027	No	Aligned
MUC20	rs77106593	A	G	-0.4314	0.0421	1.35e-24	0.015	16287528501.00	0.0914	0.1665	0.5830	0.022	No	Aligned
MUC20	rs77176301	C	T	-0.2400	0.0235	1.83e-24	0.042	52591969233.00	0.1207	0.1605	0.4519	0.022	No	Aligned
MUC20	rs116854542	A	C	-0.6112	0.0623	1.03e-22	0.028	8050691882.00	0.0122	0.1171	0.9169	0.046	No	Aligned
MUC20	rs56260729	A	G	0.1069	0.0111	5.51e-22	0.208	3980000000000.00	0.0703	0.0560	0.2096	0.248	No	Aligned
MUC20	rs140843768	T	C	0.3192	0.0334	1.29e-21	0.022	44724902174.00	0.1128	0.1546	0.4653	0.025	No	Aligned
MUC20	rs7627155	T	C	0.1157	0.0121	1.61e-21	0.151	3410000000000.00	0.0835	0.0727	0.2507	0.125	No	Aligned
MUC20	rs143060720	T	C	-0.4332	0.0455	1.75e-21	0.016	15938074849.00	-0.2044	0.1807	0.2580	0.018	No	Aligned
MUC20	rs13066800	G	A	0.2245	0.0239	5.55e-21	0.048	90748044361.00	0.1385	0.0969	0.1528	0.066	No	Aligned
MUC20	rs147180676	G	T	-0.2807	0.0299	6.65e-21	0.028	37849219638.00	-0.0646	0.1493	0.6656	0.027	No	Aligned
MUC20	rs147180676	G	T	-0.2807	0.0299	6.65e-21	0.028	37849219638.00	-0.0646	0.1493	0.6656	0.027	No	Aligned
MUC20	rs60656840	G	A	-0.0997	0.0107	1.04e-20	0.211	3000000000000.00	-0.0005	0.0624	0.9935	0.182	No	Aligned
MUC20	rs115160556	T	C	0.3335	0.0370	2.01e-19	0.019	41546481771.00	0.0895	0.1592	0.5740	0.024	No	Aligned
MUC20	rs146159581	C	T	0.1177	0.0134	1.38e-18	0.155	3350000000000.00	0.0258	0.0617	0.6754	0.197	No	Aligned
MUC20	rs150297461	C	A	0.3431	0.0391	1.57e-18	0.020	39492580369.00	-0.0232	0.2117	0.9128	0.013	No	Aligned
MUC20	rs34049168	A	G	0.1088	0.0127	1.16e-17	0.203	3950000000000.00	0.0514	0.0599	0.3913	0.222	No	Aligned

MUC20	rs7432894	C	T	-0.0782	0.0093	3.78e-17	0.328	4770000000.00	0.0203	0.0540	0.7070	0.272	No	Flipped
MUC20	rs60568302	A	G	0.2015	0.0244	1.57e-16	0.034	1160000000.00	0.1056	0.1363	0.4383	0.033	No	Aligned
MUC20	rs151192980	G	A	0.2122	0.0259	2.43e-16	0.036	1050000000.00	0.0950	0.1959	0.6277	0.016	No	Aligned
MUC20	rs4677802	G	A	0.0830	0.0102	3.21e-16	0.686	6880000000.00	0.0535	0.0541	0.3226	0.272	No	Flipped
MUC20	rs28651009	T	C	0.0781	0.0097	7.99e-16	0.356	7800000000.00	-0.0144	0.0508	0.7765	0.365	No	Flipped
MUC20	rs11712192	A	G	-0.0911	0.0117	6.18e-15	0.207	3450000000.00	-0.0216	0.0573	0.7065	0.232	No	Aligned
MUC20	rs78961457	G	A	-0.1540	0.0198	6.94e-15	0.054	1210000000.00	0.0449	0.1217	0.7120	0.041	No	Aligned
MUC20	rs7630048	A	G	0.0723	0.0094	1.17e-14	0.326	9230000000.00	-0.0009	0.0550	0.9874	0.260	No	Flipped
MUC20	rs115786032	T	C	0.3526	0.0462	2.38e-14	0.015	38902913938.00	-0.0372	0.2804	0.8945	0.007	No	Aligned
MUC20	rs68143103	A	G	0.2823	0.0371	2.75e-14	0.020	60721152241.00	-0.0255	0.2731	0.9255	0.008	No	Aligned
MUC20	rs823532	C	T	0.0783	0.0105	7.87e-14	0.672	7930000000.00	0.0035	0.0498	0.9447	0.383	No	Aligned
MUC20	rs35559909	G	A	0.1205	0.0165	2.46e-13	0.093	3370000000.00	-0.0789	0.0746	0.2903	0.121	No	Aligned
MUC20	rs7340710	T	C	-0.1183	0.0162	2.84e-13	0.079	2020000000.00	-0.1592	0.0812	0.0501	0.096	No	Aligned
MUC20	rs146105953	C	T	0.3706	0.0515	6.40e-13	0.019	35870273219.00	0.1755	0.1428	0.2190	0.029	No	Aligned
MUC20	rs112242983	A	C	-0.1059	0.0149	1.41e-12	0.094	2500000000.00	0.0429	0.0862	0.6190	0.086	No	Aligned
MUC20	rs114126286	C	T	-0.3701	0.0539	6.43e-12	0.015	20316620193.00	0.2233	0.2085	0.2841	0.014	No	Aligned
MUC20	rs4927887	T	C	0.0648	0.0099	5.49e-11	0.359	12100000000.00	-0.0362	0.0491	0.4617	0.406	No	Aligned
MUC20	rs2056469	G	A	-0.0978	0.0151	8.46e-11	0.111	2870000000.00	-0.0771	0.0858	0.3687	0.086	No	Aligned
MUC20	rs9876236	A	G	0.2157	0.0335	1.19e-10	0.025	1100000000.00	0.0865	0.1920	0.6522	0.016	No	Aligned
MUC20	rs2686442	G	A	0.0920	0.0143	1.20e-10	0.299	6050000000.00	0.1375	0.0525	0.0088	0.346	No	Aligned
MUC20	rs80087324	T	C	-0.2204	0.0344	1.45e-10	0.022	56238859583.00	-0.2108	0.2183	0.3342	0.013	No	Aligned
MUC	rs24108	G	A	-0.199	0.0318	3.53e-10	0.023	6822660780	0.0058	0.1407	0.9669	0.030	No	Aligned

20	02			5				5.00						
MUC20	rs146199320	G	A	0.1684	0.0270	4.40e-10	0.032	1830000000000.00	0.0050	0.1781	0.9774	0.019	No	Aligned
MUC20	rs9851595	T	C	0.0563	0.0091	6.42e-10	0.628	1640000000000.00	0.0466	0.0491	0.3426	0.400	No	Aligned
MUC20	rs9864994	A	C	0.0573	0.0093	7.64e-10	0.663	1590000000000.00	-0.0028	0.0527	0.9577	0.302	No	Aligned
MUC20	rs62283270	C	T	0.0782	0.0127	8.68e-10	0.654	853000000000.00	-0.0147	0.0515	0.7760	0.357	No	Aligned
MUC20	rs73206633	G	A	-0.0770	0.0128	1.85e-09	0.866	452000000000.00	0.0165	0.0712	0.8162	0.131	No	Aligned
MUC20	rs56109345	G	A	-0.0533	0.0090	3.05e-09	0.611	942000000000.00	0.0395	0.0496	0.4258	0.369	No	Aligned
MUC20	rs114439325	A	G	-0.3336	0.0565	3.56e-09	0.022	23992316511.00	0.1400	0.1335	0.2944	0.035	No	Aligned
MUC20	rs55824736	T	C	-0.2334	0.0398	4.37e-09	0.022	48947617144.00	-0.2163	0.1437	0.1321	0.030	No	Aligned
MUC20	rs34288633	C	T	-0.0763	0.0130	4.61e-09	0.232	458000000000.00	-0.0578	0.0674	0.3910	0.160	No	Aligned
MUC20	rs79398212	C	T	0.1189	0.0203	4.65e-09	0.052	376000000000.00	-0.0293	0.1235	0.8126	0.040	No	Aligned
MUC20	rs116492543	T	C	-0.2034	0.0358	1.35e-08	0.025	63817297682.00	-0.1004	0.1106	0.3642	0.051	No	Aligned
MUC20	rs1056726	A	G	0.1362	0.0242	1.84e-08	0.042	291000000000.00	0.0426	0.1278	0.7388	0.038	No	Aligned
MUC20	rs78292793	T	C	-0.1195	0.0213	2.10e-08	0.052	184000000000.00	-0.1566	0.1072	0.1440	0.055	No	Aligned
MUC20	rs141363947	A	G	-0.3309	0.0596	2.80e-08	0.022	23945734153.00	0.2971	0.1510	0.0491	0.027	No	Aligned
MUC20	rs62408947	A	G	-0.0824	0.0149	3.01e-08	0.095	386000000000.00	0.0115	0.0763	0.8806	0.115	No	Aligned
MUC20	rs9870813	C	T	0.0485	0.0088	3.88e-08	0.409	2320000000000.00	-0.0459	0.0491	0.3498	0.422	No	Flipped
MUC20	rs6806773	A	G	-0.0570	0.0105	4.86e-08	0.224	801000000000.00	-0.0486	0.0529	0.3580	0.296	No	Flipped
THBS1	rs1822105	C	T	0.3244	0.0080	0.00e+00	0.568	36473883894.00	0.0466	0.0483	0.3346	0.461	No	Aligned
THBS1	rs12909502	A	G	-0.2708	0.0080	1.34e-252	0.532	47003838494.00	0.0626	0.0479	0.1917	0.456	No	Flipped
THBS1	rs79387284	T	C	0.8689	0.0257	2.35e-251	0.974	5135765379.00	-0.0218	0.1642	0.8945	0.022	No	Aligned
THBS1	rs16968876	C	T	-0.2934	0.0095	3.78e-210	0.227	39795583239.00	0.0193	0.0549	0.7248	0.255	No	Flipped
THBS	rs18476	G	A	-0.250	0.0130	8.64e-83	0.105	5268132988	0.2501	0.1073	0.0197	0.053	No	Aligned

1	63			3				0.00						
THBS1	rs156658	G	A	-0.2403	0.0130	7.80e-77	0.105	56937102932.00	0.1372	0.0869	0.1143	0.083	No	Flipped
THBS1	rs4924347	T	C	-0.2333	0.0136	5.08e-66	0.095	59927681820.00	0.0261	0.0823	0.7510	0.095	No	Aligned
THBS1	rs149334770	T	G	0.6053	0.0359	1.24e-63	0.017	11265114052.00	0.0054	0.4024	0.9894	0.004	No	Aligned
THBS1	rs17614000	T	C	0.2668	0.0188	1.35e-45	0.047	59367923731.00	0.0280	0.0692	0.6854	0.140	No	Aligned
THBS1	rs62006528	G	A	0.1979	0.0146	4.43e-42	0.096	10900000000.00	0.0349	0.0664	0.5996	0.159	No	Aligned
THBS1	rs8026892	C	T	0.1441	0.0109	3.23e-40	0.159	20600000000.00	-0.0655	0.0581	0.2596	0.220	No	Aligned
THBS1	rs276854	A	G	-0.1558	0.0119	1.95e-39	0.154	13000000000.00	0.0582	0.0700	0.4058	0.138	No	Aligned
THBS1	rs8039714	A	C	0.2098	0.0162	1.59e-38	0.068	97402860478.00	-0.0337	0.0806	0.6762	0.102	No	Aligned
THBS1	rs12904023	A	G	0.1613	0.0136	2.37e-32	0.094	16800000000.00	-0.0580	0.0893	0.5162	0.077	No	Aligned
THBS1	rs59122632	C	T	-0.1920	0.0169	8.60e-30	0.059	83640568013.00	-0.0009	0.0932	0.9925	0.072	No	Aligned
THBS1	rs112833594	C	T	-0.2760	0.0275	9.57e-24	0.024	39656121238.00	0.0520	0.1655	0.7533	0.021	No	Aligned
THBS1	rs77660997	A	G	0.2995	0.0304	6.51e-23	0.023	50419615210.00	-0.1143	0.1024	0.2644	0.061	No	Aligned
THBS1	rs150751687	T	C	-0.3474	0.0362	9.32e-22	0.016	24811409031.00	-0.0891	0.2008	0.6572	0.015	No	Aligned
THBS1	rs75542157	T	G	-0.3046	0.0334	7.88e-20	0.018	31964857212.00	0.0988	0.2446	0.6862	0.010	No	Aligned
THBS1	rs6492914	G	A	-0.0724	0.0080	9.76e-20	0.469	56600000000.00	0.0655	0.0478	0.1707	0.482	No	Flipped
THBS1	rs156795	A	G	-0.0713	0.0082	2.34e-18	0.519	57800000000.00	0.0406	0.0486	0.4035	0.538	No	Aligned
THBS1	rs8027821	C	A	-0.0674	0.0085	1.62e-15	0.350	63500000000.00	0.0160	0.0518	0.7572	0.324	No	Aligned
THBS1	rs276856	A	G	-0.0634	0.0084	5.67e-14	0.445	70800000000.00	0.0191	0.0480	0.6899	0.479	No	Aligned
THBS1	rs11629850	A	G	-0.0575	0.0080	5.14e-13	0.509	85200000000.00	-0.0964	0.0478	0.0434	0.499	No	Aligned
THBS1	rs17621729	A	G	-0.1510	0.0216	2.63e-12	0.038	12300000000.00	0.1909	0.1653	0.2482	0.022	No	Aligned
THBS1	rs116952056	T	C	0.1482	0.0226	5.43e-11	0.038	23200000000.00	-0.1433	0.1002	0.1527	0.062	No	Aligned
THBS	rs80377	C	T	-0.167	0.0258	7.08e-11	0.025	9731463487	0.1465	0.1188	0.2176	0.043	No	Aligned

1	33			9				9.00						
THBS1	rs74869668	C	T	-0.1991	0.0307	9.15e-11	0.019	69115881495.00	0.4104	0.1743	0.0186	0.019	No	Aligned
THBS1	rs8035543	A	G	-0.0762	0.0118	1.14e-10	0.133	471000000000.00	0.0359	0.0724	0.6200	0.125	No	Aligned
THBS1	rs117681776	A	G	-0.1821	0.0289	2.95e-10	0.023	81993880105.00	-0.0431	0.1061	0.6845	0.054	No	Aligned
THBS1	rs17721122	T	A	0.0777	0.0123	3.13e-10	0.882	856000000000.00	-0.1077	0.0895	0.2287	0.078	Yes	Aligned
THBS1	rs72725044	A	G	-0.2098	0.0334	3.56e-10	0.019	61721358968.00	0.1437	0.3426	0.6748	0.005	No	Aligned
THBS1	rs1865755	C	A	0.1019	0.0167	9.89e-10	0.939	503000000000.00	-0.1314	0.0981	0.1802	0.064	No	Aligned
THBS1	rs10520147	G	A	-0.0918	0.0151	1.35e-09	0.075	319000000000.00	0.0123	0.0979	0.9002	0.064	No	Aligned
THBS1	rs77821708	G	A	-0.1030	0.0175	3.95e-09	0.064	251000000000.00	0.1060	0.1038	0.3070	0.057	No	Aligned
THBS1	rs62002527	T	C	0.0561	0.0098	8.90e-09	0.213	1700000000000.00	0.0579	0.0613	0.3453	0.188	No	Aligned
THBS1	rs117210124	A	G	0.1075	0.0190	1.48e-08	0.052	466000000000.00	-0.0127	0.1228	0.9174	0.040	No	Aligned
THBS1	rs1106508	G	A	0.0571	0.0101	1.58e-08	0.762	1650000000000.00	-0.0253	0.0540	0.6395	0.273	No	Aligned
VSIG10	rs4128858	A	G	-0.4126	0.0088	0.00e+00	0.411	20558458577.00	0.1107	0.0483	0.0219	0.447	No	Aligned
VSIG10	rs4590911	C	T	-0.2258	0.0080	3.61e-177	0.475	66857295213.00	0.0155	0.0482	0.7481	0.553	No	Aligned
VSIG10	rs10850953	G	T	0.2404	0.0101	7.33e-125	0.203	68884120685.00	-0.0561	0.0622	0.3672	0.181	No	Aligned
VSIG10	rs492939	A	G	0.1251	0.0080	9.33e-56	0.525	266000000000.00	0.0332	0.0482	0.4904	0.459	No	Aligned
VSIG10	rs12830847	T	G	0.1424	0.0091	1.08e-54	0.262	206000000000.00	-0.0166	0.0531	0.7552	0.291	No	Aligned
VSIG10	rs78708372	T	C	0.2147	0.0139	6.28e-54	0.095	90589113308.00	-0.1291	0.0713	0.0704	0.134	No	Aligned
VSIG10	rs73220128	G	A	-0.1730	0.0115	8.77e-51	0.137	107000000000.00	0.1069	0.0715	0.1350	0.128	No	Aligned
VSIG10	rs149138610	T	C	-0.2479	0.0188	1.09e-39	0.048	51319410990.00	-0.0081	0.0909	0.9291	0.076	No	Aligned
VSIG10	rs2393373	T	C	-0.1003	0.0085	3.03e-32	0.342	309000000000.00	0.0303	0.0512	0.5534	0.335	No	Flipped
VSIG10	rs7486166	G	A	-0.1304	0.0113	1.33e-30	0.149	182000000000.00	0.0478	0.0716	0.5043	0.128	No	Aligned
VSIG10	rs7135649	A	G	-0.1644	0.0145	6.48e-30	0.084	114000000000.00	-0.1931	0.0851	0.0233	0.087	No	Aligned

VSIG10	rs35019804	T	C	0.3487	0.0316	2.51e-28	0.022	36321649181.00	0.0927	0.1452	0.5233	0.028	No	Aligned
VSIG10	rs142225588	A	G	0.1613	0.0149	2.55e-27	0.082	17000000000.00	0.0976	0.0887	0.2710	0.080	No	Aligned
VSIG10	rs150073905	G	A	0.3357	0.0311	3.60e-27	0.020	39349026853.00	0.0586	0.2342	0.8026	0.011	No	Aligned
VSIG10	rs1503768	T	C	-0.1097	0.0115	1.00e-21	0.172	24900000000.00	0.0094	0.0554	0.8648	0.275	No	Aligned
VSIG10	rs12582265	A	G	-0.0996	0.0107	1.50e-20	0.165	30000000000.00	0.0919	0.0705	0.1920	0.132	No	Aligned
VSIG10	rs2254036	G	A	0.1691	0.0182	1.97e-20	0.054	16100000000.00	-0.1144	0.1093	0.2952	0.050	No	Flipped
VSIG10	rs61943491	A	G	-0.2375	0.0264	2.50e-19	0.028	52425975549.00	0.0430	0.1196	0.7194	0.044	No	Aligned
VSIG10	rs76148053	A	G	0.1888	0.0216	2.49e-18	0.038	13100000000.00	-0.2372	0.1476	0.1080	0.028	No	Aligned
VSIG10	rs77533092	T	C	-0.1501	0.0176	1.56e-17	0.056	13000000000.00	-0.0338	0.1131	0.7653	0.047	No	Aligned
VSIG10	rs7301776	A	G	-0.0671	0.0080	3.80e-17	0.476	64800000000.00	0.0338	0.0480	0.4816	0.490	No	Flipped
VSIG10	rs7960802	T	C	-0.1119	0.0134	5.28e-17	0.099	23300000000.00	0.0642	0.0763	0.4006	0.111	No	Aligned
VSIG10	rs149400623	A	G	0.2523	0.0317	1.60e-15	0.020	75028983050.00	0.1182	0.1274	0.3533	0.037	No	Aligned
VSIG10	rs10850940	T	C	0.0782	0.0107	2.48e-13	0.177	80200000000.00	0.0007	0.0577	0.9905	0.237	No	Aligned
VSIG10	rs5745851	C	T	0.2404	0.0330	3.08e-13	0.020	84891414736.00	-0.0348	0.2015	0.8627	0.015	No	Aligned
VSIG10	rs57353139	C	A	0.1060	0.0148	8.20e-13	0.079	43900000000.00	0.0820	0.0871	0.3463	0.083	No	Aligned
VSIG10	rs117354266	T	C	-0.1640	0.0234	2.24e-12	0.035	10400000000.00	-0.0493	0.1059	0.6418	0.055	No	Aligned
VSIG10	rs34026283	T	C	-0.0578	0.0084	4.74e-12	0.368	83400000000.00	0.0499	0.0518	0.3358	0.328	No	Aligned
VSIG10	rs75734538	T	C	0.2337	0.0340	6.62e-12	0.018	91593398530.00	-0.0687	0.1511	0.6492	0.026	No	Aligned
VSIG10	rs73217917	C	T	-0.2456	0.0383	1.50e-10	0.015	45307527376.00	-0.2862	0.2423	0.2375	0.010	No	Aligned
VSIG10	rs10850949	T	G	0.0678	0.0109	4.61e-10	0.837	1130000000000.00	0.0435	0.0609	0.4754	0.191	No	Aligned
VSIG10	rs138890021	T	C	-0.4364	0.0702	5.11e-10	0.014	14224801261.00	-0.0726	0.2602	0.7802	0.009	No	Aligned
VSIG	rs42380	A	G	-0.063	0.0105	1.26e-09	0.262	6650000000	-0.0206	0.0540	0.7033	0.291	No	Aligned

10	54			6				00.00						
VSIG10	rs2723277	A	C	0.1976	0.0337	4.72e-09	0.017	1360000000000.00	0.1140	0.3461	0.7419	0.005	No	Aligned
VSIG10	rs148380960	A	C	-0.2830	0.0498	1.30e-08	0.021	32987115435.00	0.3605	0.3248	0.2670	0.006	No	Aligned
VSIG10	rs11068962	T	G	-0.0750	0.0134	2.41e-08	0.098	46700000000.00	0.1636	0.0766	0.0326	0.110	No	Aligned

Note: Beta_outcome_aligned and EAF_outcome_aligned were aligned to the effect allele at the exposure level (EA_exposure); Allele_alignment = Flipped indicates that the outcome allele is in the opposite direction to the exposure allele.
